# Microwave-Assisted Neutral Glycosylation Reactions in the Absence of Reagent Activators

**DOI:** 10.3390/molecules30183693

**Published:** 2025-09-11

**Authors:** Shanika M. P. Gamage, Geraud Valentin, Samir Ghosh, Pradheep Eradi, Rahul S. Bagul, David Crich, Peter R. Andreana

**Affiliations:** 1 Department of Chemistry and Biochemistry, University of Toledo, Toledo, OH 43606, USAgeraud.valentin@rockets.utoledo.edu (G.V.);; 2 Department of Chemistry and College of Pharmacy, University of Georgia, Athens, GA 30602, USA

**Keywords:** glycosylation, microwave irradiation, reagent-free neutral glycosylation

## Abstract

Use of a microwave-labile leaving group, 2,4-dinitrophenyl (2,4-DNP), leads to *O*-glycosylation in 70–80% yields with high α-selectivity. The 2,4-DNP glycosyl donors, protected with electron-donating benzyl ethers, gave the best results under controlled microwave conditions, which were all conducted in polar aprotic solvents, such as DMF, and were all conducted in the absence of any reagent-activating additives such as a Lewis acid/base or Brønsted–Lowry acid/base. This method represents concerted efforts towards reagent-free neutral glycosylation.

## 1. Introduction

The chemical synthesis of an acetal, linking sugar units together, is of critical importance for moving the field of glycoscience forward. Chemical glycosylation is normally achieved at the electrophilic anomeric position through the reaction between an activatable glycosyl donor and a nucleophilic glycosyl acceptor employing a promoter, such as a Lewis acid, below ambient temperature [1]. Developing a glycosylation reaction that operates under non-acidic conditions is desirable to allow for the formation of acid-sensitive glycosidic linkages in the presence of acid-sensitive protecting groups.

Many recent reports have developed new glycosylation methods aiming for more reliable, robust, and user-friendly alternatives to the stringent conditions required when working with standard promoters, like Lewis acids. For instance, glycosylation with a hydrogen-bond donor catalyst [2], phenanthroline-catalyzed stereoselective glycosylation [3], chelation-assisted glycosylation [4], and enzymatic transformations all attempt to overcome challenges with stereoselectivity and hydrolysis realized during or after glycosylation has occurred. Among many glycosylation methods, a few reports by the groups of Gervay-Hague [5], Jensen [6], Waldmann [7], and others [4,8], describe employing neutral and mildly basic conditions. Of particular interest to us and relevant to the work described herein, the Jensen group, in particular, described a glycosylation method using dinitro salicylate (DISAL) donors. Under conventional heating conditions, they employed the DISAL donor to obtain a glycosylated product at 40–60 °C within 17 h using LiClO_4_ as a mild Lewis acid activator. Having also performed this reaction under microwave irradiation, they were able to reduce the reaction time from hours to minutes at elevated reaction temperatures from 60 °C to 100 °C [6,9,10].

**Figure 1:**
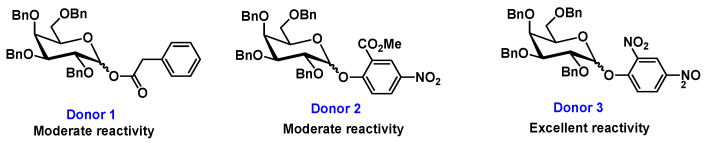
Ester and nitrophenol-based microwave-labile leaving groups.

Cognizant of Jensen’s approach, we set out to target thermal activation of armed [11] sugar donors with the assistance of microwave irradiation but in the absence of any external additives/promoters. We hypothesized that microwave irradiation [12] has the potential to promote thermally driven glycosylation reactions through localized heating in the absence of reagent additives (only solvent), for short reaction times and improved overall yields. Over the past three decades, microwave-assisted chemistry has attracted a considerable amount of attention in the labs of academic and industrial groups, allowing for the discovery of unprecedented chemical reactivities [13,14,15] and efficiency in glycosylations [16,17]. Along these lines, our longstanding interest has been to develop microwave-labile leaving groups (MWLLGs), which can be used in an orthogonal manner with other common protecting groups. With that idea in mind, we have designed and incorporated ester- and nitrophenol-based microwave-labile leaving groups (donors 1–3, Figure 1) and performed glycosylation reactions under microwave irradiation in the absence of any promoters.

## 2. Results

**Scheme 1:**
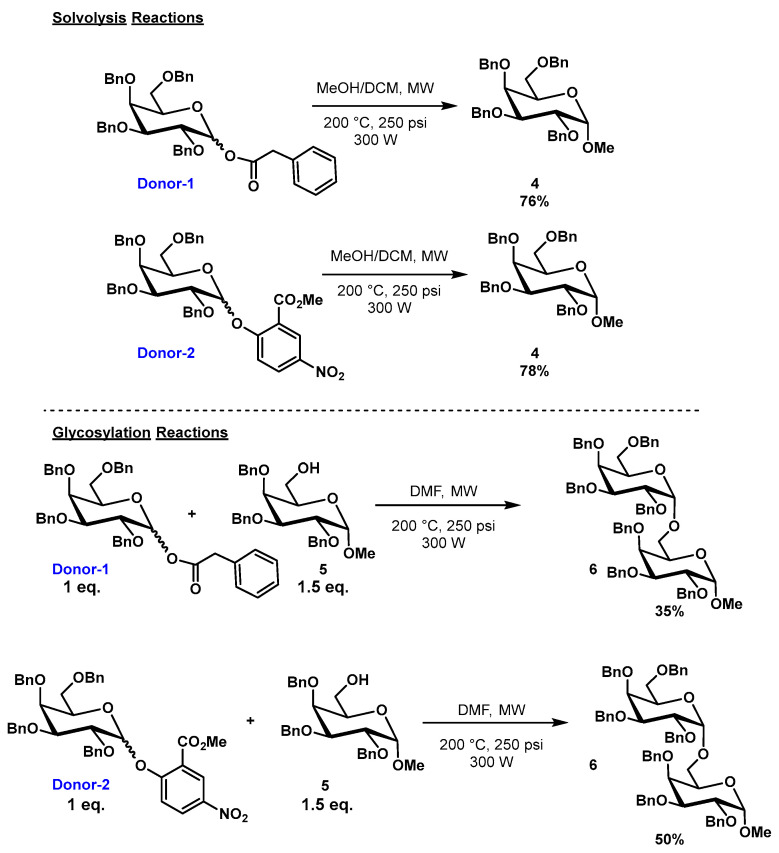
Microwave reaction conditions for glycosylation using sugar donors **1** and **2**.

In order to establish that glycosylation reactions would ensue under our ascribed conditions, a series of preliminary solvolysis experiments were conducted (Scheme 1). We investigated D-galactose-derived donors, which are known for their high reactivity. Both galactosyl phenylacetate (donor 1) and 2-methoxycarbonyl-4-nitrophenyl D-galactoside (donor 2), in a MeOH/DCM = 9:1 solvent system using microwave irradiation at 200 °C, 300 W, and 250 psi, gave methyl glycoside (**4**) in yields above 75% (all synthetic procedures and characterization data are presented in supplementary material). In these solvolysis cases, methanol acted as an acceptor and DCM was simply used to dissolve the donors. After realizing our solvolysis approach, we thought that, for the preparation of a simple disaccharide in our glycosylation system (Scheme 1; glycosylation reactions), the reaction of donor 1 with acceptor (**5**) at 200 °C, 300 W, and 250 psi in DMF would be sufficient. However, in practice, we observed a yield of 35% for compound **6**. The solvent change from MEOH/DCM to DMF is rationalized as follows: DMF, as the polar aprotic solvent with a large dipole moment (*µ* = 3.86 D at 25 °C), high boiling point (153 °C) and dielectric constant (ε = 37.51 at 25 °C), has been recognized as an optimal solvent for microwave-based reactions [18]. However, when we employed DMF as our solvent in our glycosylation reaction with donor 2 and 6-OH D-galactosyl acceptor **5** (Scheme 1), we only observed a 50% yield of compound **6**.

While we were pleased to obtain results noted in Scheme 1, the issue of low yield plagued us, and we decided to replace the anomeric moieties noted in Scheme 1 with more electron-deficient aryl dinitro groups by employing 2,4-DNP donor 3. We elected to employ 2,4-DNP as a glycosyl donor, given its leaving group potential in water but also understanding that it is not reactive enough to undergo glycosylation on its own. To our delight, donor 3 reacted under our prescribed solvolysis conditions and gave methyl glycoside **4** in excellent yields of 81–87% (Table 1; entry 1, 2, 3). In every example conducted, 2,4-dinitrophenol was isolated as the sole by-product (recyclable in our process), which was isolated from the aqueous layer using size exclusion chromatography. Prepared 2,4-DNP donor **3** (see SI) was dissolved in a dry methanol/DCM solution and then subjected to microwave irradiation at 200 °C, 300 W (power), and 250 psi (Table 1). When the reaction was conducted in a 1:1 mixture of **3a** and **3b**, it resulted in 1.3:1 mixture of α and β products (Table 1; entry 3). It is important to note that we observed no reaction with **3a** and **3b** utilizing traditional refluxing conditions (Table 1; entry 4). The reaction pH was monitored and noted to be neutral, even though the by-product of 2,4-dinitrophenol has a pKa ~4. These initial results confirmed that the 2,4-DNP donor was highly susceptible to microwave irradiation under a high temperature and pressure. It was also rewarding to observe that benzylated donors and acceptors did not decompose at high temperature microwave conditions and the 2,4-DNP donor **3** was stable at room temperature without any decomposition over long periods of time (months). These results demonstrated that we could overcome issues with yields and gave us confidence that an entirely neutral glycosylation method (without additives/promoters) employing thermal microwave energy could indeed be realized.

**Figure 2:**
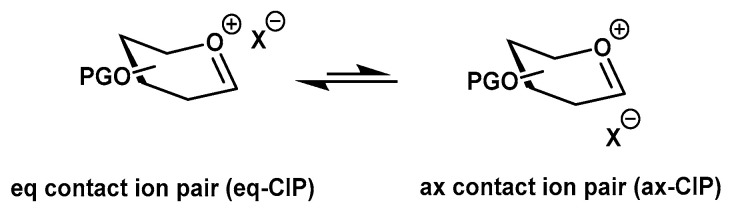
Contact ion pairs.

Entry 2 in Table 1 shows either a mixture of α and β products, indicative of an SN1-like pathway (entry 1), or the stereospecific formation of the α anomer, an S_N_2-like pathway [19,20]. This apparent paradox can be explained by contact ion pairs. While the reaction of **3b** may form the equatorial (eq) contact ion pair (CIP) (assuming that eq-CIP is more reactive than the axial (ax)-CIP) that then forms the exclusive *α* product, donor **3a** may undergo departure of a dinitrophenolate ion and form an ax-CIP which may convert into the more reactive eq-CIP (Figure 2), forming a mixture of *α* and *β* products.

After our initial success, we moved forward to probe the reaction scope with 2,4-DNP D-galactoside and complex acceptors using our microwave conditions. Our investigation commenced with treatment of benzylated 2,4-DNP donor 3 with benzylated 6-OH D-galactosyl acceptor (**5**) using various solvents under microwave conditions at different temperatures. (Table 2). **Donor 3** was separated out into both the **3a** and **3b** anomers, which were treated individually to determine the effect of donor stereochemistry.

A variety of solvents were tested; however, no glycosylation product was obtained when low boiling point and low microwave-absorbing solvents [18] (DCM, THF and ACN) were used (Table 2; entries 1–3). When the solvent was changed to a high boiling point, microwave-absorbing solvent [18] such as DMF, and N-methyl-2-pyrollidone (NMP), the glycosylation proceeded to afford the desired glycoside **6** (Table 2; entries 4–12). The reaction at 250 °C in DMF provided glycoside **6** in excellent yield and high α selectivity (Table 2; entries 10 and 11). Similarly, the reactions conducted at 200 °C in DMF gave higher yields for both **3a** and **3b** donors within 1 h. (Table 2; entry 8 and 9). However, reactions conducted at 150 °C required somewhat longer reaction time-courses for product formation (Table 2; entries 6 and 7). Reactions in which the temperature was noted to be below 150 °C yielded no product, as observed by NMR. Lower yields were observed in glycosylation reactions with NMP when comparing to DMF (Table 2; entry 12). In all cases, DMF as the solvent proved to produce the best results using our reaction conditions.

As noted in the literature, DMF is frequently used as a polar aprotic solvent for various transformations and can be degraded into many intermediates such as HCO_2_H, CO, HNMe_2_, HCONMe_2_, NH_2_COH, etc. [21]. To answer the question as to why DMF might be promoting efficient glycosylation at a high temperature under microwave irradiation, we performed a control experiment employing DCM in the presence of *N*,*N*-dimethylamine (10 eq with respect to donor). When compound **3a** and **3b** were subjected to microwave irradiation, product **6** was obtained. The reaction did require a longer reaction time for completion, most likely because the maximum microwave heating temperature for DCM is 150 °C [18]. When combined, the results confirm that the involvement of the *N*,*N*-dimethylamine can promote glycosylation; *N*,*N*-dimethylamine might be generated in situ from decomposition of DMF under our described reaction conditions [22]. The use of *N*,*N*-dimethylamine in DCM did not affect the yield nor the α/β ratio. Both **3a** and **3b** donors gave similar results with respect to yield and in all cases resulted in higher α selectivity. To analyze the role of dimethylamine as a base, we tested DMAP and triethylamine under the same conditions. However, no product formation was observed. This clearly indicates that dimethylamine does not only play a role as a base. The power (300 W) and pressure (250 psi) of our instrument were kept constant for all comparisons.

**Figure 3:**
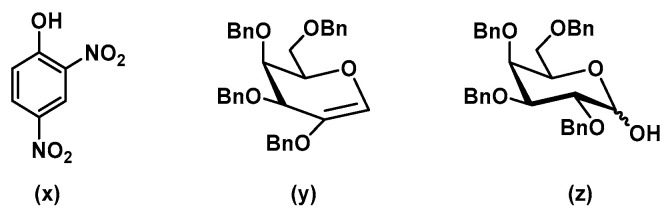
By-products of glycosylation when using donor **3**.

The reaction between donor **3** and 6-OH acceptor **5** at 250 °C in DMF gave a desired disaccharide **6** with an excellent yield of 80% (Table 2; entry 10). This is explained by the electron-withdrawing nitro group, which makes 2,4-DNP an excellent leaving group by stabilizing its conjugate base (compound (**x**), Figure 3).

Having established that DMF could serve as the best solvent under our microwave conditions, we elected to utilize 0.03 M–0.04 M for acceptor concentration and 0.07 M–0.08 M donor concentration. This concentration ratio was based off our maximum yields obtained. In our studies, we noted that use of excess donor (1.5 eq. of donor and 1 eq. of acceptor) provided the best overall yields. However, use of excess acceptor reduced the number of by-products related to the donor (elimination product and hydrolysis product; (**y**) and (**z**) respectively). 2,4-dinitrophenol (**x**) was isolated as the sole by-product, which is derived from the donor. In addition, 2,3,4,6-tetra-*O*-benzyl-d-galactal (**y**), the elimination product (Figure 3), was formed when temperatures reached more than 150 °C. Furthermore, tetra-*O*-benzyl-d-galactopyranose (**z**), the hydrolysis product, could be formed as a result of H_2_O being added to the reaction mixture [9].

Using entries 8 and 9 (Table 2) as our optimized condition for the glycosylation, we turned our attention to increasing the substrate scope of the reaction by screening a range of acceptors (Table 3). We elected to use a number of sugar acceptors and examined the reaction of **3a** and **3b** with 6-OH acceptor **7**. This is a primary acceptor which carries acid-sensitive isopropylidene groups, which proved to be a compatible substrate for this glycosylation protocol and afforded **13** in 60–68% (depending on α- or β-donor) with overall high *α* selectivity (Table 3; entries 1 and 2). Further experiments of monosaccharide acceptors showed that secondary hydroxyl groups at positions 2 or 3 also afforded good to moderate yields. Secondary 2-OH acceptor **8** produced disaccharide **14** in a 65% yield from **3a** with high *α*-selectivity (Table 3; entry 3) and a 58% yield from **3b** with 3:1 *α*/*β* ratio (Table 3; entry 4). When **3a** and **3b** were reacted with 3-OH acceptor **9**, disaccharide **15** was obtained in 62–65% with high *α*-selectivity (Table 3, entry 5 and 6). Unsurprisingly, the 4-OH acceptor **10** is comparatively less reactive to other secondary acceptors and it was completely inert under our reaction conditions. It was gratifying to observe, however, that the reaction of 1-adamantanol **11**, a tertiary alcohol, gave the resultant product **17** in 65–70% with exclusive *α*-selectivity (Table 3; entry 9 and 10). Cholesterol **12**, as a secondary acceptor, gave high *α*-selectivity as well (Table 3; entry 11 and 12) from both **3a** and **3b** donors.

Based on our successful attempts with 2,4-DNP galactosyl donor **3a** and **3b**, we sought to investigate the synthesis of a trisaccharide under our microwave conditions (Table 4). To accomplish this, we synthesized disaccharide 2,4-DNP donor **19** using the double-base method [6], and then we examined the reactions of disaccharide 2,4-DNP donor **19** and 6-OH D-galactosyl acceptor **5**. Trisaccharide **20** was obtained in a modest yield of 40% and 35% from **19a** and **19b** disaccharide donors, respectively (Table 4; entries 1 and 2) [23].

Although DMF is not very common in glycosylation, it has been utilized in glycosylation reactions as a solvent and as an additive to effect *α*-glycosylation by the Mong group, as noted in the literature [24,25]. In the course of our studies, we obtained 1,2-*cis* (*α*) as the major product. We believe this is the result of the addition of DMF to the oxocarbenium intermediate, forming both *α* and *β* glycosylimidates [25]. In addition to the modulating properties, DMF is a good microwave-absorbing solvent which may promote the thermal activation of glycosyl donors.

## 3. Conclusions

In conclusion, this microwave-assisted glycosylation method provides an important platform for the formation of glycosides under operationally simple conditions relative to the conventional glycosylation methods. Herein, we have introduced a new donor with a microwave-labile leaving group 2,4-DNP. Benzyl-protected (2,4-DNP donor) **3a** and **3b** donors showed a similar reactivity and inversion of configuration was observed under solvolysis conditions. *O*-glycosylations with the 2,4-DNP donor and various acceptors in DMF were promoted by microwave heating at elevated temperatures. Furthermore, we were able to synthesize a trisaccharide using optimized microwave reaction conditions. We believe that there is an effect of *N*,*N*-dimethylamine (base), which might form in situ during the reaction. Further investigations of compatibility of electron-withdrawing (disarming) acetate and benzoyl-protecting groups and the role of the *N*,*N*-dimethylamine are currently underway in our lab and will be reported in due course.

## Figures and Tables

**Table 1:** Solvolysis of 2,4-DNP donor.

* 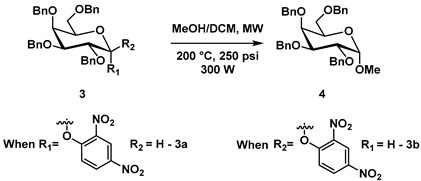 *					
**Entry**	**Donor**	**Solvent**	**Time (min)**	**Yield % ^a,f^**	***α*/*β* ^b^**
1	3a	MeOH/DCM ^c^	30	84	3:1
2	3b	MeOH/DCM ^c^	30	87	α only
3	3a/b ^d^	MeOH/DCM ^c^	30	81	1.3:1
4	3a/b ^e^	MeOH/DCM ^c^	30	N.R.	n/a

^a^ Isolated yields. ^b^ *α*/*β* ratios were measured using the anomeric peak in the ^1^H NMR of the unpurified material. ^c^ MeOH/DCM 9:1, (1 mL). ^d^ A 1:1 mixture of **3a** and **3b** was used, 100 mg scale, 0.14 mmol. ^e^ A 1:1 mixture of **3a** and **3b** was used under refluxing conditions. ^f^ Combined yield of both α and β products. N.R.—no reaction. n/a—not available.

**Table 2:** Preliminary reaction optimization under microwave conditions. ^a^

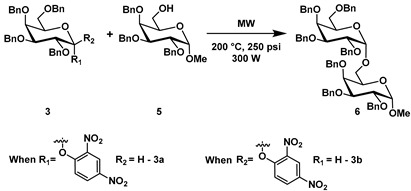						
**Entry**	**Donor ^a^**	**Solvent**	**T °C**	**Time (h)**	**α/β ^e^**	**Yield % ^b,d^**
1	3a/b	DCM	150	2	-	N.R.
2	3a/b	THF	200	2	-	negligable
3	3a/b	ACN	200	2	-	N.R.
4	3a	DMF	100	2	-	N.R.
5	3b	DMF	100	2	-	N.R.
6	3a	DMF	150	2	3:1	71
7	3b	DMF	150	2	3:1	80
8	3a	DMF	200	1	2.9:1	72
9	3b	DMF	200	1	3:1	80
10	3a	DMF	250	1	3:1	80
11	3b	DMF	250	1	3:1	86
12	3a/b	NMP	250	1	2.5:1	30
13	3a	DCM/Dimethylamine ^c^	150	2	3:1	65
14	3b	DCM/Dimethylamine ^c^	150	2	3:1	70
15	3a/b	DCM/Triethylamine ^c^	150	2	-	N.R.
16	3a/b	DCM/DMAP ^c^	150	2	-	N.R.

^a^ Reactions were conducted with 1.2 eq. of **3a**/**b** 100 mg scale, 0.14 mmol, and 1 eq. of acceptor **5** in 2 mL of solvent. ^b^ Isolated yields. ^c^ A 10 eq. of dimethylamine. N.R.—no reaction. ^d^ Combined yield of both α and β products, ^e^ *α*/*β* ratios were measured using anomeric peak of the ^1^H NMR of the unpurified material.

**Table 3:** Glycosylation of different acceptors with per-benzylated galactosyl DNP donor. ^a^

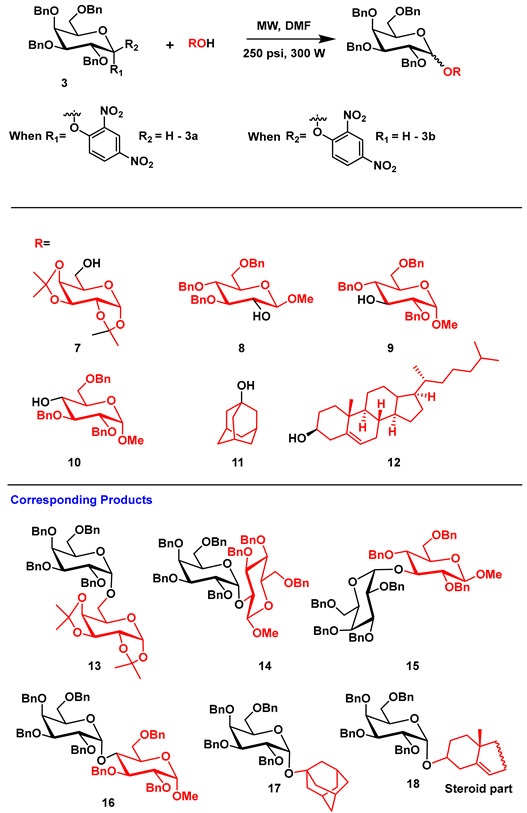							
**Entry**	**Donor** **1.2 eq**	**Acceptor** **1 eq**	**Temp** **°C**	**Time** **h**	**Product**	**Yield ^b,d^** **%**	***α*/*β* ^c^**
1	3a	7	200	1	13	68	3:1
2	3b	7	200	1	13	60	2.1:1
3	3a	8	200	1	14	65	α only
4	3b	8	200	1	14	58	3:1
5	3a	9	200	1	15	62	3.5:1
6	3b	9	200	1	15	65	2:1
7	3a	10	200	1	16	n.r.	n/a
8	3b	10	200	1	16	n.r.	n/a
9	3a	11	200	1	17	65	*α* only
10	3b	11	200	1	17	70	*α* only
11	3a	12	200	1	18	80	*α* only
12	3b	12	200	1	18	75	*α* only

^a^ Reactions were conducted with 1.2 eq. of **3a/b** (100 mg scale, 0.14 mmol) and 1 eq. of acceptor in 2 mL of DMF. ^b^ Isolated yields. ^c^ *α*/*β* ratios were measured using anomeric peak of the ^1^H NMR of the unpurified material. ^d^ Combined yield of both α and β products.

**Table 4:** Synthesis of trisaccharide with DNP glycoside as a donor.

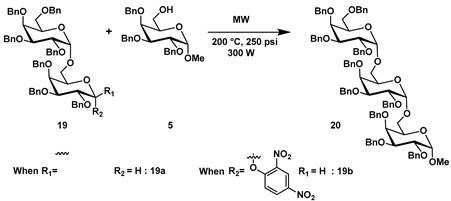							
**Entry**	**Donor**	**Acceptor**	**Temp (°C)**	**Time**	**Product**	**Yield ^b,d^ (%)**	***α*/*β* ^c^**
1	19a	5 ^a^	200	1.5	20	40	*α* only
2	19b	5 ^a^	200	1.5	20	35	*α* only

^a^ Reactions were conducted with 1.2 eq. (100 mg scale, 0.09 mmol) of **19a/b** and 1 eq. of acceptor **5** in 2 mL of DMF. ^b^ Isolated yields. ^c^ *α*/*β* ratios were measured using anomeric peak of the ^1^H NMR of the unpurified material. ^d^ Combined yield of both α and β products.

## Data Availability

The original contributions presented in this study are included in this article/Supplementary Materials. Further inquiries can be directed to the corresponding author.

## Supplementary Materials

The following supporting information can be downloaded at: https://www.mdpi.com/article/10.3390/molecules30183693/s1 [6,26,27,28,29,30,31,32,33,34].
