# Supplementary material for: Microwave-Assisted Neutral Glycosylation Reactions in the Absence of Reagent Activators
Source: Molecules. 2025 Sep 11;30(18):3693. doi: 10.3390/molecules30183693 (PMC12472276; doi:10.3390/molecules30183693)

Supporting Information

Microwave-Assisted Neutral Glycosylation Reactions in the Absence of Reagent Activators

Shanika M. P. Gamage [1], Geraud Valentin [1], Samir Ghosh [1], Pradheep Eradi [1], Rahul S. Bagul [2], David Crich [2] and Peter R. Andreana [1,*]

1 Department of Chemistry and Biochemistry, University of Toledo, Toledo, OH 43606, USA

2 Department of Chemistry and College of Pharmacy, University of Georgia, Athens, GA 30602, USA

*Correspondence: peter.andreana@utoledo.edu; Tel.: +1-419-530-1930

Table of Content	
**Heading**	**Page No**
**General Information Experiments, Equipment and Materials**	**SI2**
**Abbreviation**	**SI2**
**Experimental Procedures**	**SI3**
**Synthesis of microwave labile donors**	**SI3-SI24**
**Trisaccharide synthesis**	**SI25-Sl29**
**Reaction conditions and results of the microwave reactions**	**SI30-SI32**
**Spectroscopic data**	**SI33-SI83**

General Information Experiments, Equipment and Materials

All commercial chemicals/reagents were obtained without further purification unless specialized and were obtained from commercial sources (Sigma, Oakwood, Alfa and AK scientific). All reactions were carried out in dry solvents, oven dried glassware and under inert atmosphere (Argon) unless specialized. Thin layer chromatography was performed using Silicycle silica gel plates (0.25 thickness), visualized under UV light, and stained with p- anisaldehyde staining solution.

Proton nuclear magnetic resonance ^{1}H NMR, and proton-decoupled carbon nuclear magnetic resonance ^{13}C NMR experiments were performed using Bruker Avance 600 MHz spectrometer at the University of Toledo nuclear magnetic resonance facility. The solvent peak was referenced to 7.26 ppm for 1H and 77.16 ppm for ^{13}C for $CDCl_3$. The NMR data are represented as follows, Chemical Shift in ppm, integration, multiplicity (s-singlet, d-doublet, t-triplet, q-quartet, m-multiplet) and coupling constants in Hertz.

Electron spray ionization (ESI) and ion trap analyzer techniques were used for low resolution mass. Electron spray ionization (ESI) and time of flight (TOF) techniques were used to obtain high resolution mass spectra.

All the microwave reactions were performed in Microwave Synthesizer Discover SP. It is a single mode reactor with the following features, performs reactions temperature up to 300 °C, Patented infrared temperature sensor, integrated stirring and patented powerMAX technology.

Abbreviation

THF: Tetrahydrofuran, DMF: N, N-Dimethylformamide, EtOAc: Ethyl acetate, DCM: Dichloromethane, NMP: N-methyl-2- pyrrolidone, $NaHCO_3$: Sodium bicarbonate, Na_2SO_4: Sodium sulfate, ESI: Electrospray ionization, rt: Room temperature, 2,4 DNP: 2,4 Dinitrophenol, DMA: Dimethylamine

Experimental Procedures

Synthesis of microwave labile donors

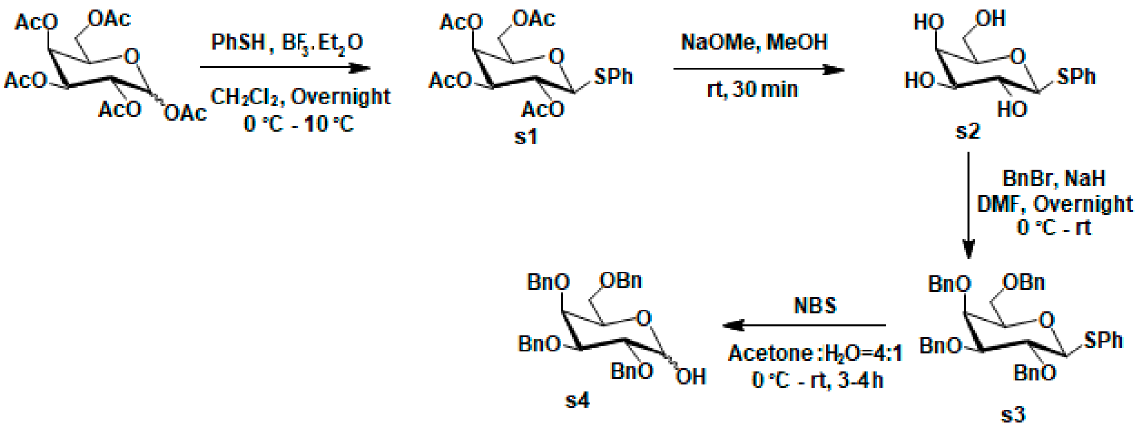

Compounds s1, s2, s3, and s4

Comparison of our data of compounds **s1**, **s2**, **s3** and **s4** with the literature report [26] which is nicely revealed identity with respect to ^{1}H and ^{13}C spectra.

2,3,4,6-tetra-*O*-benzyl-D-galactopyranosyl phenylacetate (1)

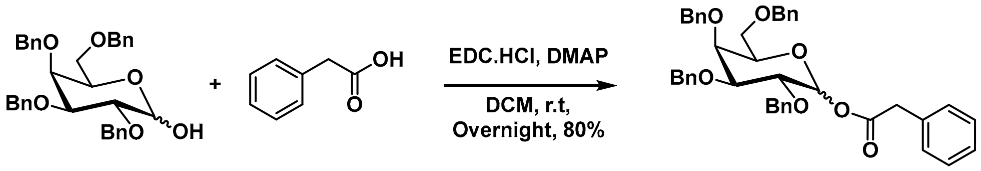

To solution of **s4** (1.00 g, 1.85 mmol) in dry CH_2Cl_2 (10 mL), phenyl acetic acid (0.50 g,3.70 mmol), and DMAP (0.220 g, 1.85 mmol) were added and then the mixture was cooled to 0 ℃. EDC.HCl (0.750 g, 3.88 mmol) was added portion wise to the mixture at 0 ℃. The reaction mixture was allowed to warm to room temperature and stirred for 24 h. After TLC indicated the complete consumption of the starting material, the reaction mixture was washed with water (3 × 10 mL),

followed by saturated NaCl (10 mL) solution, and then dried over anhydrous Na_2SO_4. The crude product was purified by flash column chromatography using ethyl acetate and hexane as eluents to afford phenyl acetate donor 1 as an *α*/β (3:1) mixture in 80% yield (0.97 g of isolated amount).

Only *α* anomer was reacted under microwave conditions to afford glycosylated product.

2,3,4,6-tetra-*O*-benzyl- *α*-D-galactopyranosyl phenylacetate (1*α*)

Rf- 0.76 (hexane/EtOAc, 2:1)

<u>^{1}H NMR (600 MHz, $CDCl_3$)</u> :- δ 7.41 – 7.25 (m, 25H, Ar-H), 6.45 (d, *J* = 3.5 Hz, 1H,H^1), 4.97 (d, *J* = 11.4 Hz, 1H, PhC*H*$_2$), 4.80 (d, *J* = 11.9 Hz, 1H, PhC*H*$_2$), 4.73 – 4.68 (m, 3H, PhC*H*$_2$), 4.60 (d, *J* = 11.4 Hz, 1H, PhC*H*$_2$), 4.46 (d, *J* = 11.7 Hz, 1H, PhC*H*$_2$), 4.40 (d, *J* = 11.7 Hz, 1H, PhC*H*$_2$), 4.18 (dd, *J* = 10.1, 3.7 Hz, 1H, H^2), 3.99 (dd, *J* = 3.1, 1.0 Hz, 1H, H^4), 3.90 – 3.87 (m, 1H, H^5), 3.78 (dd, *J* = 10.0, 2.8 Hz, 1H, H^3), 3.71 (s, 2H, CH_2), 3.55 (dd, *J* = 9.3, 7.4 Hz, 1H, H^6), 3.49 (dd, *J* = 9.2, 5.7 Hz, 1H, H^6).

<u>^{13}C NMR (151 MHz, $CDCl_3$)</u>:- δ 170.1 (C=O), 138.7(Ar-C), 127.1 (Ar-C), 91.3 (C^1), 78.5 (C^3), 75.6 (C^2), 74.9 (Ph*C*H$_2$), 74.6 (C^4), 73.6 (Ph*C*H$_2$), 73.4 (Ph*C*H$_2$), 73.2 (Ph*C*H$_2$), 72.0 (C^5), 68.5 (C^6), 41.6 (CH_2).

OR: $[\alpha]_D^{22.5}$ = +118.0 (C = 0.541 g/100 mL in $CHCl_3$)

ESI-MS calcd. for $C_{42}H_{42}O_7^+$ $[M+K]^+$: 697.79, found 697.74.

<u>General method for preparation of glycosyl donor</u>

2-methoxycarbonyl-4-nitrophenyl 2,3,4,6-tetra-O-benzyl-D-galactopyranoside (2)

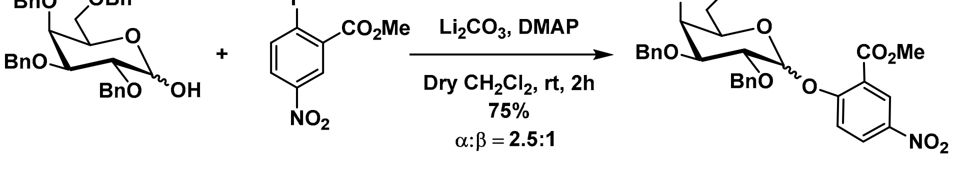

The titled compound **2** was synthesized according to the general glycosyl donor synthesis. Compound **s4** (1.00 g, 0.960 mmol, 1 eq), Li_2CO_3 (0.140 g, 1.85 mmol, 2 eq.), and 2,4-Methyl 2-fluoro-5-nitrobenzenecarboxylate (0.221 g, 1.11 mmol, 1.2 eq.) were dissolved in dry CH_2Cl_2 (10 mL) in a dried flask and the reaction mixture was stirred at room temperature. DMAP (0.112 g, 0.930 mmol, 1 eq.) was dissolved in CH_2Cl_2 and added to the suspension over 20 minutes in 5 portions. Upon addition of DMAP, the suspension changed color to a strong yellow brown to dark red. The reaction was allowed to stir at room temperature for 1–2 hours. After TLC indicated complete consumption of the starting materials, the product was purified by silica gel chromatography using a hexane: ethyl acetate gradient. The desired compound was obtained as a yellow oil in 70% yield, with a 50% α-anomeric ratio (isolated amount of 0.93 g).

2-methoxycarbonyl-4-nitrophenyl 2,3,4,6-tetra-O-benzyl-α-D-galactopyranoside (2a)

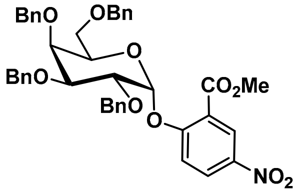

Rf- 0.67 (hexane/EtOAc, 2:1)

<u>^{1}H NMR (600 MHz, $CDCl_3$)</u>:- δ 8.70 (d, *J* = 2.9 Hz, 1H, Ar-H), 8.22 (dd, *J* = 9.4, 2.9 Hz, 1H, Ar-H), 7.47 – 7.44 (m, 2H, Ar-H), 7.41 – 7.36 (m, 2H, Ar-H), 7.36 – 7.22 (m, 10H, Ar-H), 7.18 – 7.10 (m, 4H, Ar-H), 5.62 (d, *J* = 2.9 Hz, 3H, H^1), 5.00 (d, *J* = 11.2 Hz, 1H, PhC$\underline{H_2}$), 4.93 (d, *J* = 7.0 Hz, 1H, PhC$\underline{H_2}$), 4.91 (d, *J* = 6.8 Hz, 1H, PhC$\underline{H_2}$), 4.85 (d, *J* = 11.9 Hz, 1H, PhC$\underline{H_2}$), 4.68 (d, *J* = 12.1 Hz, 1H, PhC$\underline{H_2}$), 4.61 (d, *J* = 11.4 Hz, 1H, PhC$\underline{H_2}$), 4.33 (dd, *J* = 28.5, 11.6 Hz, 2H, PhC$\underline{H_2}$), 4.27 (m, H^2 and H^3), 4.08 – 4.04 (m, 2H, H^4 and H^5), 3.82 (s, 3H, OCH_3), 3.48 (ddd, *J* = 33.7, 9.8, 6.7 Hz, 2H, H^6).

<u>^{13}C NMR (151 MHz, $CDCl_3$)</u>:- δ 164.5 (C=O), 160.7, 141.5, 138.6-115.6 (Ar-C), 96.7 (C^1), 78.6(C^3), 76.1(C^2), 75.0(C^5), 74.8 (C^6), 73.9 (Ph$\underline{C}H_2$), 73.4 (Ph$\underline{C}H_2$), 73.3 (Ph$\underline{C}H_2$), 71.5 (Ph$\underline{C}H_2$), 68.7(Ph$\underline{C}H_2$), 52.6 (OCH_3).

HRMS calcd. for $C_{42}H_{43}NO_{11}^+$ $[M+H_2O]^+$: 737.2836, found 737.3071.

OR: $[\alpha]_D$ $^{22.5}$ = +67.6 (C =0.630 g/100mL in $CHCl_3$)

2,4-Dinitrophenyl 2,3,4,6-tetra-*O*-benzyl-D-galactopyranoside (3)

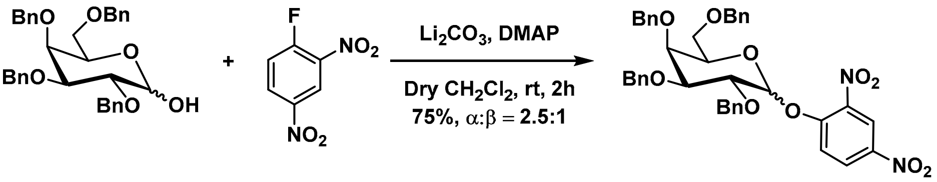

Compound **s4** (1.00 g, 1.85 mmol, 1 eq), Li_2CO_3 (0.270 g, 3.70 mmol, 2 eq.), and Dinitrofluorobenzene (0.413 g, 2.22 mmol, 1.2 eq.) were dissolved in dry CH_2Cl_2 (10 mL) in a dried flask and the reaction mixture was stirred at room temperature. DMAP (0.225 g, 1.85 mmol, 1 eq.) was dissolved in CH_2Cl_2 and added to the suspension over 20 minutes in 5 portions. Upon addition of DMAP, the suspension changed color to a strong yellow brown to dark red. The reaction was allowed to stir at room temperature for 1–2 hours. After TLC indicated complete consumption of the starting materials, the product was purified by silica gel chromatography using a hexane: ethyl acetate gradient. The product was obtained as a yellow oil in 75% yield. The 0.71 g of **α**- and 0.27 g of **β**-anomers were isolated in 54% and 21% yield, respectively.

2,4-Dinitrophenyl 2,3,4,6-tetra-*O*-benzyl-*α*-D-galactopyranoside (3a)

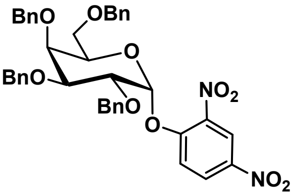

Rf = 0.57 (hexane/EtOAc, 4:1) and

^{1}H NMR (600 MHz, $CDCl_3$):- δ 8.73 (d, *J* = 2.7 Hz, 1H, Ar-H), 8.23 (dd, *J* = 9.2, 2.7 Hz, 1H, Ar-H), 7.46 – 7.11 (m, 21H, Ar-H), 5.59 (d, *J* = 3.5 Hz, 1H,H^1), 4.98 (d, *J* = 11.2 Hz, 1H, PhC$\underline{H}_2$), 4.90 (dd, *J* = 11.9, 2.1 Hz, 2H, PhC$\underline{H}_2$), 4.81 (d, *J* = 11.6 Hz, 1H, PhC$\underline{H}_2$), 4.60 (dd, *J* = 21.5, 11.7 Hz, 2H, PhC$\underline{H}_2$),

4.33 (q, *J* = 11.7 Hz, 2H, PhC*H*2), 4.24 (dd, *J* = 10.1, 3.3 Hz, 1H,H^2), 4.17 (dd, *J* = 9.9, 2.7 Hz, 1H,H^3) 3.99 (m, 2H, H^4 and H^5), 3.46 (d, *J* = 6.4 Hz, 2H H^6).

^{13}C NMR (151 MHz, $CDCl_3$):- δ 154.7 (Ar-C), 140.9 - 137.7 (Ar-C), 128.8 - 127.7 (Ar-C), 121.8 (Ar-C), 117.2 (Ar-C), 98.1 (C^1), 78.4 (C^3), 77.4 (C^2), 75.1 (C^5), 74.9 (Ph*C*H2), 74.1 (Ph*C*H2), 73.8 (Ph*C*H2), 73.5 (Ph*C*H2), 72.2 (C^4), 68.7 (C^6).

OR: $[\alpha]_D^{22.5}$ = +59.2 (C =0.24 g/100 mL in $CHCl_3$)

ESI-MS calcd. for $C_{40}H_{38}N_2O_{10}^+$ $[M+Na]^+$: 729.25, found 729.24.

2,4-Dinitrophenyl 2,3,4,6-tetra-*O*-benzyl-*β*-D-galactopyranoside (3b)

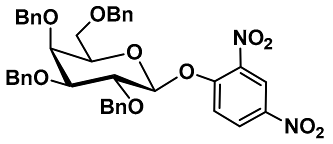

Rf = 0.22 (hexane/EtOAc, 4:1)

^{1}H NMR (600 MHz, $CDCl_3$):- δ 8.73 (d, *J* = 2.7 Hz, 1H, Ar-H), 8.19 (dd, *J* = 9.3, 2.8 Hz, 1H, Ar-H), 7.42 – 7.19 (m, 21H, Ar-H), 5.16 (d, *J* = 7.7 Hz, 1H,H^1), 4.98 (d, *J* = 10.8 Hz, 2H, PhC*H*2), 4.87 (d, *J* = 10.3 Hz, 1H, PhC*H*2), 4.80 (q, *J* = 11.7, 25.1 Hz 2H, PhC*H*2), 4.64 (d, *J* = 11.6 Hz, 1H, PhC*H*2), 4.42 (d, *J* = 2.3 Hz, 2H, PhC*H*2), 4.22 (dd, *J* = 9.8, 7.5 Hz, 1H,H^2), 3.92 (d, *J* = 2.9 Hz, 1H,H^4), 3.75 (t, *J*=6.13 Hz, 1H, H^5), 3.69 – 3.59 (m, 2H,H^3 and H^6), 3.50 (dd, *J* = 9.6, 5.2 Hz, 1H, H^6).

^{13}C NMR (151 MHz, $CDCl_3$):- δ 154.6 (Ar-C), 141.2 - 137.7 (Ar-C), 129.3 - 127.7 (Ar-C), 122.1 (Ar-C), 121.7 (Ar-C), 117.6 (Ar-C), 113.7 (Ar-C), 101.4 (C^1), 81.9 (C^3), 78.9 (C^2), 75.7 (C^5), 75.1 (Ph*C*H2), 74.78 (Ph*C*H2), 73.9 (Ph*C*H2), 73.4 (Ph*C*H2), 72.9 (C^4), 69.2 (C^6).

OR: $[\alpha]_D^{22.5}$ = -46.7 (C = 0.00042 in $CHCl_3$)

ESI-MS calcd. for $C_{40}H_{38}N_2O_{10}^+$ $[M+Na]^+$: 729.25, found 729.24.

Preparation of glycosyl acceptors

Methyl 2,3,4-tri-*O*-benzyl-*α*-D-galactopyranoside (5)

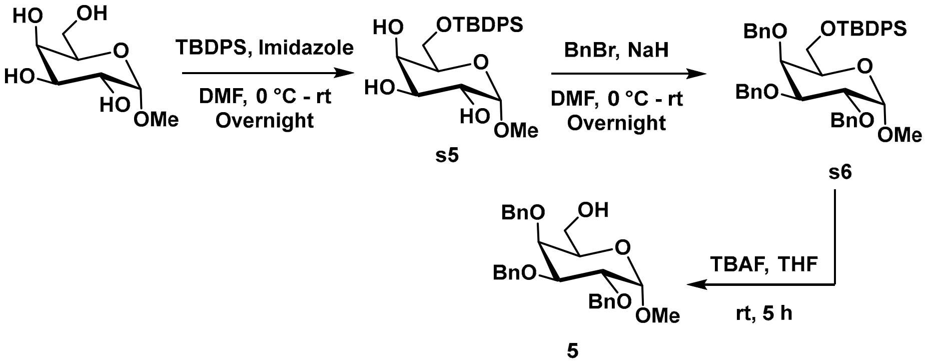

Comparison of our data of compounds **s5**, **s6,** and **5** with the literature report revealed identity with respect to ^{1}H and ^{13}C spectra [27,28].

Rf = 0.24 (hexane/EtOAc, 2:1)

^{1}<u>H NMR (600 MHz, CDCl$_3$)</u>:- δ 7.38 – 7.18 (m, 15H, Ar-H), 4.92 (d, *J* = 11.6 Hz, 1H, PhC<u>*H*</u>$_2$), 4.84 (d, *J* = 11.7 Hz, 1H, PhC<u>*H*</u>$_2$), 4.79 (d, *J* = 11.9 Hz, 1H, PhC<u>*H*</u>$_2$), 4.70 (d, *J* = 11.9 Hz, 1H, PhC<u>*H*</u>$_2$), 4.66 (d, J=3.83, 1H, H^1), 4.64 (d, *J* = 8.5 Hz, 1H, PhC<u>*H*</u>$_2$), 4.59 (d, *J* = 11.6 Hz, 1H, PhC<u>*H*</u>$_2$), 4.00 (dd, *J* = 10.1, 3.6 Hz, 1H,H^2), 3.89 (dd, *J* = 10.1, 2.9 Hz, 1H,H^3), 3.82 (d, *J* = 3.1 Hz, 1H, H^4), 3.70 – 3.62 (m, 2H, H^5, H^6), 3.42 (dd, *J* = 9.7, 3.6 Hz, 1H, H^6), 3.31 (s, 3H, OCH_3).

^{13}<u>C NMR (151 MHz, CDCl$_3$)</u>:- δ 138.8 - 127.6 (Ar-C), 98.9 (C^1), 79.2 (C^3), 76.5 (C^2), 75.1 (C^4), 74.5 (Ph<u>C</u>*H*$_2$), 73.7, (Ph<u>C</u>*H*$_2$) 70.3 (C^5), 62.4 (C^6), 55.4 (OCH_3).

ESI-MS calcd. for $C_{28}H_{32}N_2O_6^+$ [M+Na]$^+$: 487.56, found 487.22.

OR: $[\alpha]_D^{22.5}$ = +110.0 (C = 1 mg/1 mL in $CHCl_3$)

Methyl 3,4,6-tri-*O*-benzyl-*β*-D-glucopyranoside (8)

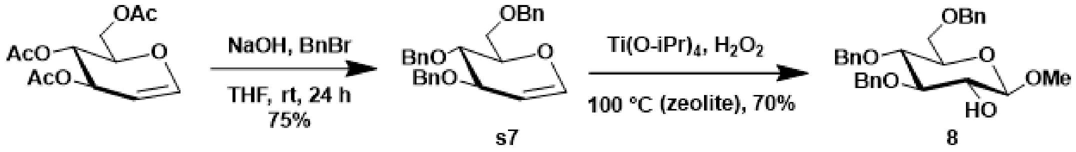

Benzylation of 3, 4, 6- tri-O-acetyl glucal afforded compound **s7** as a pale yellow oil and **s7** was subjected to Epoxidation-Alcoholysis using Ti catalyst [29]. Analytical data for **s7** and **8** were in accordance with those reported previously [29].

Rf = 0.3 (hexane/EtOAc, 2:1)

^{1}H NMR (600 MHz, $CDCl_3$):- δ 7.40 – 7.13 (m, 15H), 4.91 (d, *J* = 11.3 Hz, 1H, PhC*H*$_2$), 4.86 (d, *J* = 11.3 Hz, 1H, PhC*H*$_2$), 4.83 (d, *J* = 10.8 Hz, 1H, PhC*H*$_2$), 4.63 (d, *J* = 12.2 Hz, 1H, PhC*H*$_2$), 4.56 (d, *J* = 8.8 Hz, 1H, PhC*H*$_2$), 4.54 (d, *J* = 7.4 Hz, 1H, PhC*H*$_2$), 4.19 (d, *J* = 7.6 Hz, 1H,H^1), 3.76 (dd, *J* = 10.8, 2.0 Hz, 1H,H^6), 3.71 (dd, *J* = 10.8, 4.6 Hz, 1H,H^6), 3.59 (t, *J* = 8.8 Hz, 1H), 3.66-3.59 (m, 2H, H^3, H^4), 3.57 (s, 3H,OCH_3), 3.56-3.50 (m, 2H, H^2, H^5).

^{13}C NMR (151 MHz, $CDCl_3$):- δ 138.7 - 138.2 (Ar-C), 128.6 - 127.8 (Ar-C), 103.8 (C^1), 84.6, 76.9, 75.3, 75.2, 74.7 (Ph*C*H$_2$), 73.6 (Ph*C*H$_2$), 68.9 (Ph*C*H$_2$), 57.3 (OCH_3).

ESI-MS calcd. for $C_{28}H_{32}O_6^+$ [M+Na]$^+$: 487.56, found 487.22.

OR: $[\alpha]_D^{22.5}$ = -15.0 (C = 1 mg/1 mL in $CHCl_3$)

Methyl 2,4,6-tri-*O*-benzyl-*β*-D-glucopyranoside (9)

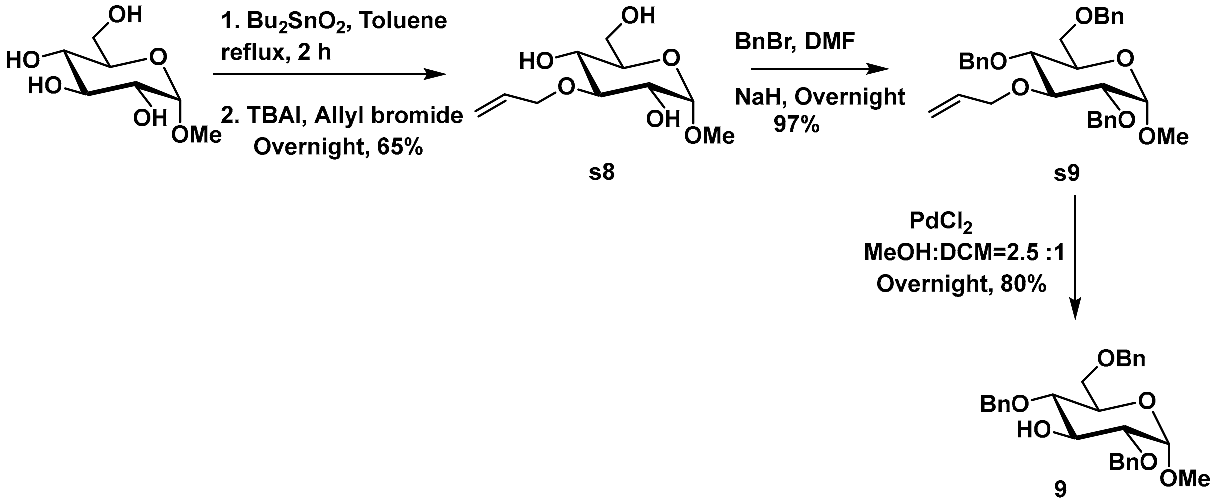

Comparison of our data of compounds **s8**, **s9** and **9** with the literature report revealed identity with respect to ^{1}H and ^{13}C spectra[4].

Rf = 0.5 (hexane/EtOAc, 2:1)

<u>^{1}H NMR (600 MHz, $CDCl_3$)</u> δ 7.40 – 7.25 (m, 13H), 7.16 (dd, J = 7.6, 2.0 Hz, 2H), 4.91 (d, J = 12.0 Hz, 1H), 4.88 (dd, J = 27.9, 11.2 Hz, 1H), 4.82 (d, J = 2.2 Hz, 1H), 4.64 (d, J = 12.3 Hz, 1H), 4.52 (t, J = 11.2 Hz, 2H), 3.79 – 3.71 (m, 3H), 3.67 (ddd, J = 28.1, 10.9, 5.4 Hz, 2H), 3.43 (s, 3H).

<u>^{13}C NMR: (150 MHz, $CDCl_3$)</u> δ 138.8 - 138.0 (Ar-C), 128.6 - 127.8 (Ar-C), 99.5 (C^1), 83.3, 77.5, 75.5, 75.1, 73.6, (C_2, C_3, C_4, C_5, Ph<u>C</u>*H_2*), 68.5 (C^6), 55.3 (OCH_3).

ESI-MS calcd. for $C_{28}H_{32}O_6^+$ [M+Na]$^+$: 487.22, found 487.57.

OR: $[\alpha]_D^{22.5}$ = +100.0 (C = 1 mg/ 1 mL in $CHCl_3$)

Methyl 2,3,6-tri-*O*-benzyl-*β*-D-glucopyranoside (10)

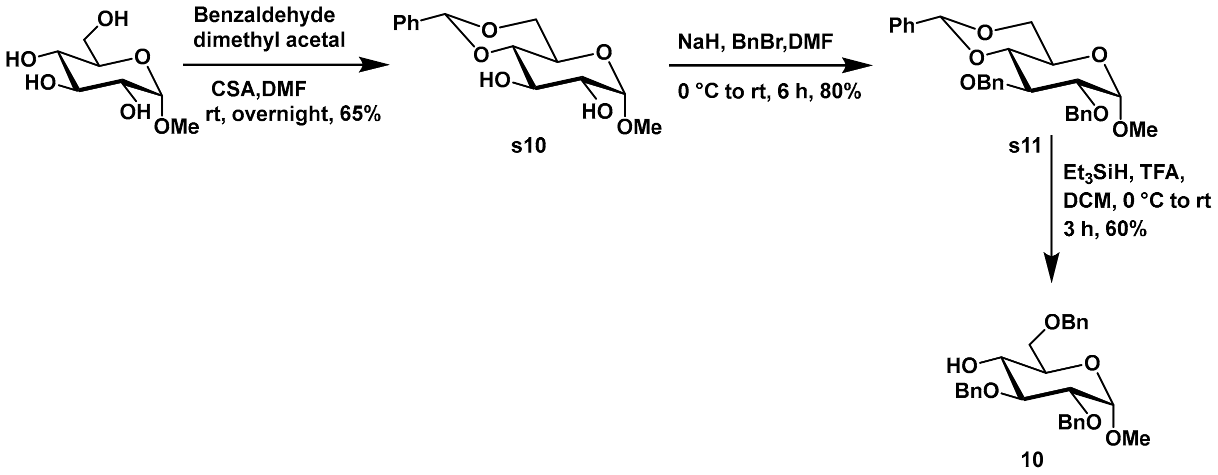

Comparison of our data of compounds **s10**, **s11** and **10** with the literature report revealed identity with respect to ^{1}H and ^{13}C spectra.[4]

Rf = 0.38 (hexane/EtOAc, 2:1).

^{1}H NMR (600 MHz, $CDCl_3$):- δ 7.33 – 7.19 (m, 15H, Ar-H), 4.95 (d, *J* = 11.4 Hz, 1H, PhC*H*$_2$), 4.71 (d, *J* = 12.1 Hz, 1H, PhC*H*$_2$), 4.68 (d, *J* = 11.4 Hz, 1H, PhC*H*$_2$), 4.60 (d, *J* = 12.1 Hz, 1H, PhC*H*$_2$), 4.57 (d, *J* = 3.5 Hz, 1H, H^1), 4.51 (dd, *J* = 29.0, 12.3 Hz, 2H, PhC*H*$_2$), 3.73 (t, *J* = 9.2 Hz, 1H, H^3), 3.65 (dd, *J* = 9.6, 3.8 Hz, 1H, H^5), 3.62 (d, *J* = 3.8 Hz, 1H, H^6), 3.55 (t, *J* = 9.3 Hz, 1H, H^4), 3.48 (dd, *J* = 9.5, 3.7 Hz, 1H, H^2), 3.33 (s, 3H, OCH_3).

^{13}C NMR (151 MHz, $CDCl_3$):- δ 138.8-127.7 (Ar-C), 98.2 (C^1), 81.5 (C^3), 79.6 (C^2), 75.5 (Ph<u>C</u>*H*$_2$), 73.6 (Ph<u>C</u>*H*$_2$), 73.2 (Ph<u>C</u>*H*$_2$), 70.7 (C^4), 69.9 (C^5), 69.5 (C^6), 55.3 (OCH_3).

ESI-MS calcd. for $C_{28}H_{32}O_6^+$ [M+Na]$^+$: 487.22, found 487.67

OR: $[\alpha]_D^{22.5}$ = +11.0 (C = 1 mg/ 1 mL in $CHCl_3$)

Microwave assisted glycosylation

General method for microwave assisted glycosylation

The glycosyl donor (1.2 eq), and acceptor (1 eq) were combined and azeotroped ×3 with toluene and placed under high vacuum overnight. The mixture was dissolved in dry DMF and cannulated to the 10 mL microwave tube with activated 4 A° molecular sieves and it was placed under Ar. The indicated solvent was added (Total concentration-0.14 M) to the reaction mixture and the tube was capped with the microwave vessel cap. The reaction mixture was subjected to microwave heating as indicated in Table 2 (150-250°C, 250 PSI, 300W, 1-2 hours. Measurements supplied by the instrument). After completion of the reaction, the residue was diluted with CH_2Cl_2, washed with ice cold water (×2) to remove 2, 4-Dinitro phenol and DMF and followed by sat. NaCl. The organic layer was dried over Na_2SO_4 and the solvent was removed under reduced pressure to obtain the unpurified compound. Purification of the crude product was done using silica gel chromatography with hexane and ethyl acetate to afford glycosylated product.

Methyl 2,3,4,6-tetra-*O*-benzyl-*α*-D-galactopyranoside (4*α*)

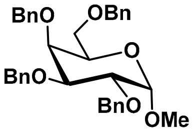

The title compound **4** was obtained from donor **3** in MeOH under the microwave conditions.

The glycosyl donor (100. mg, 0.14 mmol, 1.2 eq), and acceptor (1 eq) was azeotroped ×3 with toluene and placed under high vacuum overnight. The mixture was dissolved in dry MeOH and cannulated to the 10 mL microwave tube with activated 4 A° molecular sieves and it was placed under Argon. The tube was capped with the microwave vessel cap. The reaction mixture was subjected to microwave heating as indicated in Table 1 (150-250°C, 250 PSI, 300W, 1-2 hours. Measurements supplied by the instrument). After completion of the reaction, the residue was

diluted with CH_2Cl_2, washed with ice cold water (×2) to remove 2, 4-Dinitro phenol and followed by sat. NaCl. The organic layer was dried over Na_2SO_4 and the solvent was removed under reduced pressure to obtain the unpurified compound. Purification of the crude product was done using silica gel chromatography with hexane and ethyl acetate to afford glycosylated product. The isolated amount of 4α was 49.4 mg, corresponding to a 63% yield, and 4β was isolated in 16.0 mg, corresponding to a 21% yield from α donor. The isolated amount of 4α was 68.4 mg, corresponding to a 87% yield from β donor. The isolated amount of 4α was 35,9 mg, corresponding to a 45.7% yield, and 4β was isolated in 27.6 mg, corresponding to a 35.2 % yield from (1:1) α/β donors.

Analytical data for **4*α*** were in accordance with those reported previously [30].

4*α*

Rf = 0.51 (hexane/EtOAc, 2:1)

^{1}H NMR (600 MHz, $CDCl_3$):- δ 7.42 – 7.22 (m, 1H, 20H, Ar-H), 4.94 (d, *J* = 11.4 Hz, 1H, PhC*H*$_2$), 4.85 (d, *J* = 7.5 Hz, 1H, PhC*H*$_2$), 4.83 (d, *J* = 7.9 Hz, 1H, PhC*H*$_2$), 4.73 (d, *J* = 11.7 Hz, 1H, PhC*H*$_2$), 4.68 (d, *J* = 3.5 Hz, 1H, H^1), 4.69 (d, *J* = 12.2 Hz, 1H, PhC*H*$_2$), 4.57 (d, *J* = 11.6 Hz, 1H, PhC*H*$_2$), 4.48 (d, *J* = 11.7 Hz, 1H, PhC*H*$_2$), 4.39 (d, *J* = 11.7 Hz, 1H, PhC*H*$_2$), 4.03 (ddd, *J* = 5.4, 4.5, 2.0 Hz, 1H, H^2), 3.93 – 3.91(m, 2H, H^3 and H^4), 3.89 (t, *J* = 6.5 Hz, 1H, H^5), 3.52 (d, *J* = 6.4 Hz, 2H, H^6), 3.37 (s, 3H, OCH_3).

^{13}C NMR (151 MHz, $CDCl_3$):- δ 138.9 – 138.1 (Ar-C), 128.5 – 127.6 (Ar-C), 98.9 (C^1), 79.3 (C^3), 76.5 (C^2), 75.3 (C^4), 74.9 (Ph*C*H$_2$), 73.7 (Ph*C*H$_2$), 73.6 (Ph*C*H$_2$), 73.4 (Ph*C*H$_2$), 69.4 (C^5), 69.2 (C^6), 55.5 (OCH_3).

ESI-MS calcd. for $C_{35}H_{38}O_6^+$ [M+Na]$^+$: 577.68, found 577.40.

OR: $[\alpha]_D^{22.5}$ = +100.0 (C = 1 mg/1 mL in $CHCl_3$)

Methyl 2,3,4,6-tetra-*O*-benzyl-*β*-D-galactopyranoside (4*β*)

The title compound **4** was obtained from donor **3** in MeOH under the microwave conditions.

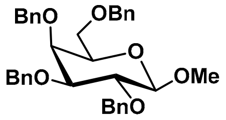

<u>4*β*</u>

Rf = 0.57 (hexane/EtOAc, 2:1).

<u>^{1}H NMR (600 MHz, CDCl$_3$)</u>:- δ 7.39 – 7.23 (m, 1H, 20H, Ar-H), 4.94 (d, *J* = 11.6 Hz, 1H, PhC<u>*H*</u>$_2$), 4.89 (d, *J* = 10.8 Hz, 1H, PhC<u>*H*</u>$_2$), 4.77 – 4.69 (m, 3H, PhC<u>*H*</u>$_2$), 4.61 (d, *J* = 11.7 Hz, 1H, PhC<u>*H*</u>$_2$), 4.43 (q, *J* = 11.7 Hz, 2H, PhC<u>*H*</u>$_2$), 4.27 (d, *J* = 7.7 Hz, 1H, H^1), 3.89 (d, *J* = 2.8 Hz, 1H, H^4), 3.80 (dd, *J* = 9.7, 7.7 Hz, 1H, H^2), 3.59 (m, 2H, H^6), 3.55 (s, 3H, OCH_3), 3.54 – 3.50 (m, 2H, H^3, H^5).

<u>^{13}C NMR (151 MHz, CDCl$_3$)</u>:- δ 138.9 - 138.0 (Ar-C), 128.5 - 127.6(Ar-C), 105.1 (C^1), 82.2 (C^3), 79.7 (C^2), 75.3 (Ph<u>C</u>*H*$_2$), 74.5 (Ph<u>C</u>*H*$_2$), 73.7 (Ph<u>C</u>*H*$_2$), 73.5 (C^5), 73.5 (Ph<u>C</u>*H*$_2$), 73.1 (C^4), 68.9 (C^6), 57.1 (OCH_3).

ESI-MS <u>calcd</u>. for $C_{35}H_{38}O_6^+$ $[M+Na]^+$: 577.68, found 577.40.

OR: $[\alpha]_D^{22.5}$ = +11.0 (C = 1 mg/ 1 mL in CHCl$_3$)

Methyl (2,3,4,6-tetra-*O*-benzyl-*α*-D-galactopyranosyl) -(1à6)-2,3,4-tri-*O*-benzyl-*α*-D-galactopyranoside (6*α*)

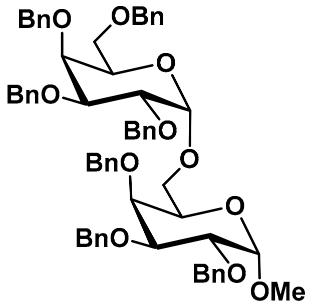

The glycosyl donor (100. mg, 0.14 mmol, 1.2 eq), and acceptor (54.0 mg, 0.118 mmol, 1 eq) were combined and azeotroped ×3 with toluene and placed under high vacuum overnight. The mixture was dissolved in dry DMF, 2 mL (Total concentration-0.15 M) and cannulated to the 10 mL microwave tube with activated 4 A° molecular sieves and it was placed under Ar. The tube was capped with the microwave vessel cap. The reaction mixture was subjected to microwave heating as indicated in Table 2 (150-250°C, 250 PSI, 300W, 1-2 hours. Measurements supplied by the instrument). After completion of the reaction, the residue was diluted with CH_2Cl_2, washed with ice cold water (×2) to remove 2, 4-Dinitro phenol and DMF and followed by sat. NaCl. The organic layer was dried over Na_2SO_4 and the solvent was removed under reduced pressure to obtain the unpurified compound. Purification of the crude product was done using silica gel chromatography with hexane and ethyl acetate to afford glycosylated product. The isolated amount of 6α was 84.0 mg, corresponding to a 60% yield and 6β was isolated in 28.0 mg, corresponding to a 20% yield from α donor. The isolated amount of 6α was 90.0 mg, corresponding to a 64.5% yield and 6β was isolated in 30.0 mg, corresponding to a 21.5% yield from β donor.

Rf = 0.56 (hexane/EtOAc, 2:1)

^{1}H NMR (600 MHz, $CDCl_3$):- δ 7.42 – 7.23 (m, 35H), 4.95 (d, *J* = 11.4 Hz, 1H), 4.93 (d, *J* = 11.4 Hz, 1H), 4.86 – 4.82 (m, 1H), 4.80 (d, *J* = 11.9 Hz, 1H), 4.78 – 4.74 (m, 2H), 4.72 (d, *J* = 11.8 Hz, 1H), 4.69 (d, *J* = 12.1 Hz, 1H), 4.65 (d, *J* = 12.0 Hz, 1H), 4.64 (d, *J* = 3.6 Hz, 1H) 4.58 (t, *J* = 11.2 Hz, 1H), 4.48 (d, *J* = 11.7 Hz, 1H), 4.40 (d, *J* = 11.7 Hz, 1H), 4.05 – 4.01 (m, 2H), 4.01 – 4.00 (m, 1H), 3.99 – 3.96 (m, 1H), 3.96 – 3.89 (m, 4H), 3.73 (dd, *J* = 10.0, 6.3 Hz, 1H), 3.58 (dd, *J* = 9.1, 7.7 Hz, 1H), 3.52 (dd, *J* = 9.1, 5.6 Hz, 1H), 3.44 (dd, *J* = 9.9, 6.0 Hz, 1H), 3.29 (s, 3H).

^{13}C NMR (151 MHz, $CDCl_3$):- δ 138.9, 138.8, 138.7, 138.7, 138.6, 138.0, 128.5, 128.4, 128.4, 128.4, 128.4, 128.3, 128.2, 128.2, 127.9, 127.9, 127.8, 127.7, 127.7, 127.6, 127.6, 127.5, 98.7, 98.2, 79.1, 78.9, 77.3, 77.1, 76.9, 76.4, 76.2, 75.4, 74.8, 74.7, 73.6, 73.6, 73.5, 73.3, 72.7, 69.3, 69.2, 68.7, 67.3, 55.3.

OR: $[\alpha]_D^{22.5}$ = +103.0 (C = 0.0825 g/100 mL in $CHCl_3$)
ESI-MS calcd. for $C_{62}H_{66}O_{11}^+$ $[M+K]^+$: 1026.20, found 1026.86.

Methyl (2,3,4,6-tetra-*O*-benzyl-*β*-D-galactopyranosyl)-(1à6)-2,3,4-tri-*O*-benzyl-*α*-D-galactopyranoside (6b)

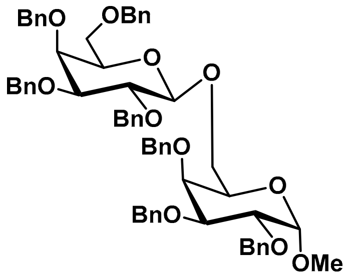

Rf= 0.57 (hexane/EtOAc, 2:1)

<u>^{1}H NMR (600 MHz, CDCl3)</u>:- δ 7.42 – 7.19 (m, 35H), 4.96 (t, *J* = 11.7 Hz, 2H), 4.90 (d, *J* = 10.8 Hz, 1H), 4.85 (dd, *J* = 12.1, 9.9 Hz, 2H), 4.76 (d, *J* = 11.9 Hz, 1H), 4.73 (d, *J* = 2.5 Hz, 1H), 4.72 – 4.71 (m, 1H), 4.70 – 4.68 (d, m, 2H), 4.64 (dd, *J* = 11.6, 2.7 Hz, 1H), 4.42 (s, 2H), 4.36 (d, *J* = 7.7 Hz, 1H), 4.05 (dd, *J* = 10.1, 3.6 Hz, 1H), 3.95 – 3.91 (m, 3H), 3.85 (d, *J* = 3.8 Hz, 1H), 3.82 (dd, *J* = 9.9, 4.2 Hz, 1H), 3.79 – 3.77 (m, 1H), 3.69 (dd, *J* = 10.3, 7.3 Hz, 1H), 3.61 – 3.57 (m, 1H), 3.55 – 3.51 (m, 1H), 3.30 (s, 3H).

<u>^{13}C NMR (151 MHz, CDCl3)</u>:- δ 138.8, 138.7, 138.7, 138.5, 138.5, 138.5, 137.8, 128.5, 128.5, 128.4, 128.4, 128.4, 128.3, 128.3, 128.2, 128.2, 128.2, 128.0, 127.9, 127.8, 127.7, 127.6, 127.6, 103.9, 98.7, 82.1, 79.7, 79.1, 77.3, 77.1, 76.9, 76.4, 75.3, 75.2, 74.6, 74.6, 73.6, 73.6, 73.4, 73.3, 73.1, 73.0, 69.6, 69.0, 68.4, 55.4.

ESI-MS calcd. for $C_{62}H_{66}O_{11}^{+}$ [M+K]$^{+}$: 1026.20, found 1026.86.

2,3,4,6-tetra-*O*-benzyl-*α*-D-galactopyranosyl-(1à6)-1,2:3,4-di-*O*-isopropylidene-*α*-D-galactopyranoside (13*α*)

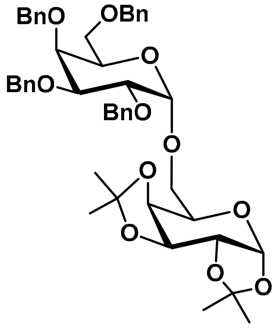

The title compound **13** was obtained from donor **3** (100. mg, 0.142 mmol, 1.2 eq) with acceptor **7** (30.5 mg, 0.118 mmol, 1 eq) under the microwave conditions in 2 mL DMF. The isolated amount of 13α was 56.0 mg, corresponding to a 51% yield and the amount of 13β was isolated in 18.8.0 mg, corresponding to a 17% yield from α donor. The isolated amount of 13α was 45.0 mg, corresponding to a 40.6 % yield and the isolated amount of 14β was 21.4 mg, corresponding to a 19.3% yield from β donor.

Rf= 0.66 (hexane/EtOAc, 2:1)

<u>^{1}H NMR (600 MHz, CDCl$_3$)</u>:- δ 7.40 – 7.22 (m, 20H), 5.51 (d, *J* = 5.1 Hz, 1H), 5.01 (d, *J* = 3.7 Hz, 1H), 4.93 (d, *J* = 11.4 Hz, 1H), 4.83 (d, *J* = 11.6 Hz, 1H), 4.74 (s, 2H), 4.73 (d, *J* = 11.6 Hz, 1H), 4.58 (d, *J* = 7.5 Hz, 1H), 4.56 (dd, *J* = 5.9, 2.0 Hz, 1H), 4.44 (dd, *J* = 39.1, 11.7 Hz, 2H), 4.31 (dd, *J* = 7.9, 2.0 Hz, 1H), 4.29 (dd, *J* = 5.0, 2.4 Hz, 1H), 4.07 – 4.01 (m, 4H), 3.95 (dd, *J* = 10.1, 2.7 Hz, 1H), 3.78 (dd, *J* = 10.5, 6.4 Hz, 1H), 3.73 (dd, *J* = 10.4, 7.2 Hz, 1H), 3.56 (dd, *J* = 9.1, 7.5 Hz, 1H), 3.51 (dd, *J* = 9.2, 5.7 Hz, 1H), 1.51 (s, 3H), 1.42 (s, 3H), 1.32 (s, 3H), 1.29 (s, 3H).

<u>^{13}C NMR (151 MHz, CDCl$_3$)</u>:- δ 139.0, 138.8, 138.2, 128.7, 128.5, 128.4, 128.3, 128.3, 127.9, 127.8, 127.8, 127.6, 127.6, 127.5, 127.5, 127.1, 109.3, 108.6, 97.6, 96.4, 79.1, 77.3, 77.1, 76.9, 76.5, 75.0, 74.9, 73.5, 73.1, 72.8, 71.0, 70.7, 70.7, 69.2, 68.7, 66.4, 65.9, 26.2, 26.1, 25.0, 24.7.

ESI-MS <u>calcd</u>. for $C_{46}H_{54}O_{11}^+$ [M+Na]$^+$: 805.93, found 805.37.

2,3,4,6-tetra-*O*-benzyl-*β*-D-galactopyranosyl -(1à6)-1,2:3,4-di-*O*-isopropylidene-*α*-D-galactopyranoside (13b)

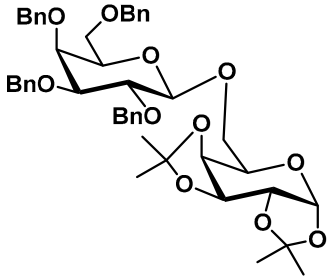

Rf= 0.66 (hexane/EtOAc, 2:1)

^{1}H NMR: (600 MHz, $CDCl_3$) δ 7.47 – 7.21 (m, 20H), 5.56 (d, *J* = 4.9 Hz, 1H), 5.05 (d, *J* = 11.0 Hz, 1H), 4.93 (d, *J* = 11.7 Hz, 1H), 4.79 (d, *J* = 11.7 Hz, 1H), 4.72 (t, *J* = 12.1 Hz, 2H), 4.61 (d, *J* = 11.7 Hz, 1H), 4.57 (dd, *J* = 8.0, 2.5 Hz, 1H), 4.41 (q, *J* = 11.7 Hz, 1H), 4.41 (d, *J* = 7.7 Hz, 1H), 4.30 (dd, *J* = 4.9, 2.4 Hz, 1H), 4.21 (dd, *J* = 8.0, 1.9 Hz, 1H), 4.13 (dd, *J* = 10.6, 3.5 Hz, 1H), 4.09 – 4.04 (m, 1H), 3.88 (d, *J* = 3.0 Hz, 1H), 3.82 (dd, *J* = 9.8, 7.6 Hz, 1H), 3.68 (dd, *J* = 10.8, 7.5 Hz, 1H), 3.59 – 3.56 (m, 2H), 3.54 – 3.51 (m, 2H) 1.49 (s, 3H), 1.43 (s, 3H), 1.31 (s, 3H), 1.30 (s, 3H).

^{13}C NMR: (150 MHz, $CDCl_3$) δ 139.1, 138.8, 138.0, 128.7, 128.5, 128.5, 128.4, 128.2, 128.0, 127.9, 127.6, 127.4, 109.4, 108.7, 104.8, 96.5, 82.0, 79.2, 77.3, 77.1, 76.9, 74.9, 74.6, 73.6, 73.4, 73.2, 71.6, 70.9, 70.6, 69.7, 68.7, 67.5, 26.1, 26.1, 25.2, 24.5.

OR: $[\alpha]_D^{22.5}$ = -61.4 (C =0.070 g/100 mL in $CHCl_3$)

ESI-MS calcd. for $C_{46}H_{54}O_{11}^+$ [M+Na]$^+$: 805.93, found 805.37.

Methyl (2,3,4,6-tetra-*O*-benzyl-*α*-D-galactopyranosyl)-(1à2)-3,4,6-tri-*O*-benzyl-*β*-D-glucopyranoside (14*α*)

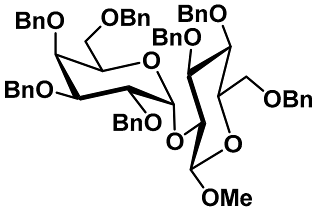

The title compound **14** was obtained from donor **3** (100. mg, 0.142 mmol, 1.2 eq) with acceptor **8** (54.0 mg, 0.118 mmol, 1 eq) under the microwave conditions in 2 mL DMF. The isolated amount of 14*α* was 60.8 mg, corresponding to a 43.5% yield and the isolated amount of 14β was 20.2 mg, corresponding to a 14.5% yield from β donor. The isolated amount of 14*α* was 90.8 mg, corresponding to a 65% yield from *α* donor.

Rf - 0.73 (hexane/EtOAc, 2:1)

^{1}H NMR: (600 MHz, $CDCl_3$):- δ 7.42 – 7.29 (m, 20H), 7.28 –7.27 (m, 13H), 7.20 – 7.19 (m, 3H), 7.13 – 7.08 (m, 2H), 5.66 (d, *J* = 3.7 Hz, 1H), 4.92 (d, *J* = 11.5 Hz, 1H), 4.89 (d, *J* = 11.4 Hz, 1H), 4.83 (d, *J* = 11.8 Hz, 1H), 4.81 (s, 2H), 4.79 (d, *J* = 11.5 Hz, 1H), 4.74 (d, *J* = 10.7 Hz, 1H), 4.71 (d, *J* = 11.8 Hz,

1H), 4.66 (d, *J* = 12.2 Hz, 1H), 4.59 (d, *J* = 12.2 Hz, 1H), 4.53 (d, *J* = 10.7 Hz, 1H), 4.50 (d, *J* = 11.4 Hz, 1H), 4.44 (d, *J* = 7.7 Hz, 1H), 4.31 (d, *J* = 11.8 Hz, 1H), 4.26 – 4.22 (m, 2H), 4.08 (dd, *J* = 10.2, 3.7 Hz, 1H), 3.88 (dd, *J* = 10.2, 2.8 Hz, 1H) 3.81 – 3.69 (m, 5H), 3.63 (t, *J* = 9.4 Hz, 1H), 3.54 (s, 3H), 3.52 – 3.46 (m, 2H), 3.35 (dd, *J* = 9.3, 7.2 Hz, 1H).

^{13}C NMR (151 MHz, $CDCl_3$):- 139.0, 138.9, 138.7, 138.7, 138.5, 138.2, 138.0, 128.5, 128.4, 128.4, 128.3, 128.2, 128.2, 128.1, 128.1, 127.9, 127.9, 127.8, 127.7, 127.6, 127.6, 127.5, 127.5, 127.4, 127.4, 104.7, 95.8, 83.5, 78.8, 78.5, 77.3, 77.1, 76.9, 76.2, 75.7, 75.3, 75.0, 75.0, 74.8, 74.5, 73.6, 72.9, 72.8, 68.9, 68.8, 56.7.

OR: $[\alpha]_D^{22.5}$ = +32.9 (C = 0.070 g/100 mL in $CHCl_3$)

ESI-MS calcd. for $C_{62}H_{66}O_{11}^+$ [M+K]$^+$: 1026.20, found 1026.86.

Methyl (2,3,4,6-tetra-*O*-benzyl-*β*-D-galactopyranosyl)-(1à2)-3,4,6-tri-*O*-benzyl-*β*-D-glucopyranoside (14b)

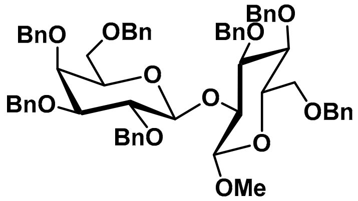

Rf = 0.56 (hexane/EtOAc, 2:1)

^{1}H NMR (600 MHz, $CDCl_3$):- δ 7.39 – 7.21 (m, 30H), 7.20 – 7.16 (m, 3H), 7.13 (dd, *J* = 6.5, 3.2 Hz, 2H), 4.96 (d, *J* = 11.6 Hz, 1H), 4.92 (t, *J* = 10.8 Hz, 2H), 4.86 (d, *J* = 7.9 Hz, 1H), 4.79 (t, *J* = 10.1 Hz, 2H), 4.73 -4.71 (m, 3H), 4.64 (d, *J* = 12.3 Hz, 2H), 4.57 (dd, *J* = 11.5, 9.6 Hz, 2H), 4.44 (d, *J* = 1.9 Hz, 2H), 4.42 (d, *J* = 7.3 Hz, 1H), 3.90 (d, *J* = 3.3 Hz, 1H), 3.85 – 3.80 (m, 2H), 3.77 – 3.68 (m, 3H), 3.67 – 3.61 (m, 2H), 3.58 (dd, *J* = 9.1, 5.3 Hz, 1H), 3.52 – 3.46 (m, 6H).

^{13}C NMR (151 MHz, $CDCl_3$):- δ 139.0, 138.9, 138.7, 138.7, 138.5, 138.2, 138.0, 128.6, 128.5, 128.4, 128.4, 128.4, 128.3, 128.2, 128.1, 128.1, 128.0, 127.9, 127.9, 127.7, 127.6, 127.6, 127.5, 127.5, 127.4, 127.4, 104.8, 95.8, 83.5, 78.8, 78.5, 77.6, 77.3, 77.1, 76.9, 76.2, 75.8, 75.3, 75.0, 75.0, 74.8, 74.6, 73.6, 73.0, 72.8, 68.9, 68.8, 56.7, 29.8.

ESI-MS calcd. for $C_{62}H_{66}O_{11}^+$ [M+K]$^+$: 1026.20, found 1026.86.

Methyl (2,3,4,6-tetra-*O*-benzyl-α-D-galactopyranosyl)-(1à3)-2,4,6-tri-*O*-benzyl-β-D-glucopyranoside (15α)

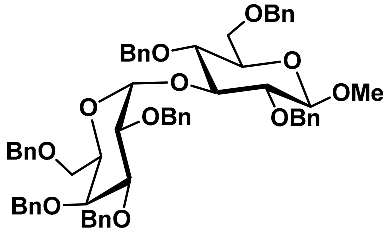

The title compound **15** was obtained from donor **3** (100. mg, 0.142 mmol, 1.2 eq) with acceptor **9** (54.0 mg, 0.118 mmol, 1 eq) under the microwave conditions in 2 mL DMF. The isolated amount of 15α was 60.5 mg, corresponding to a 43.3% yield and the isolated amount of 15β was 30.2 mg, corresponding to a 21.6% yield from β donor. The isolated amount of 15α was 67.4 mg, corresponding to a 48.2% yield and the isolated amount of 15β was 19.2 mg, corresponding to a 13.7% yield from α donor.

Rf = 0.34 (hexane/EtOAc, 4:1)

<u>^{1}H NMR (600 MHz, $CDCl_3$)</u>:- δ 7.42 – 7.24 (m, 28H), 7.22 – 7.17 (m, 3H), 7.14 – 7.10 (m, 2H), 5.01 (d, *J* = 3.6 Hz, 1H), 4.94 (d, *J* = 3.4 Hz, 1H), 4.91 (d, *J* = 11.4 Hz, 1H), 4.86 (d, *J* = 5.7 Hz, 1H), 4.83 (d, *J* = 12.3 Hz, 1H), 4.80 (d, *J* = 4.8 Hz, 1H), 4.78 (d, *J* = 5.7 Hz, 1H), 4.73 (t, *J* = 7.5 Hz, 1H), 4.69 (d, *J* = 11.9 Hz, 1H), 4.64 (d, *J* = 12.1 Hz, 1H), 4.53 (d, *J* = 6.0 Hz, 1H), 4.52 (d, *J* = 5.3 Hz, 1H), 4.48 (d, *J* = 11.0 Hz, 1H), 4.35 (d, *J* = 11.7 Hz, 1H), 4.30 (d, *J* = 11.9 Hz, 1H), 4.16 (t, *J* = 6.8 Hz, 1H), 4.09 – 4.02 (m, 2H), 3.93 (dd, *J* = 10.1, 2.9 Hz, 1H), 3.88 (dd, *J* = 9.9, 3.5 Hz, 1H), 3.79 (ddd, *J* = 10.1, 4.0, 2.1 Hz, 1H), 3.76 – 3.73 (m, 2H), 3.68 (dd, *J* = 10.6, 2.1 Hz, 1H), 3.65 – 3.60 (m, 1H), 3.49 (dd, *J* = 9.5, 6.1 Hz, 1H), 3.43 (s, 3H), 3.39 (dd, *J* = 9.5, 7.2 Hz, 1H).

<u>^{13}C NMR (151 MHz, $CDCl_3$)</u>:- δ 138.8, 138.8, 138.7, 138.6, 138.3, 138.2, 137.9, 128.4, 128.3, 128.3, 128.3, 128.2, 128.2, 128.1, 128.1, 127.9, 127.9, 127.8, 127.7, 127.6, 127.5, 127.5, 127.5, 127.4, 127.4, 127.3, 96.5, 94.7, 80.9, 78.7, 77.9, 77.2, 77.0, 76.8, 75.8, 75.6, 74.9, 74.7, 74.6, 73.5, 72.9, 72.8, 70.2, 69.0, 68.6, 68.5, 54.9.

ESI-MS <u>calcd</u>. for $C_{62}H_{66}O_{11}^+$ [M+K]$^+$: 1026.20, found 1026.80.

OR: $[\alpha]_D^{22.5}$ = +17.1(C = 0.18 g/100 mL in $CHCl_3$)

Methyl (2,3,4,6-tetra-*O*-benzyl-*β*-D-galactopyranosyl)-(1à3)-2,4,6-tri-O-benzyl-β-D-glucopyranoside (15b)

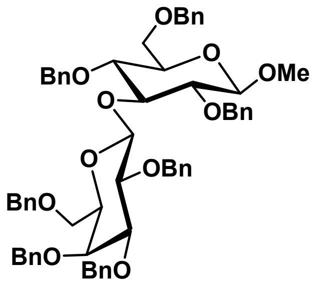

Rf = 0.40 (hexane/EtOAc, 4:1)

^{1}H NMR (600 MHz, $CDCl_3$):- δ 7.37 – 7.29 (m, 20H), 7.27 (dd, *J* = 5.1, 1.9 Hz, 5H), 7.24 – 7.15 (m, 8H), 7.13 – 7.09 (m, 2H), 5.01 (d, *J* = 11.3 Hz, 1H), 4.94 (d, *J* = 11.6 Hz, 1H), 4.90 (d, *J* = 3.5 Hz, 1H), 4.87 (t, *J* = 11.1 Hz, 1H), 4.81 (d, *J* = 10.8 Hz, 1H), 4.75 (d, *J* = 11.9 Hz, 1H), 4.70 (d, *J* = 7.8 Hz, 1H), 4.71 (d, *J* = 11.9 Hz, 1H), 4.68 – 4.64 (m, 1H), 4.62 (d, *J* = 11.6 Hz, 1H), 4.52 (d, *J* = 12.1 Hz, 1H), 4.48 (d, *J* = 10.8 Hz, 1H), 4.45 – 4.40 (m, 2H), 4.04 (t, *J* = 9.3 Hz, 1H), 3.92 – 3.87 (m, 1H), 3.84 (dd, *J* = 9.8, 3.5 Hz, 1H), 3.83 – 3.79 (m, 1H), 3.75 (dd, *J* = 10.6, 3.7 Hz, 1H), 3.71 – 3.63 (m, 2H), 3.59 (dd, *J* = 8.8, 7.7 Hz, 1H), 3.53 (dd, *J* = 8.9, 5.3 Hz, 1H), 3.51 – 3.45 (m, 2H), 3.41 (s, 3H).

^{13}C NMR (151 MHz, $CDCl_3$):- δ 138.9, 138.8, 138.8, 138.5, 138.3, 138.1, 137.9, 128.6, 128.5, 128.4, 128.4, 128.3, 128.2, 128.1, 128.1, 128.1, 128.0, 127.9, 127.8, 127.7, 127.6, 127.6, 127.4, 127.2, 104.2, 99.8, 82.4, 82.0, 79.3, 78.3, 77.3, 77.1, 76.9, 75.2, 75.1, 74.7, 73.7, 73.6, 73.3, 73.0, 70.0, 68.7, 68.5, 55.2.

OR: $[\alpha]_D^{22.5}$ = -40.0 (C = 0.30 g/100 mL in $CHCl_3$)

ESI-MS calcd. for $C_{62}H_{66}O_{11}^+$ [M+K]$^+$: 1026.20, found 1026.80.

1-Adamantanyl 2,3,4,6-tetra-O-benzyl-α-D-galactopyranoside (17)

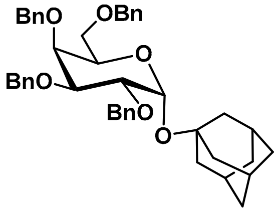

The title compound **17** was obtained from donor **3** (100. mg, 0.142 mmol, 1.2 eq) and acceptor **11** (17.8 mg, 0.118 mmol, 1 eq) in 2 mL DMF under the general glycosylation method. Analytical data for were in accordance with those reported previously [31]. The isolated amount of 17α was 66.9 mg, corresponding to a 70% yield from β donor. The isolated amount of 17α was 62.0 mg, corresponding to a 65% yield from α donor.

Rf = 0.40 (hexane/EtOAc, 4:1)

<u>^{1}H NMR (600 MHz, $CDCl_3$)</u> :- δ 7.41 – 7.22 (m, 20H), 5.31 (d, *J* = 3.5 Hz, 1H), 4.96 (d, *J* = 11.4 Hz, 1H), 4.84 (d, *J* = 11.6 Hz, 1H), 4.74 (dd, *J* = 11.7, 7.5 Hz, 2H), 4.69 (d, *J* = 11.9 Hz, 1H), 4.56 (d, *J* = 11.4 Hz, 1H), 4.48 (d, *J* = 11.7 Hz, 1H), 4.41 (d, *J* = 11.7 Hz, 1H), 4.18 (t, *J* = 6.6 Hz, 1H), 4.02 – 3.96 (m, 3H), 3.57 (dd, *J* = 9.1, 7.6 Hz, 1H), 3.50 (dd, *J* = 9.2, 5.9 Hz, 1H), 2.12 (s, 3H), 1.83 (dd, *J* = 45.7, 11.4 Hz, 6H), 1.64 – 1.58 (m, 8H).

<u>^{13}C NMR (151 MHz, $CDCl_3$)</u>:- δ 139.1, 139.0, 138.9.138.2, 128.4, 128.4, 128.2, 128.2, 128.0, 127.7, 127.6, 127.5, 127.5, 127.4, 90.6, 79.3, 76.7, 75.3, 74.8, 74.3, 73.5, 73.1, 73.0, 69.2, 68.8, 42.5, 36.4, 30.7.

ESI-MS <u>calcd</u>. for $C_{44}H_{50}O_6^+$ [M+Na]$^+$: 697.88, found 697.36.

Cholesteryl 2,3,4,6-tetra-O-benzyl-α-D-galactopyranoside (18)

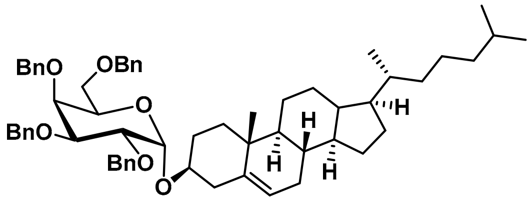

The title compound **17** was obtained from donor **3** (100. mg, 0.142 mmol, 1.2 eq) and acceptor **11** (43.7 mg, 0.118 mmol, 1 eq) in 2 mL DMF under the general glycosylation method. The isolated amount of 18α was 101.4 mg, corresponding to a 80% yield from α donor. The isolated amount of 18α was 95.5 mg, corresponding to a 75% yield from β donor.

Rf- 0.89 (hexane/EtOAc, 2:1)

^{1}<u>H NMR (600 MHz, CDCl$_3$)</u>:- δ 7.42 – 7.22 (m, 20H), 5.32 (dd, *J* = 5.0, 2.5 Hz, 0.3H), 5.25 (dd, *J* = 5.2, 2.5 Hz, 1H), 4.98 (d, *J* = 3.8 Hz, 1H), 4.96 – 4.91 (m, 2H), 4.85 (d, *J* = 11.7 Hz, 1H), 4.79 (d, *J* = 12.1 Hz, 1H), 4.73 (d, *J* = 8.8 Hz, 1H), 4.70 (d, *J* = 11.8 Hz, 0.4H), 4.67 (d, *J* = 11.9 Hz, 1H), 4.61 (d, *J* = 11.7 Hz, 0.4H), 4.57 (d, *J* = 11.6 Hz, 1H), 4.47 (d, *J* = 11.7 Hz, 1H), 4.45 (d, *J* = 7.8 Hz, 1H) 4.40 (t, *J* = 8.7 H, 1.5H), 4.05 (t, *J* = 6.6 Hz, 1H), 4.02 (dd, *J* = 10.0, 3.7 Hz, 1H), 3.98 (d, *J* = 3.2 Hz, 1H), 3.95 (dd, *J* = 10.1, 2.9 Hz, 1H), 3.86 (d, *J* = 3.0 Hz, 0.4H), 3.79 (dd, *J* = 9.8, 7.7 Hz, 0.4H), 3.56 (t, *J* = 4.7 Hz, 1H), 3.53 (d, *J* = 6.6 Hz, 2H), 3.50 (dd, *J* = 7.0, 2.9 Hz, 0.4H), 3.48 – 3.43 (m, 1H), 2.45 – 0.65 (m, 1H)

^{13}<u>C NMR (151 MHz, CDCl$_3$)</u>:- δ 141.0, 139.1, 138.8, 138.8, 138.1, 128.5, 128.5, 128.4, 128.4, 128.3, 128.3, 128.2, 128.1, 128.0, 127.8, 127.7, 127.7, 127.6, 127.5, 127.5, 121.8, 102.5, 95.6, 82.5, 79.8, 79.5, 79.3, 77.3, 77.1, 76.9, 76.8, 76.6, 75.4, 75.3, 74.8, 74.5, 73.6, 73.5, 73.5, 73.3, 73.2, 69.3, 69.2, 69.1, 56.9, 56.2, 50.3, 50.2, 42.4, 40.0, 39.9, 39.6, 39.1, 37.4, 37.2, 36.9, 36.3, 35.9, 32.0, 32.0, 29.9, 28.3, 28.1, 27.7, 24.4, 23.9, 22.9, 22.7, 21.2, 19.5, 18.8, 12.0.

ESI-MS <u>calcd</u>. for $C_{60}H_{78}O_6^+$ [M+K]$^+$: 934.28, found 934.58.

Trisaccharide synthesis

2, 4-Dinitrophenyl (2,3,4,6-tetra-*O*-benzyl-*α*-D-galactopyranosyl)-2,3,4-tri-*O*-benzyl-*α*/*β*-D galactopyranoside (19)

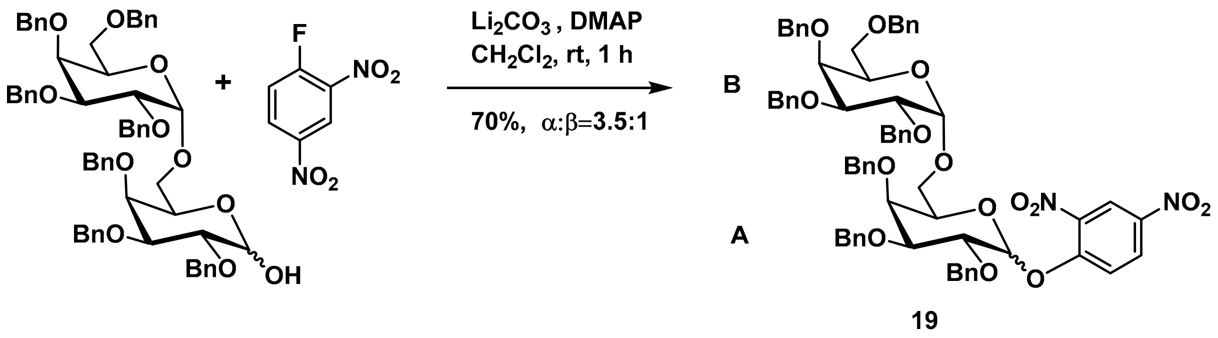

The titled compound **19** was synthesized according to the general glycosyl donor synthesis method. Compound **S12** (1.00 g, 1.02 mmol, 1 eq), Li_2CO_3 (0.150 g, 2.05 mmol, 2 eq.), and Dinitrofluorobenzene (0.229 g, 1.23 mmol, 1.2 eq.) were dissolved in dry CH_2Cl_2 (10 mL) in a dried flask and the reaction mixture was stirred at room temperature. DMAP (0.125 mg, 1.03 mmol, 1 eq.) was dissolved in CH_2Cl_2 and added to the suspension over 20 minutes in 5 portions. Upon addition of DMAP, the suspension changed color to a strong yellow brown to dark red. The reaction was allowed to stir at room temperature for 1–2 hours. After TLC indicated complete consumption of the starting materials, the product was purified by silica gel chromatography using a hexane: ethyl acetate gradient. The isolated amount of 19*α* was 0.63 g, corresponding to a 55% yield and the isolated amount of 19β was 0.18 g, corresponding to a 15% yield.

2, 4-Dinitrophenyl (2,3,4,6-tetra-*O*-benzyl-*α*-D-galactopyranosyl)-2,3,4-tri-*O*-benzyl-*α*-D-galactopyranoside (19a)

19a- Rf =0.77 (hexane/EtOAc, 3:1).

<u>^{1}H NMR (600 MHz, $CDCl_3$)</u>:- δ 8.62 (d, *J* = 2.9 Hz, 1H), 8.11 (dd, *J* = 9.3, 2.8 Hz, 1H), 7.42 – 7.16 (m, 36H), 7.12 (d, *J* = 9.4 Hz, 1H), 5.51 (d, *J* = 3.3 Hz, 1H), 4.92 (d, *J* = 11.2 Hz, 1H), 4.90 (d, *J* = 11.6 Hz, 1H), 4.87 (dd, *J* = 12.0, 3.4 Hz, 2H), 4.77 – 4.72 (m, 2H), 4.65 (d, *J* = 3.7 Hz, 1H), 4.62 (s, 2H), 4.56 (d, *J* = 12.3 Hz, 1H), 4.54 (d, *J* = 5.9 Hz, 1H), 4.52 (d, *J* = 5.3 Hz, 1H), 4.39 (d, *J* = 11.4 Hz, 1H), 4.32 (d, *J* = 11.6 Hz, 1H), 4.20 (dd, *J* = 10.0, 3.2 Hz, 1H), 4.16 (dd, *J* = 10.0, 2.6 Hz, 1H), 4.01 – 3.98 (m, 1H), 3.96 – 3.91 (m, 2H), 3.88 (dd, *J* = 2.9, 1.2 Hz, 1H), 3.82 – 3.78 (m, 1H), 3.65 (dd, *J* = 10.1, 6.4 Hz, 1H), 3.61 (dd, *J* = 10.1, 2.9 Hz, 1H), 3.52 (dd, *J* = 9.0, 7.7 Hz, 1H), 3.46 (dd, *J* = 9.0, 5.3 Hz, 1H), 3.39 (dd, *J* = 10.1, 5.5 Hz, 1H).

<u>^{13}C NMR (151 MHz, $CDCl_3$)</u>:- δ 154.7, 140.8, 139.5, 138.6, 138.5, 138.4, 137.9, 128.8, 128.6, 128.6, 128.5, 128.5, 128.4, 128.3, 128.2, 128.0, 127.9, 127.9, 127.8, 127.8, 127.6, 127.3, 121.8, 117.1, 98.2, 97.9, 79.1, 78.2, 77.3, 77.1, 76.9, 76.1, 75.9, 75.2, 75.0, 74.8, 74.3, 74.0, 73.7, 73.7, 73.6, 72.6, 72.4, 69.4, 68.8, 67.1, 53.5.

OR: $[\alpha]_D^{22.5}$ = +131.3 (C = 0.022 g/100 mL in $CHCl_3$)

HRMS calcd. for $C_{67}H_{66}N_2O_{15}^+$ $[M+Na]^+$: 1161.4463, found 1161.4320.

2, 4-Dinitrophenyl (2,3,4,6-tetra-*O*-benzyl-*α*-D-galactopyranosyl)-2,3,4-tri-*O*-benzyl-*β*-D galactopyranoside (19b)

19b Rf =0.77 (hexane/EtOAc, 3:1)

<u>^{1}H NMR (600 MHz, $CDCl_3$)</u>:- δ 8.55 (d, J=2.76 Hz, 1H), 8.01 (dd, J= 2.82, 9.36, 1H), 7.47-7.13 (m, 34H), 5.08 (d, J= 7.76 Hz, 1H), 4.99-4.94 (m, 3H), 4.90-4.84 (m, 4H), 4.81 (d, J=12.12 Hz, 1H), 4.74 (d, J=11.61 Hz, 1H), 4.72(d, J=3.72 Hz, 1H), 4.64 (t, J= 11.61 Hz, 2H), 4.55(d, J= 11.11 Hz, 1H), 4.26 (dd, J=11.52 Hz, 21.18 Hz, 2H), 4.19 (dd, J=7.68 Hz, 9.45 Hz, 1H), 4.03 (dd, J=3.6 Hz, 9.36 Hz, 1H), 3.90-3.88 (m, 2H), 3.82-3.74 (m, 4H), 3.60 (dd, J=2.76 Hz, 9.72 Hz, 1H), 3.44-3.41(m, 3H).

^{13}C NMR (151 MHz, CDCl$_3$):- δ 154.5, 138.6, 138.6, 138.4, 138.0, 137.6, 128.6, 128.5, 128.5, 128.5, 128.5, 128.4, 128.4, 128.2, 128.0, 128.0, 127.9, 127.8, 127.6, 121.7, 117.5, 100.9, 98.3, 81.6, 78.2, 77.3, 77.1, 76.9, 76.5, 75.8, 74.9, 74.7, 74.6, 74.1, 73.7, 73.5, 72.9, 69.6, 68.9, 68.1.

OR: $[\alpha]_D^{22.5}$ = +65.4 (C = 0.36 g/100 mL in CHCl$_3$)

HRMS calcd. for $C_{67}H_{66}N_2O_{15}^+$ [M+Na]$^+$: 1161.4463, found 1161.4320.

Methyl 2,3,4-tri-O-benzyl-6-O-[2,3,4-tri-O-benzyl-6-O-(2,3,4,6-tetra-O-benzyl-α-D-galactopyranosyl)-D-galactopyranosyl]-α-D-galactopyranoside (20)

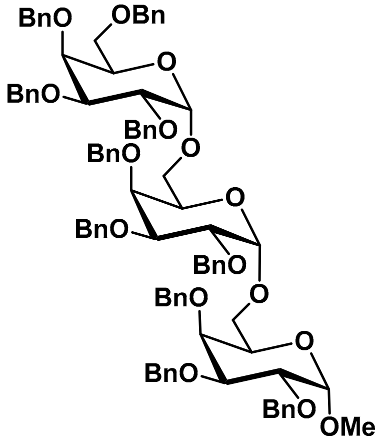

The title compound **20** was obtained from donor **19** (100. mg, 0.088 mmol, 1.2 eq) and acceptor **5** (0.338 mg, 0.073 mmol, 1 eq) in 2 mL DMF under the general glycosylation method. It was obtained as pale-yellow syrup and α product was isolated (isolated amount 49.8 mg) in 40% and (isolated amount 43.6 mg) 35% from α and β donors respectively.

Rf = 0.37 (hexane/EtOAc, 2:1)**^{1}H NMR (600 MHz, CDCl$_3$):-** δ 7.38 – 7.29 (m, 25H), 7.28 – 7.19 (m, 23H), 4.92 – 4.89 (m, 4H), 4.81 (dd, *J* = 9.0, 5.0 Hz, 2H), 4.80 – 4.76 (m, 4H), 4.76 – 4.74 (m, 3H), 4.71 (ddd, *J* = 9.8, 4.3, 3.0 Hz, 6H), 4.67 – 4.60 (m, 4H), 4.55 (t, *J* = 11.3 Hz, 4H), 4.51 – 4.47 (m, 2H), 4.42 – 4.33 (m, 2H), 4.04 (dd, *J* = 10.0, 3.6 Hz, 2H), 4.02 – 4.00 (m, 2H), 3.99 – 3.98 (m, 2H), 3.97 – 3.94 (m, 3H), 3.94 – 3.93 (m, 1H), 3.91 (dd, *J* = 6.9, 5.8 Hz, 1H), 3.88 – 3.86 (m, 1H), 3.78 – 3.71 (m, 1H), 3.58 (dd, *J* = 9.0, 7.6 Hz, 2H), 3.56 – 3.52 (m, 1H), 3.51 – 3.47 (m, 1H), 3.28 (s, 3H).

^{13}C NMR (151 MHz, $CDCl_3$):- δ 138.9, 138.8, 138.7, 138.0, 128.5, 128.4, 128.4, 128.3, 128.3, 128.2, 128.1, 128.1, 128.0, 128.0, 127.9, 127.8, 127.8, 127.7, 127.7, 127.7, 127.6, 127.5, 127.5, 127.4, 98.8, 98.5, 98.2, 79.5, 79.2, 79.1, 77.3, 77.1, 76.9, 76.5, 76.2, 74.9, 74.7, 73.7, 73.6, 73.6, 73.5, 73.2, 72.8, 72.6, 69.4, 69.1, 68.7, 66.8, 55.4.

OR: $[\alpha]_D^{22.5}$ = -61.4 (C =0.070 g/100 mL in $CHCl_3$)

ESI-MS calcd. for $C_{89}H_{94}O_{16}^+$ [M+K]$^+$ is 1458.71, found 1458.80.

Isolated byproducts

2,3,4,6-Tetra-O-benzyl-D-galactopyranose (z)

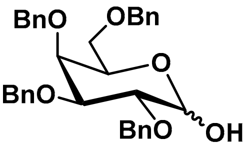

The titled byproduct **z** was isolated from the reaction mixture. It was obtained as a mixture of α and β. Analytical data for **compound z** was in accordance with those reported previously [32].

^{1}H NMR (600 MHz, $CDCl_3$) δ 7.48 – 7.29 (m, 1H), 5.34 (d, *J* = 3.6 Hz, 1H), 5.00 (dd, *J* = 11.5, 3.9 Hz, 1H), 4.89 – 4.82 (m, 1H), 4.81 – 4.74 (m, 1H), 4.71 – 4.61 (m, 1H), 4.53 (dd, *J* = 11.9, 7.8 Hz, 1H), 4.44 (dd, *J* = 11.9, 6.7 Hz, 1H), 4.23 (t, *J* = 6.4 Hz, 1H), 4.18 (q, *J* = 7.1 Hz, 1H), 4.09 (dd, *J* = 9.9, 3.6 Hz, 1H), 4.02 – 3.96 (m, 1H), 3.91 (d, *J* = 2.6 Hz, 1H), 3.85 (dd, *J* = 9.6, 7.6 Hz, 1H), 3.68 – 3.59 (m, 1H), 3.58 – 3.53 (m, 1H), 3.50 (dd, *J* = 9.4, 6.2 Hz, 1H).

^{13}C NMR (151 MHz, $CDCl_3$) δ 138.75, 138.59, 138.53, 138.49, 138.38, 137.83, 137.76, 128.53, 128.49, 128.46, 128.41, 128.39, 128.34, 128.31, 128.29, 128.23, 128.06, 127.89, 127.83, 127.76, 127.71, 127.68, 127.67, 127.62, 127.58, 97.83, 91.84, 82.23, 80.77, 78.78, 77.41, 77.20, 76.99, 76.58, 75.14, 74.88, 74.69, 74.59, 73.66, 73.54, 73.48, 73.38, 73.04, 73.00, 69.40, 69.26, 68.97, 60.51, 53.54.

2,3,4,6-tetra-*O*-benzyl-D-galactal (y)

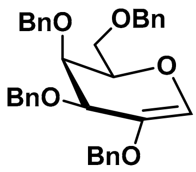

The titled byproduct **y** was isolated from the reaction mixture. Analytical data was in accordance with the literature [33].

^{1}H NMR (600 MHz, $CDCl_3$) δ 7.40 – 7.29 (m, 20H), 6.20 (s, 1H), 4.85 (d, *J* = 12.0 Hz, 1H), 4.80 (dd, *J* = 12.0, 8.4 Hz, 2H), 4.74 (d, *J* = 11.3 Hz, 1H), 4.66 – 4.61 (m, 2H), 4.56 (d, *J* = 11.9 Hz, 1H), 4.46 (d, *J* = 11.9 Hz, 1H), 4.27 (d, *J* = 4.2 Hz, 1H), 4.23 (dd, *J* = 7.6, 3.8 Hz, 1H), 3.96 – 3.90 (m, 2H), 3.71 (dd, *J* = 10.8, 3.4 Hz, 1H).

2,4-Dinitrophenol (x)

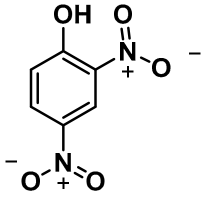

The titled byproduct **x** was isolated from the water layer. 1H NMR data for **compound x** was in accordance with those reported previously [34].

^{1}H NMR (600 MHz, $CDCl_3$) δ 11.06 (s, 1H), 9.11 (d, *J* = 2.7 Hz, 1H), 8.49 (dd, *J* = 9.2, 2.7 Hz, 1H), 7.36 (d, *J* = 9.2 Hz, 1H).

Reaction conditions and results of the microwave reactions

All experiments were carried out following the procedure of general method for microwave assisted glycosylation.

Control experiments were conducted to investigate the reaction time on the stereoselective outcome of the reaction. There was no effect on stereoselectivity due to the reaction time within the given temperature range.

To check the reversibility of the product, product **4*α*** and **4*β*** were used as the substrate in the presence of 2,4 -dinitrophenol and performed a microwave reaction under the general microwave conditions. The product didn't react, and no reversibility was detected.

(2,3,4,6-tetra-*O*-benzyl-D-galactopyranosyl) -(1à6)-1,2:3,4-di-*O*-isopropylidene-*α*-D-galactopyranoside (13)

Entry	Donor (eq)	Donor mmol	Acceptor (eq)	Acceptor mmol	Solvent	Time (h)	Temp.	Yield	*α*:*β*
1[a]	1*α* 1	0.142	1.5	0.212	DMF	1	200	45	3.2:1
2[a]	1β 1	0.142	1.5	0.212	DMF	1	200	48	2:1
3[a,b]	1*α* 1	0.142	1.5	0.212	DCM/Dim.	1.5	150	-	-
4[a,b]	1β 1	0.142	1.5	0.212	DCM/Dim.	1.5	150	-	-
5[a]	1*α* 1.2	0.142	1	0.118	DMF	1	200	68	3:1
6[a]	1β 1.2	0.142	1	0.118	DMF	1	200	60	2.1:1

[a] 2 mL of DMF, [b]10 eq of Dim (dimethylamine)

Methyl (2,3,4,6-tetra-*O*-benzyl-D-galactopyranosyl)-(1à2)-2,4,6-tri-*O*-benzyl-D-glucoopyranoside (14)

Entry	Donor (eq)	Donor mmol	Acceptor (eq)	Acceptor mmol	Solvent	Time (h)	Temp.	Yield	*α:β*
1[a]	1α 1	0.142	1.5	0.212	DMF	1	200	40	α only
2[a]	1β 1	0.142	1.5	0.212	DMF	1	200	55	3:1
3[a,b]	1α 1	0.142	1.5	0.212	DCM/Dim.	1.5	150	20	α only
4[a,b]	1β 1	0.142	1.5	0.212	DCM/Dim.	1.5	150	23	3.5:1
5[a]	1α 1.2	0.142	1	0.118	DMF	1	200	65	α only
6[a]	1β 1.2	0.142	1	0.118	DMF	1	200	58	3:1

[a] 2 mL of DMF, [b]10 eq of Dim (dimethylamine)

Methyl (2,3,4,6-tetra-*O*-benzyl-D-galactopyranosyl) -(1à3)-2,4,6-tri-*O*-benzyl-D-glucoopyranoside (15)

Entry	Donor (eq)	Donor mmol	Acceptor (eq)	Acceptor mmol	Solvent	Time (h)	Temp.	Yield	*α:β*
1[a]	1α 1	0.142	1.5	0.212	DMF	1	200	50	3:1
2[a]	1β 1	0.142	1.5	0.212	DMF	1	200	58	2.1:1
3[a,b]	1α 1	0.142	1.5	0.212	DCM/Dim.	1.5	150	30	3:1
4[a,b]	1β 1	0.142	1.5	0.212	DCM/Dim.	1.5	150	25	2:1
5[a]	1α 1.2	0.142	1	0.118	DMF	1	200	62	3.5:1
6[a]	1β 1.2	0.142	1	0.118	DMF	1	200	65	2:1

[a] 2 mL of DMF, [b]10 eq of Dim (dimethylamine)

Cholesteryl 2,3,4,6-tetra-*O*-benzyl-D-glucopyranoside (18)

Entry	Donor (eq)	Donor mmol	Acceptor (eq)	Acceptor mmol	Solvent	Time (h)	Temp.	Yield	*α:β*
1 [a,b]	1α 1	0.142	1.5	0.212	DCM/Dim	1	150	50	3:1
2 [a,b]	1β 1	0.142	1.5	0.212	DCM/Dim	1	150	57	3.1:1
3 [a]	1α 1.2	0.142	1	0.212	DMF	1.5	150	60	3.1:1
4[a]	1β 1.2	0.142	1	0.212	DMF	1.5	150	58	3.2:1
5[a]	1α 1.2	0.142	1	0.118	DMF	1	200	80	2.5:1
6[a]	1β 1.2	0.142	1	0.118	DMF	1	200	75	3:1

1-Adamantanyl 2,3,4,6-tetra-*O*-benzyl-D-glucopyranoside (17)

Entry	Donor (eq)	Donor mmol	Acceptor (eq)	Acceptor mmol	Solvent	Time (h)	Temp.	Yield	*α:β*
1 [a,b]	1α 1	0.142	1.5	0.212	DCM/Dim	1	150	60	α only
2 [a,b]	1β 1	0.142	1.5	0.212	DCM/Dim	1	150	50	α only
3 [a]	1α 1.2	0.142	1	0.212	DMF	1.5	150	65	α only
4[a]	1β 1.2	0.142	1	0.212	DMF	1.5	150	70	α only
5[a]	1α 1.2	0.142	1	0.118	DMF	1	200	60	α only
6[a]	1β 1.2	0.142	1	0.118	DMF	1	200	68	α only

Spectroscopic data

2,3,4,β-tetra-*O*-benzyl-α-D-galactopyranosyl phenylacetate (1)

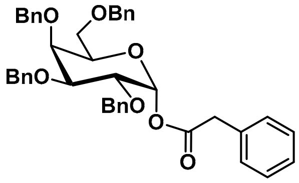

^{1}H NMR (600 MHz, $CDCl_3$)

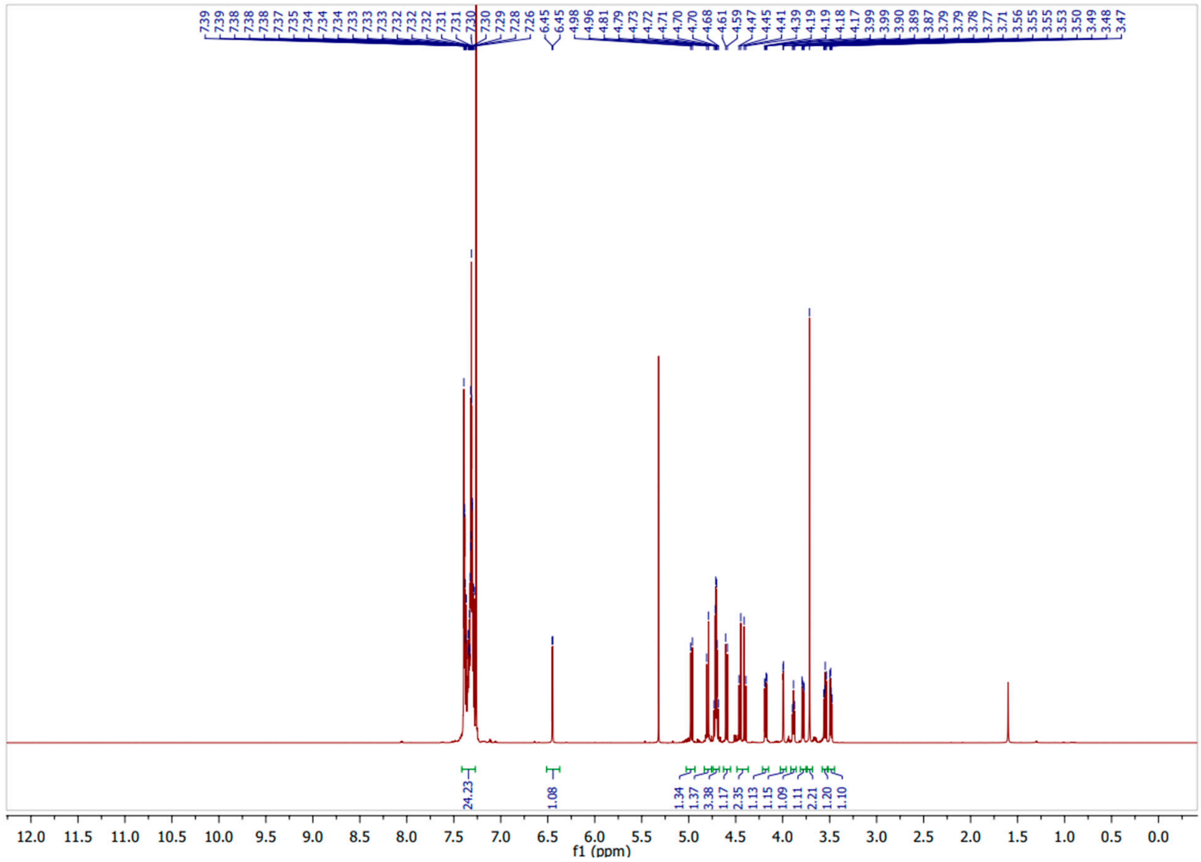

2,3,4,6-tetra-*O*-benzyl-α-D-galactopyranosyl phenylacetate (1)

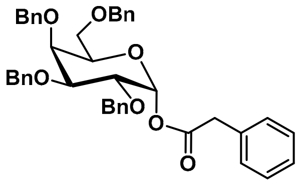

^{13}C NMR (151 MHz, $CDCl_3$)

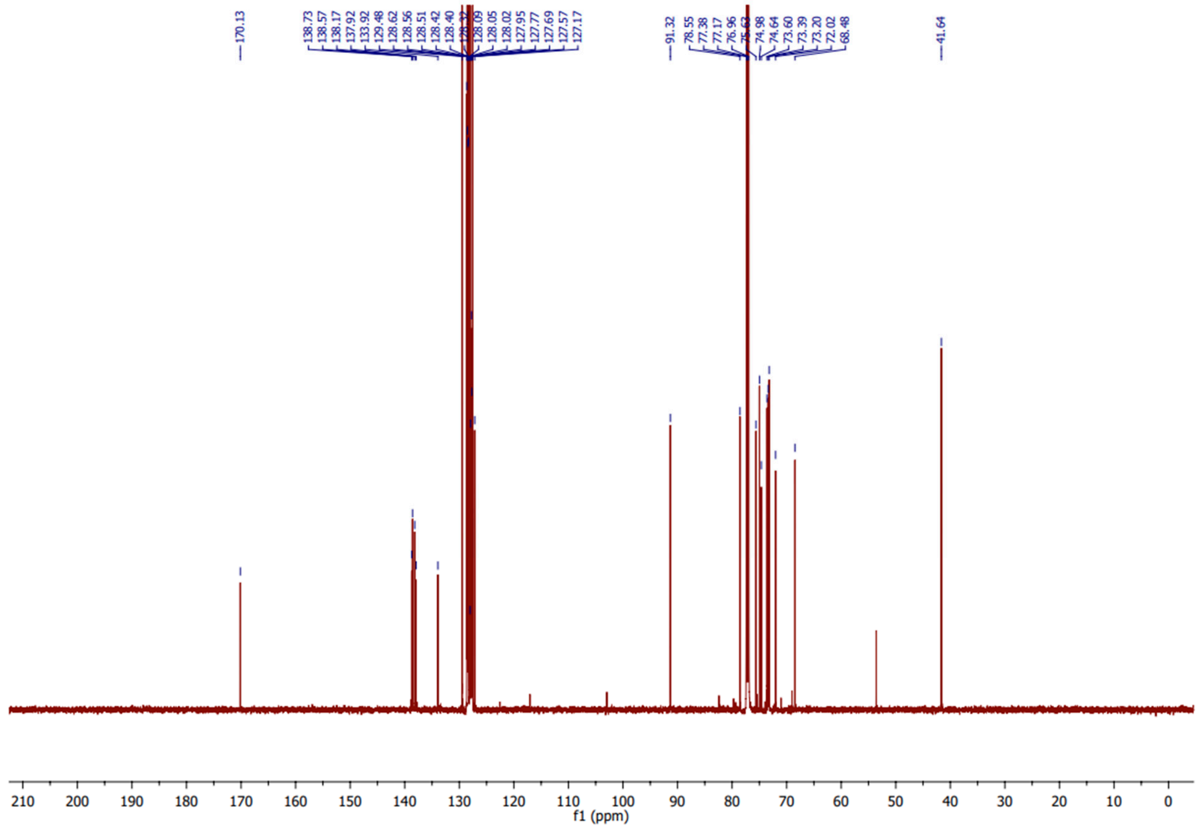

2-Methoxycarbonyl-4-nitrophenyl 2,3,4,6-tetra-O-benzyl-D-galactopyranoside (2)

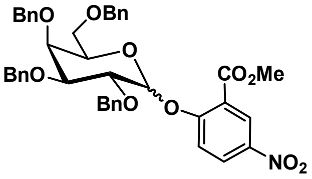

^{1}H NMR (600 MHz, $CDCl_3$)

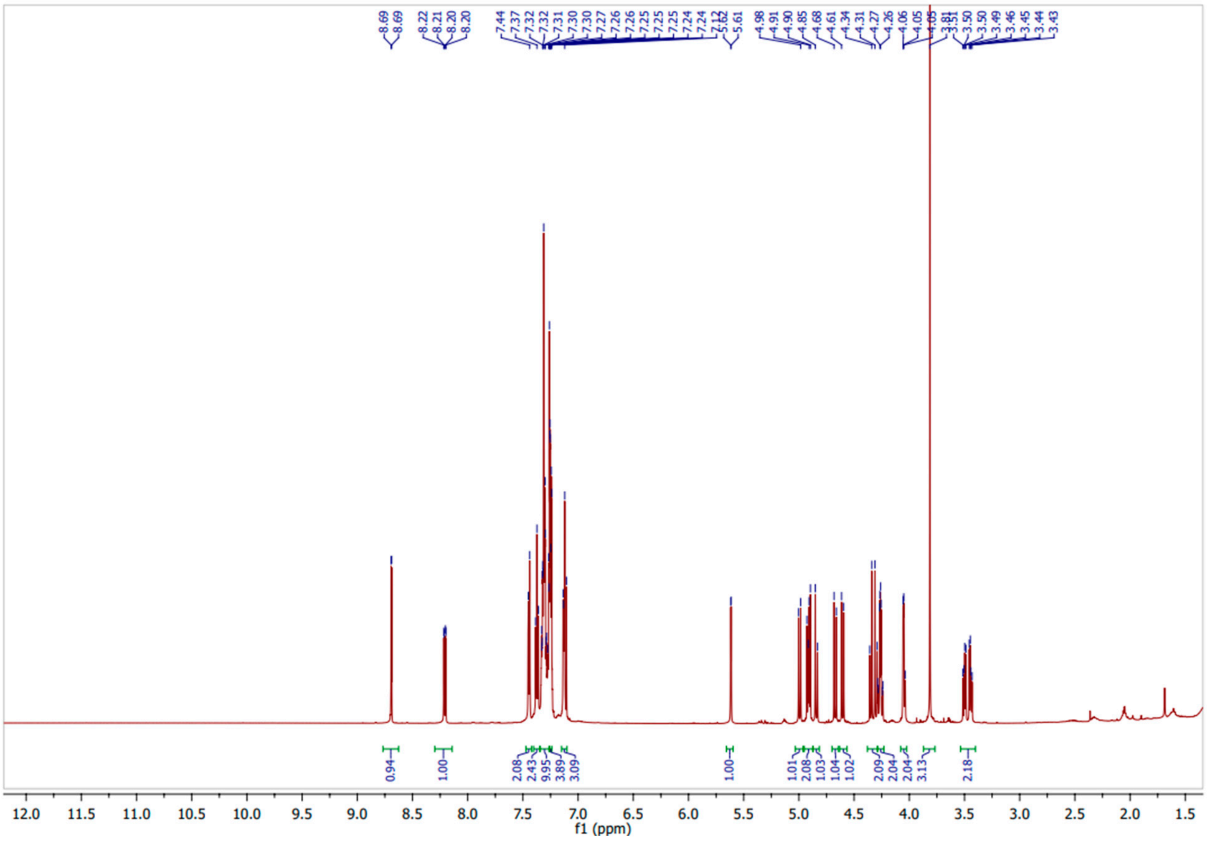

2-Methoxycarbonyl-4-nitrophenyl 2,3,4,6-tetra-O-benzyl-D-galactopyranoside (2)

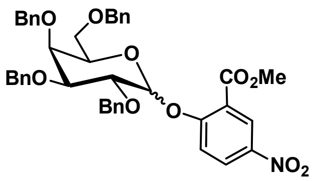

^{13}C NMR (151 MHz, CDCl$_3$)

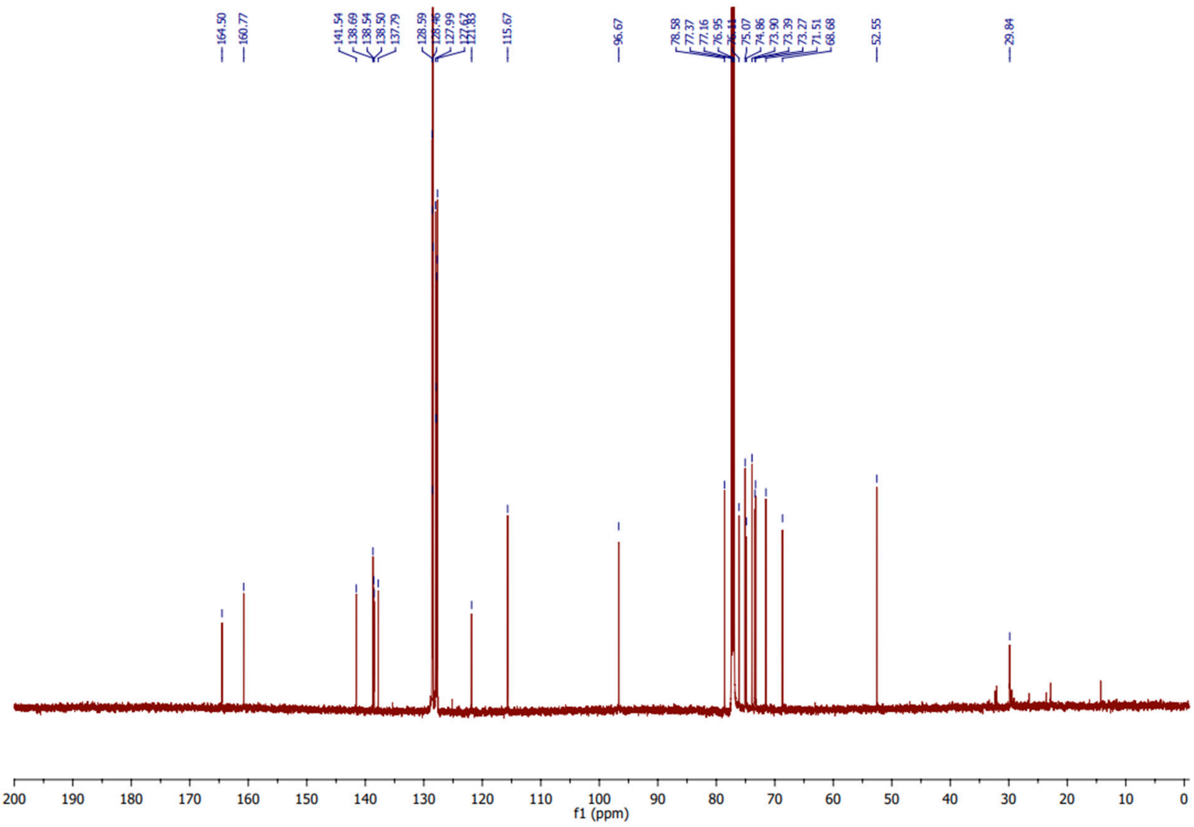

2,4-Dinitrophenyl 2,3,4,6-tetra-*O*-benzyl-D-*α*-galactopyranoside (3a)

^{1}H NMR

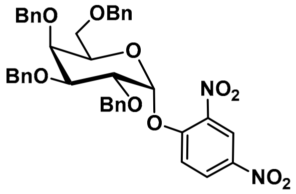

^{1}H NMR (600 MHz, CDCl3)

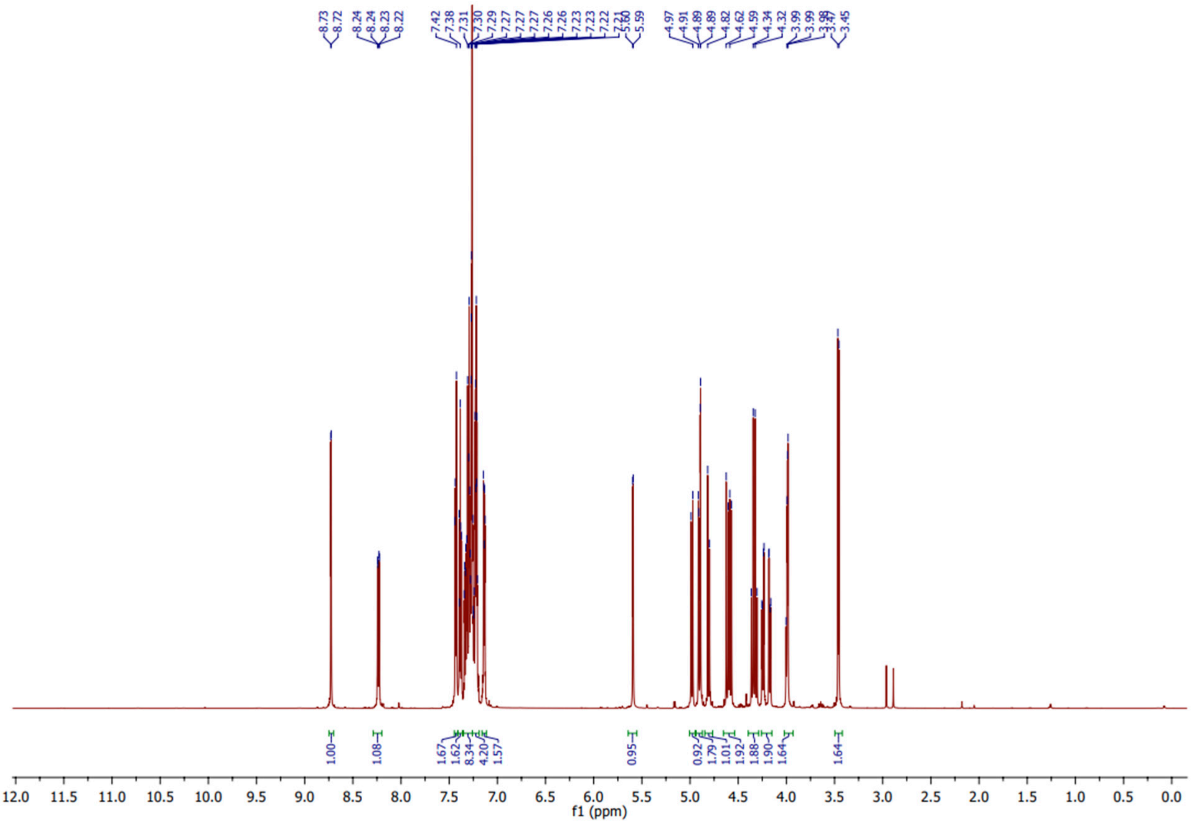

2,4-Dinitrophenyl 2,3,4,6-tetra-*O*-benzyl-D-*α*-galactopyranoside (3a)

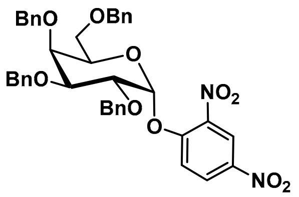

^{13}C NMR (151 MHz, $CDCl_3$)

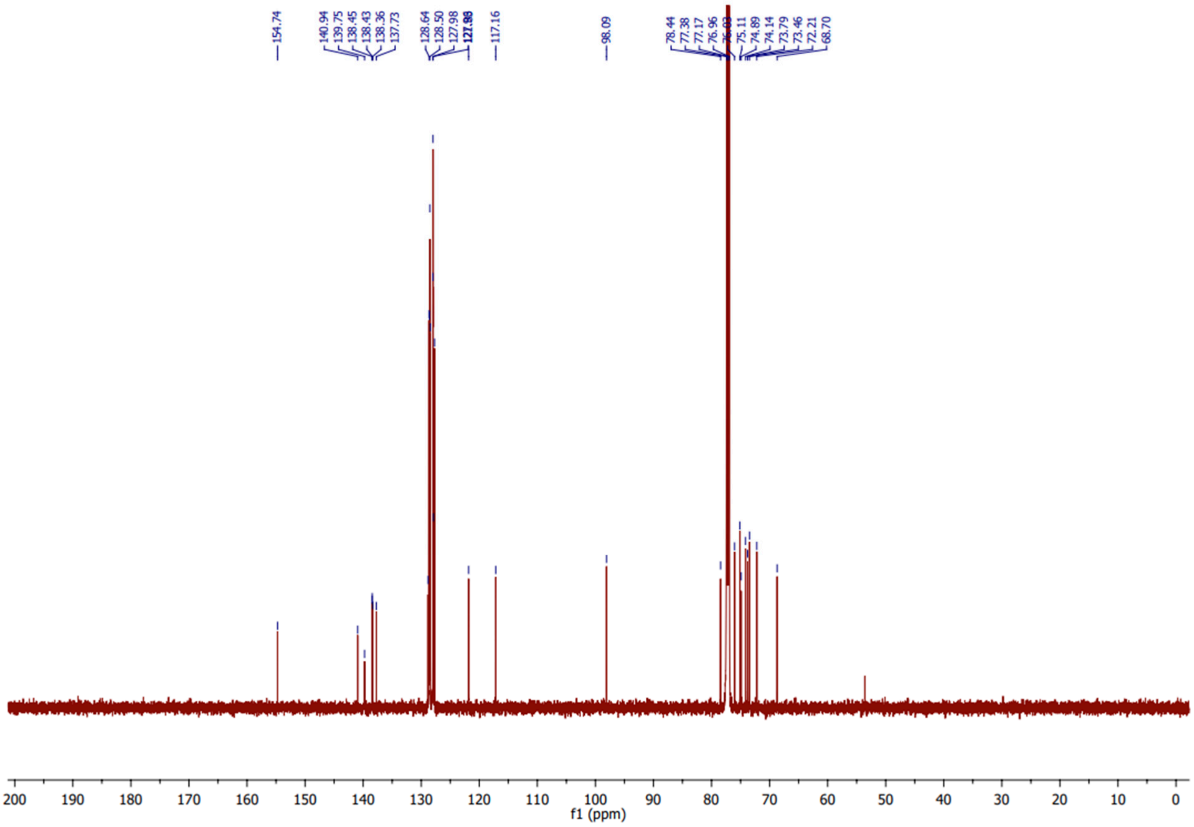

2,4-Dinitrophenyl 2,3,4,6-tetra-*O*-benzyl-D-*β*-galactopyranoside (3b)

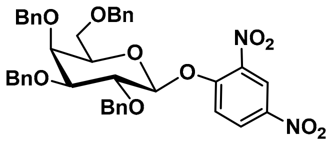

^{1}H NMR (600 MHz, $CDCl_3$)

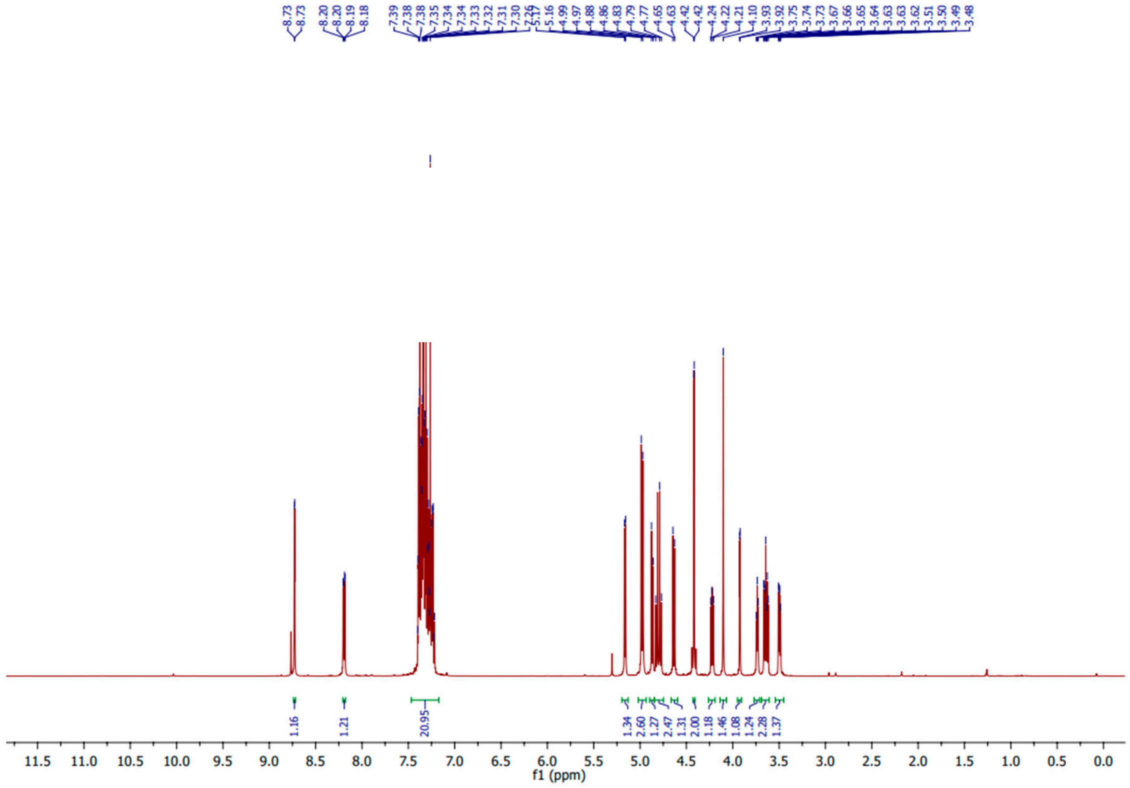

2,4-Dinitrophenyl 2,3,4,6-tetra-*O*-benzyl-D-*β*-galactopyranoside (3b)

^{13}C NMR

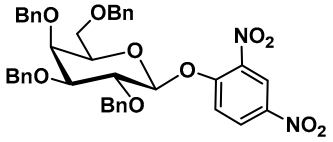

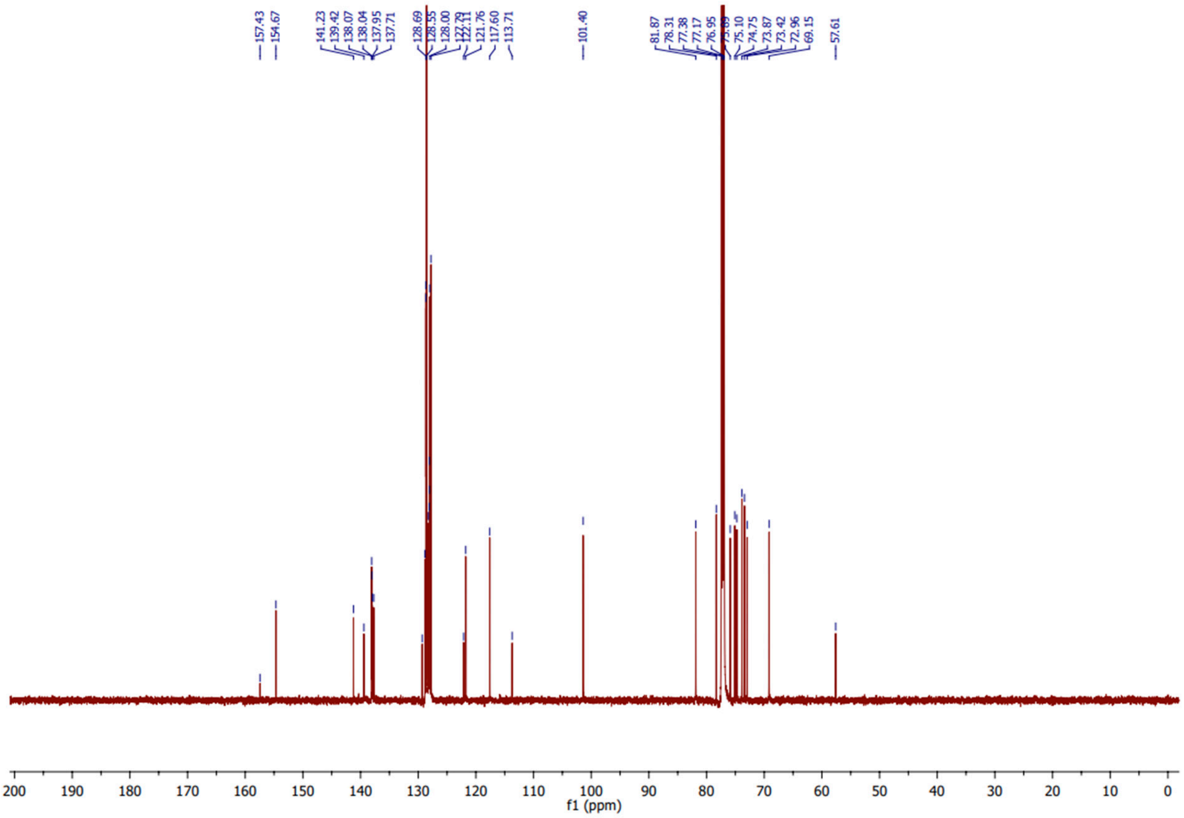

Methyl 2,3,4,6-tetra-*O*-α-D-galactopyranoside (4*α*)

^{1}H NMR

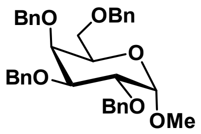

^{1}H NMR (600 MHz, $CDCl_3$)

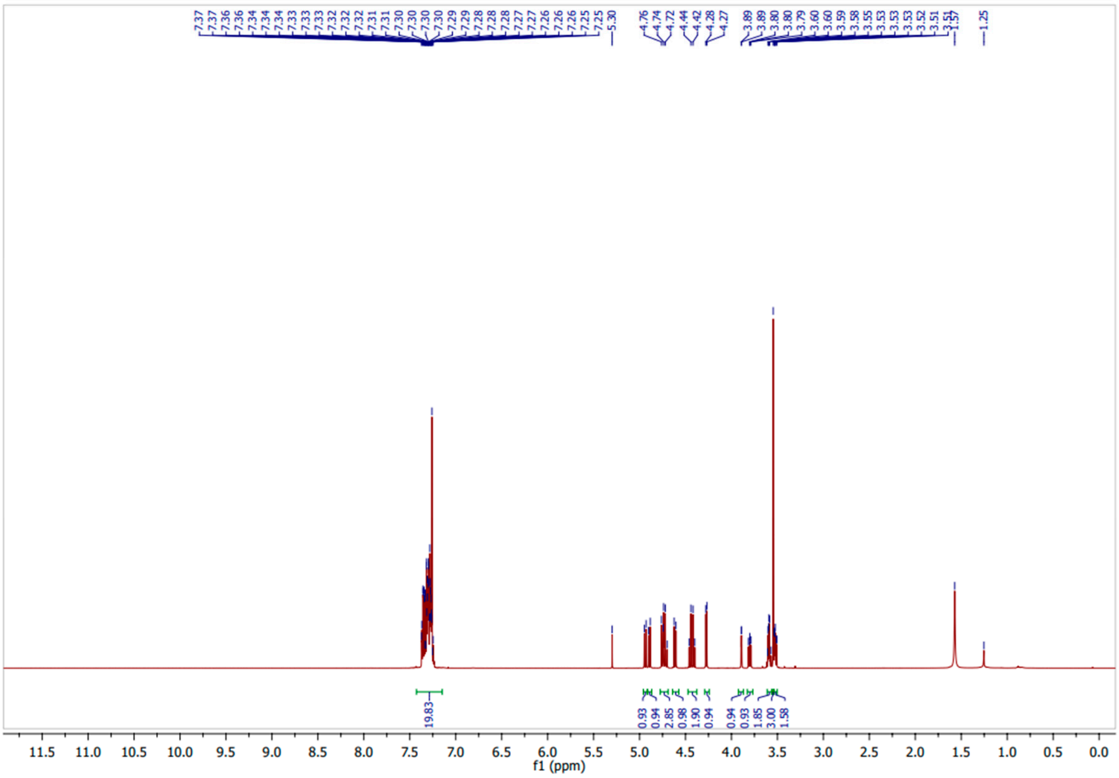

Methyl 2,3,4,6-tetra-*O*-α-D-galactopyranoside (4*α*)

^{13}C NMR

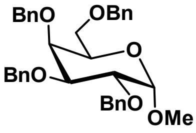

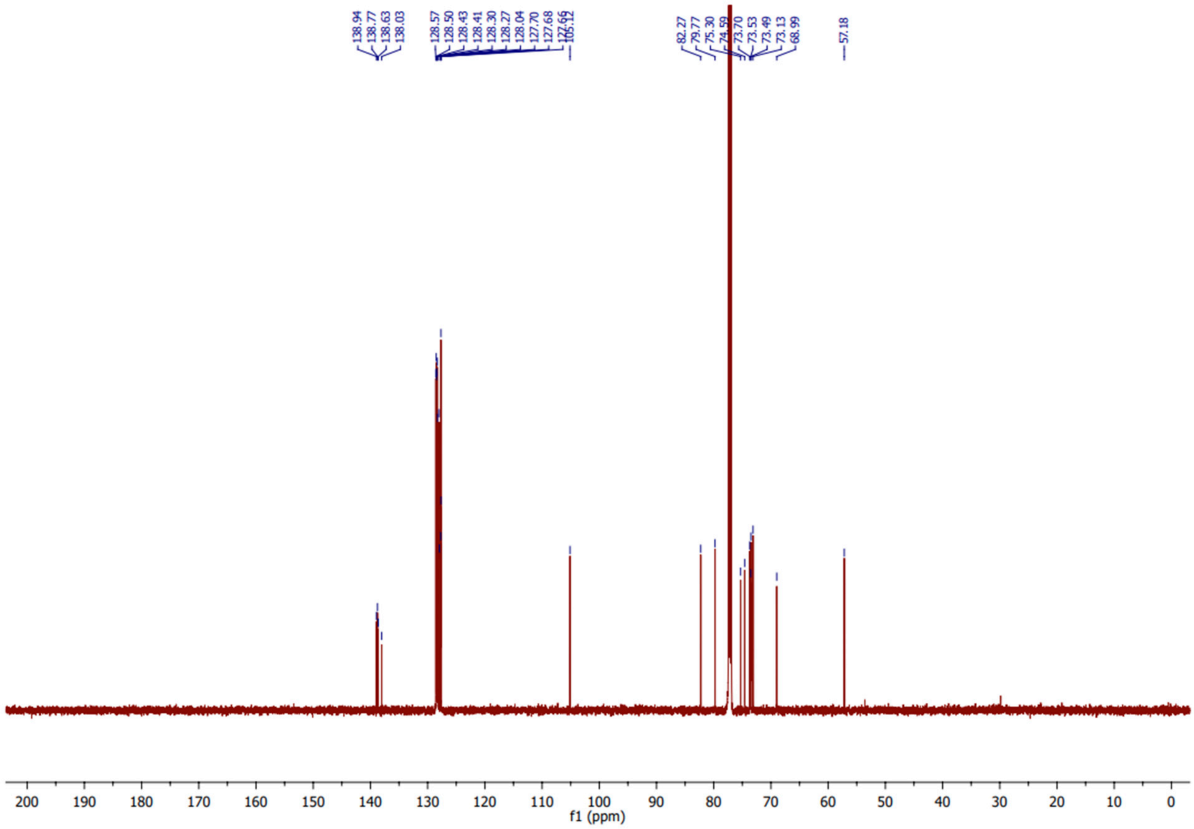

Methyl 2,3,4,6-tetra-*O*-α-D-galactopyranoside (4*β*)

^{1}H NMR

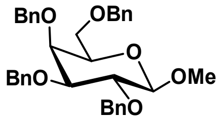

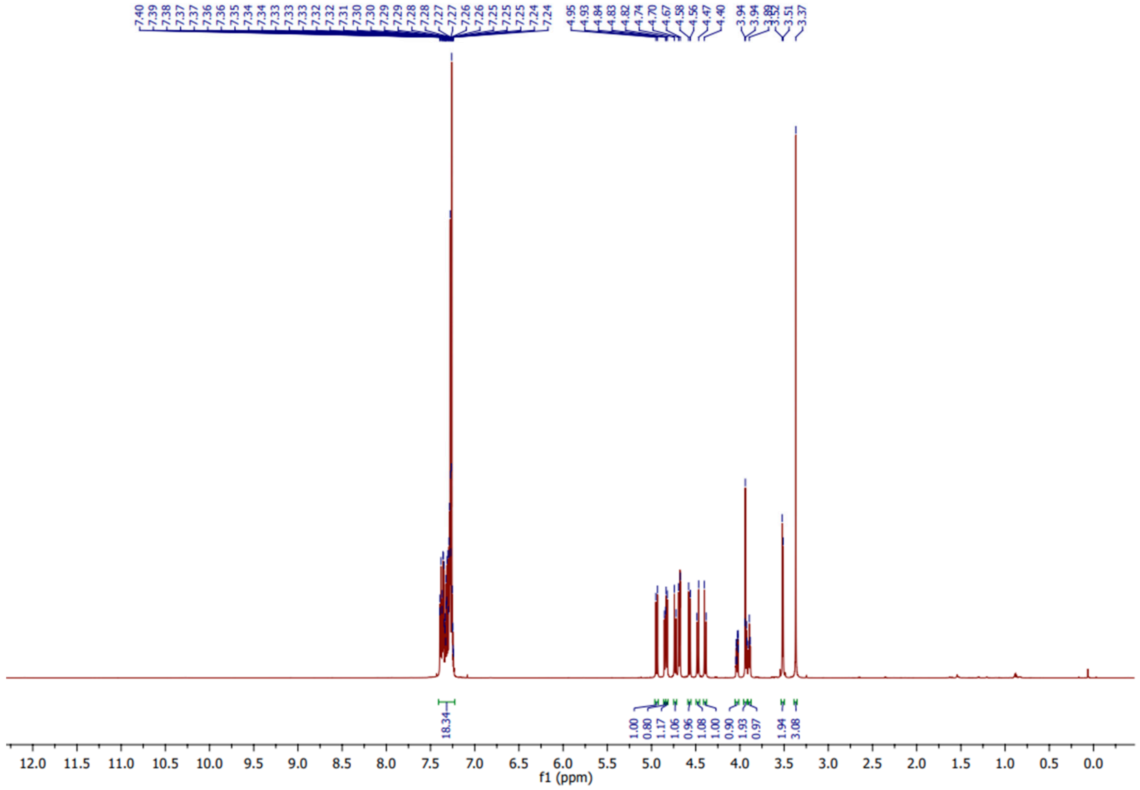

Methyl 2,3,4,6-tetra-*O*-α-D-galactopyranoside (4*β*)

^{13}C NMR

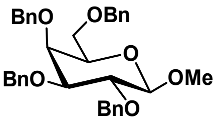

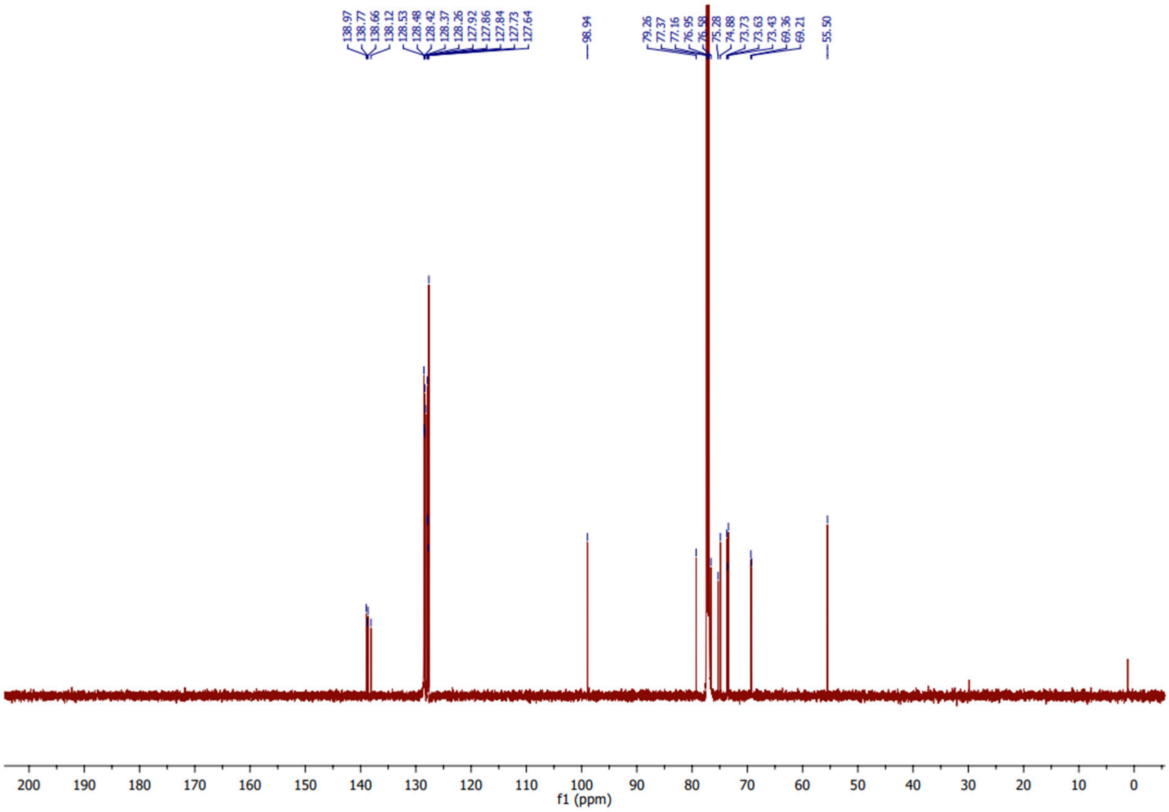

Methyl 2,3,4-tri-*O*-benzyl-*α*-D-galactopyranoside (5)

^{1}H NMR

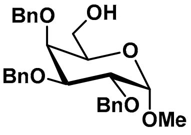

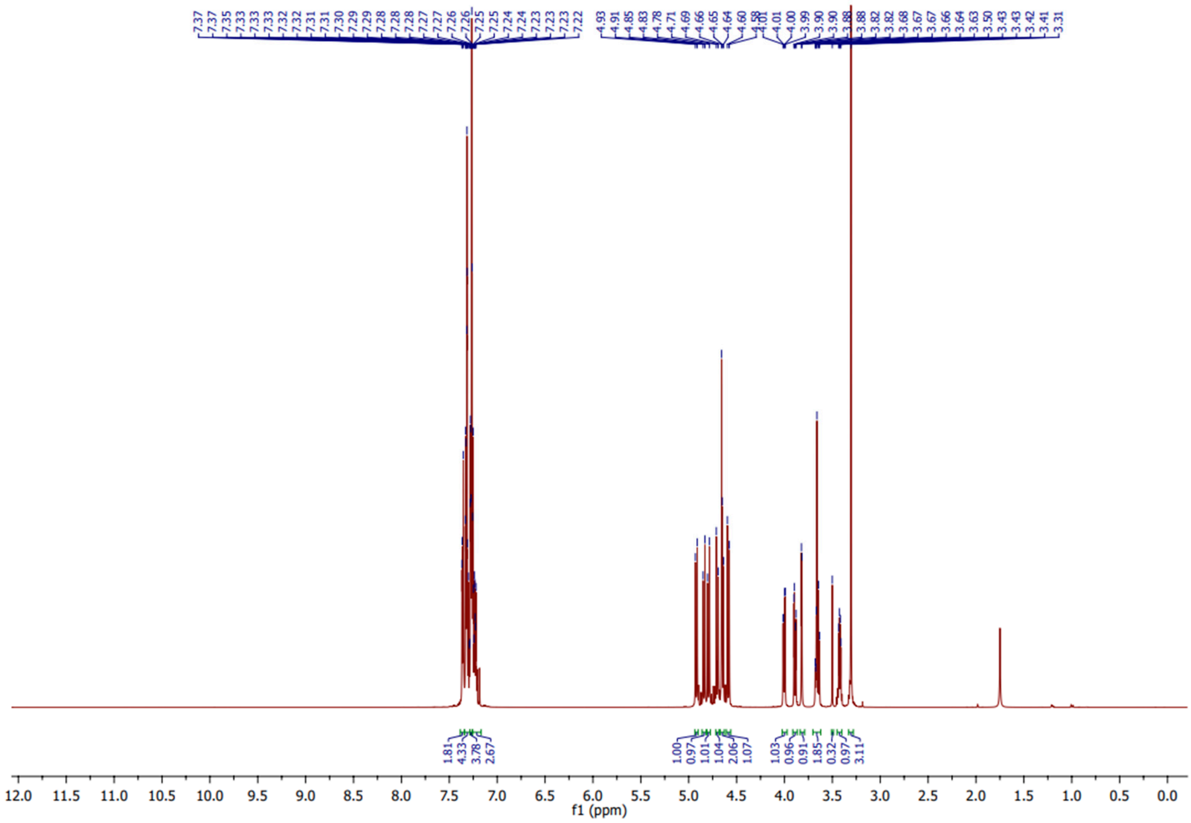

Methyl 2,3,4-tri-*O*-benzyl-*α*-D-galactopyranoside (5)

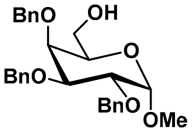

^{13}C NMR (151 MHz, $CDCl_3$)

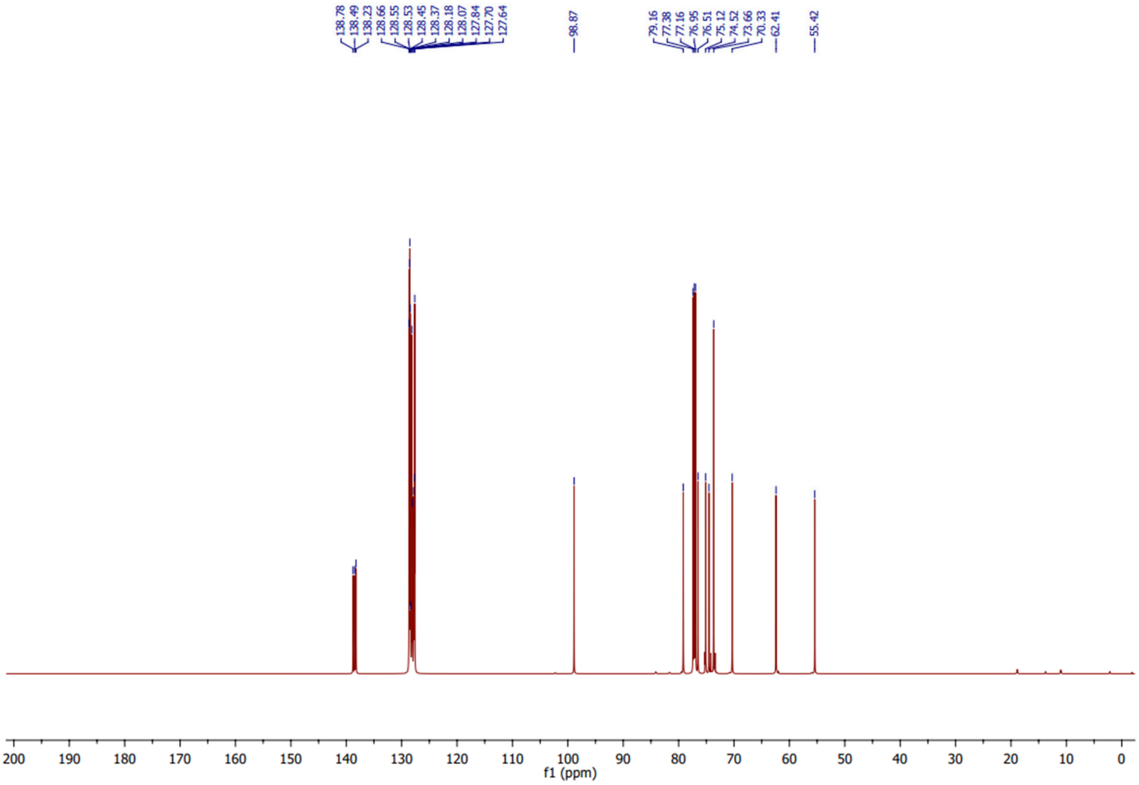

Methyl (2,3,4,6-tetra-*O*-benzyl-*β*-D-galactopyranosyl)-(1à6)-2,3,4-tri-*O*-benzyl-*α*-D-galactopyranoside (6*β*)

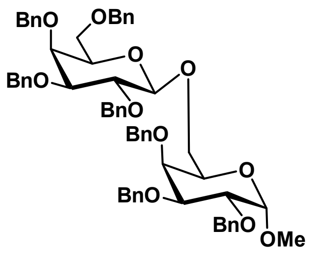

^{1}H NMR (600 MHz, $CDCl_3$)

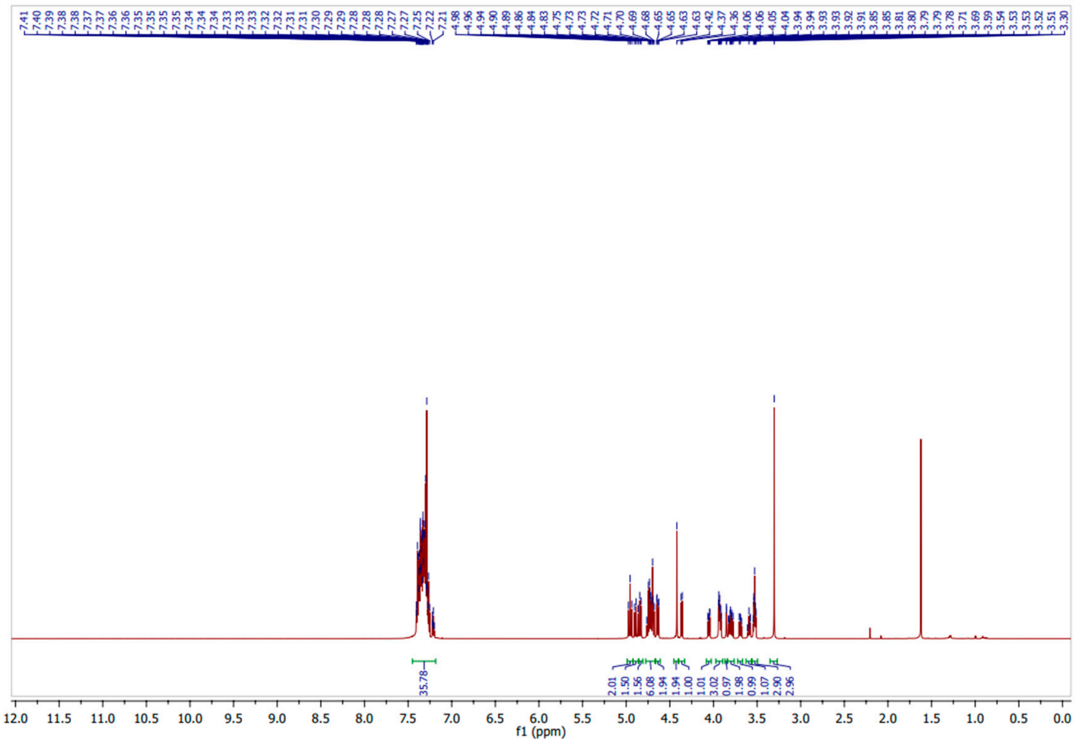

Methyl (2,3,4,6-tetra-*O*-benzyl-*β*-D-galactopyranosyl)-(1à6)-2,3,4-tri-*O*-benzyl-*α*-D-galactopyranoside (6*β*)

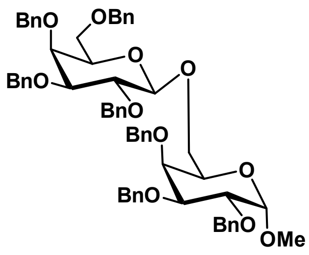

^{13}C NMR (151 MHz, CDCl₃)

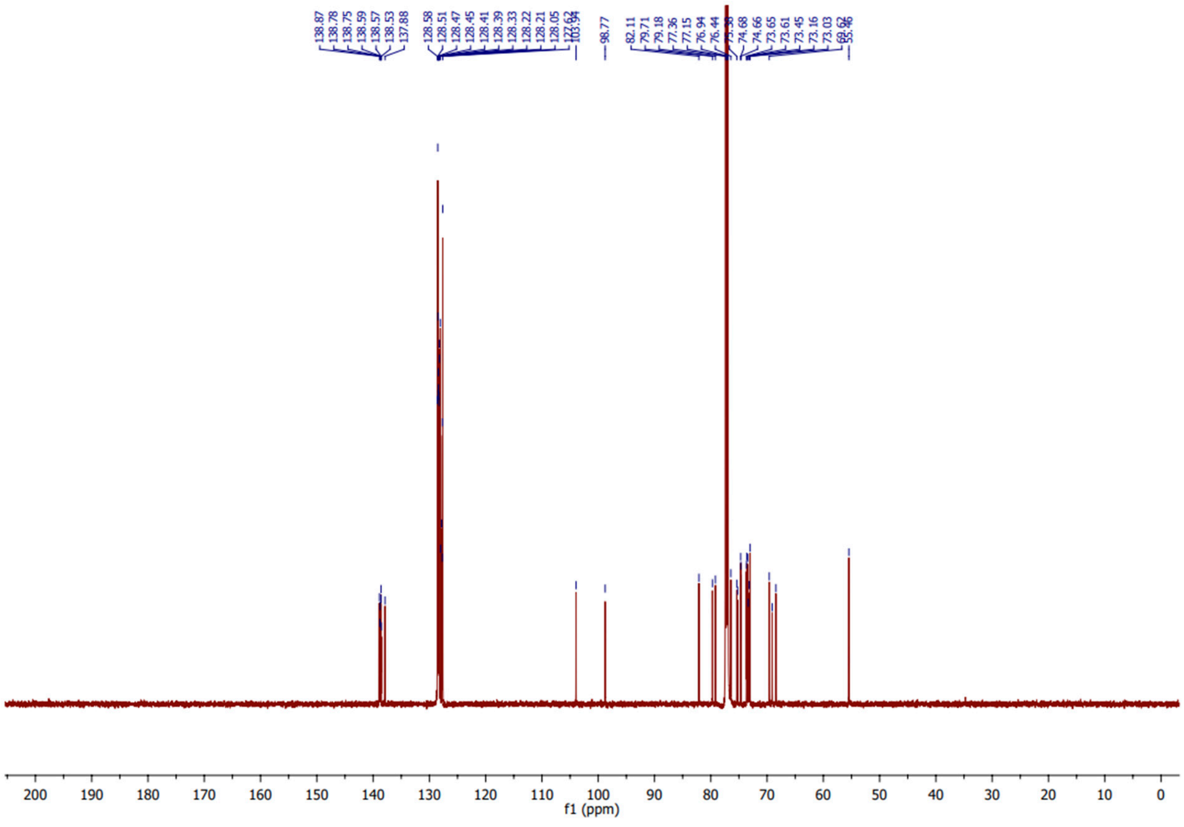

Methyl (2,3,4,6-tetra-*O*-benzyl-*α*-D-galactopyranosyl)-(1à6)-2,3,4-tri-O-benzyl-*α*-D-galactopyranoside (6*α*)

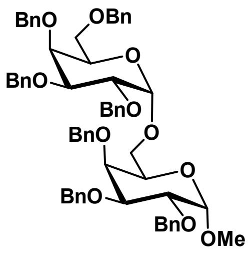

^{1}H NMR (600 MHz, $CDCl_3$)

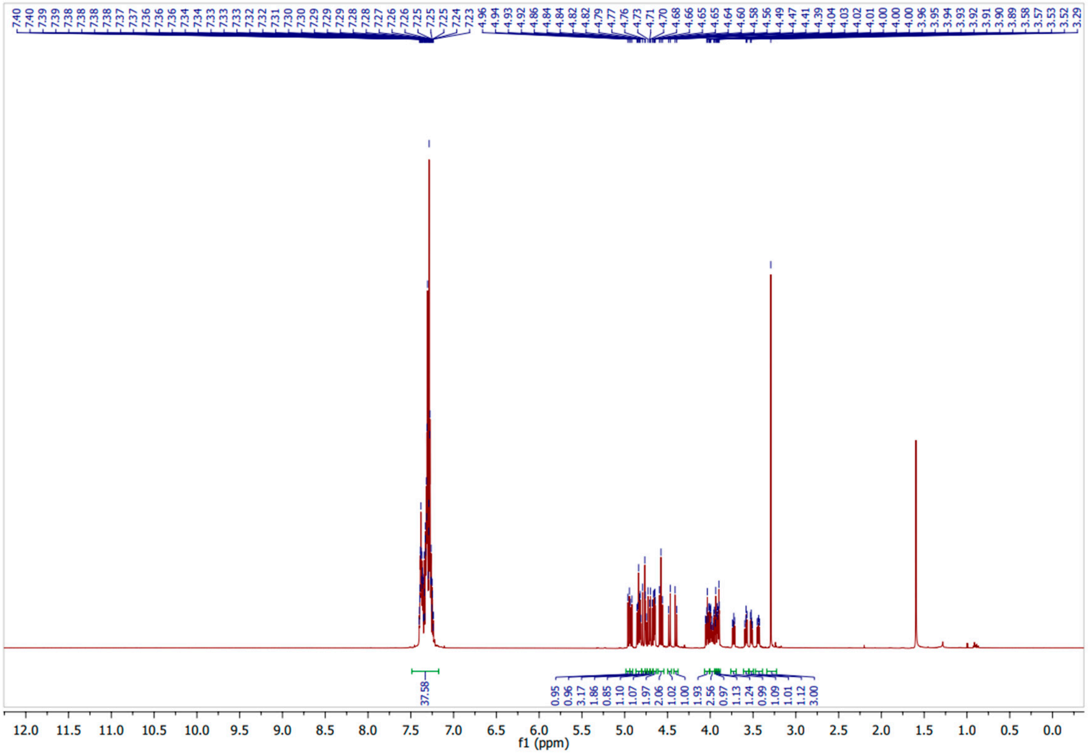

Methyl (2,3,4,6-tetra-*O*-benzyl-*α*-D-galactopyranosyl)-(1à6)-2,3,4-tri-O-benzyl-*α*-D-galactopyranoside (6*α*)

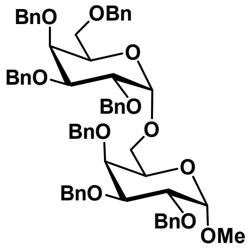

^{13}C NMR (151 MHz, $CDCl_3$)

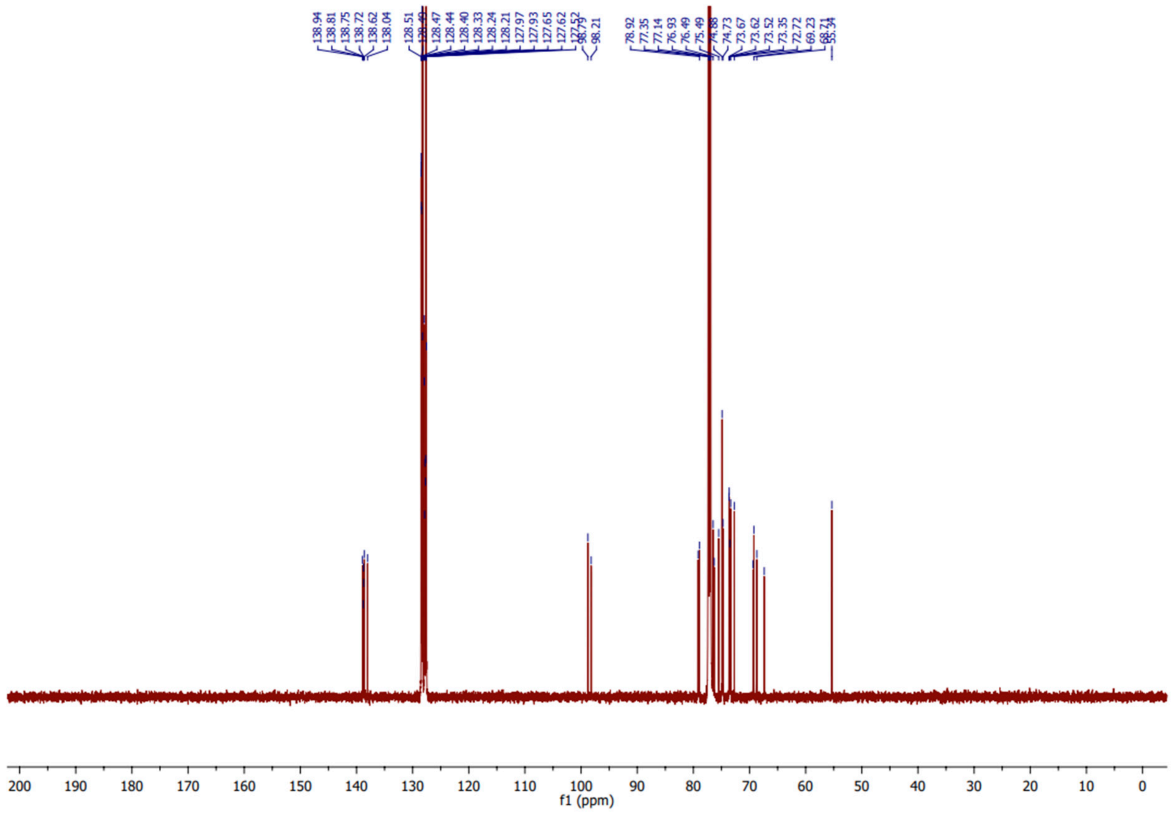

Methyl 3,4,6-tri-*O*-benzyl-*β*-D-glucopyranoside (8)

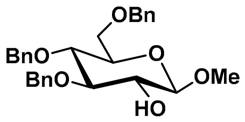

^{1}H NMR (600 MHz, $CDCl_3$)

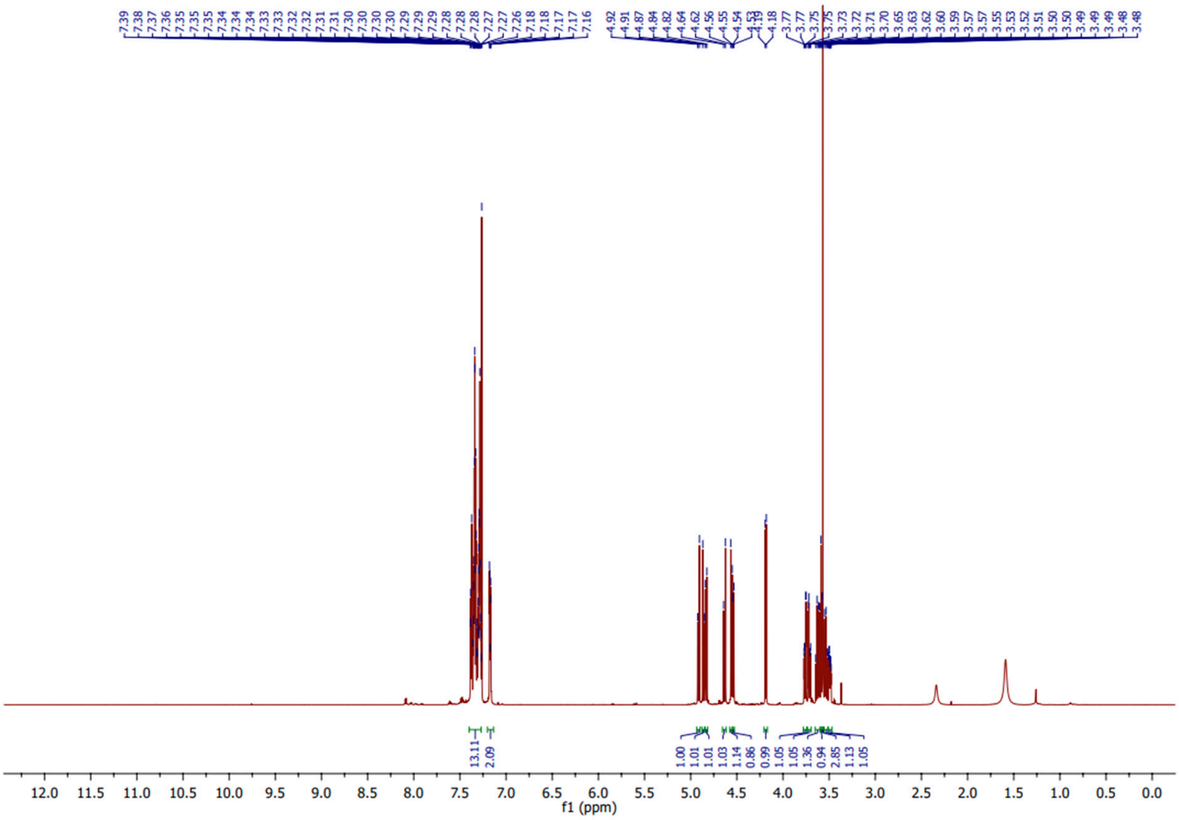

Methyl 2,4,6-tri-*O*-benzyl-*β*-D-glucopyranoside (9)

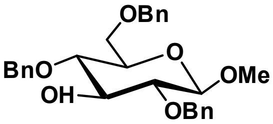

^{1}H NMR (600 MHz, CDCl$_3$)

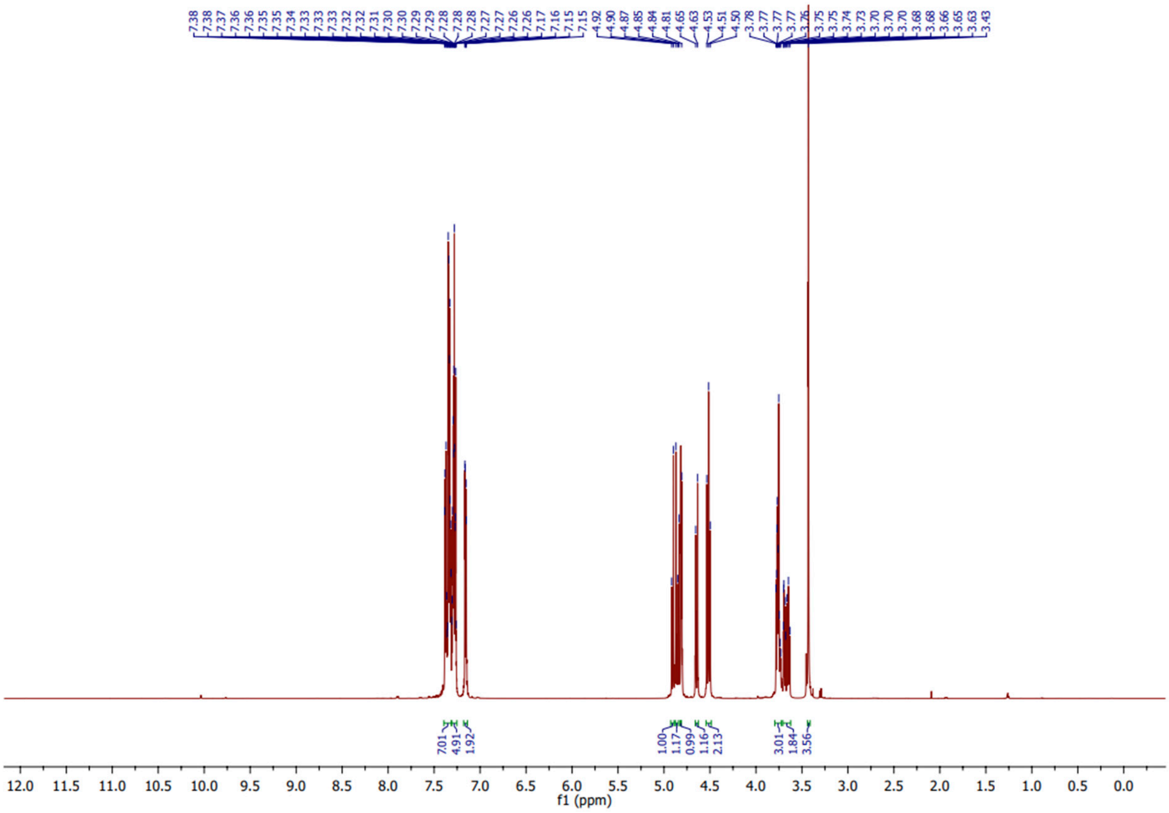

Methyl 2,4,6-tri-*O*-benzyl-*β*-D-glucopyranoside (9)

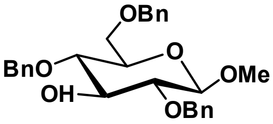

^{13}C NMR (151 MHz, CDCl$_3$)

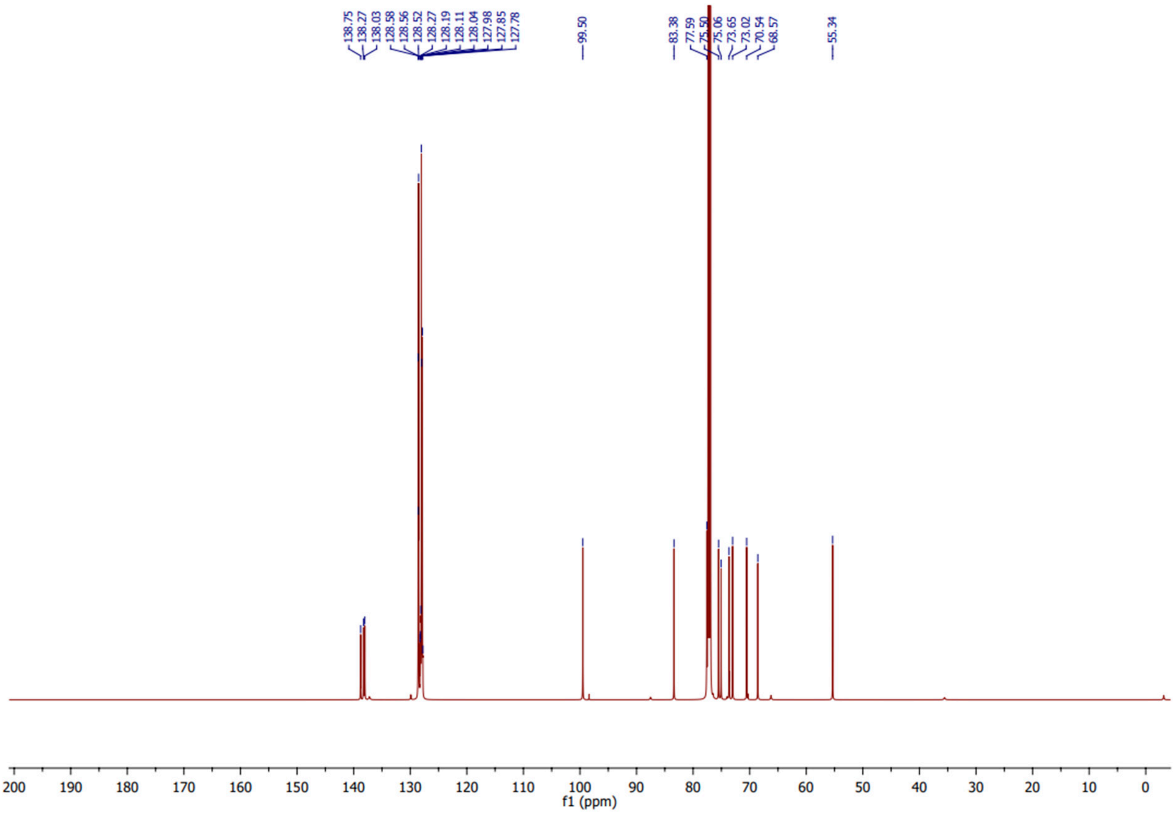

Methyl 2,3,6-tri-*O*-benzyl-*β*-D-glucopyranoside (10)

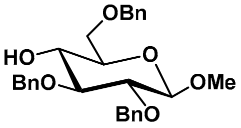

^{1}H NMR (600 MHz, $CDCl_3$)

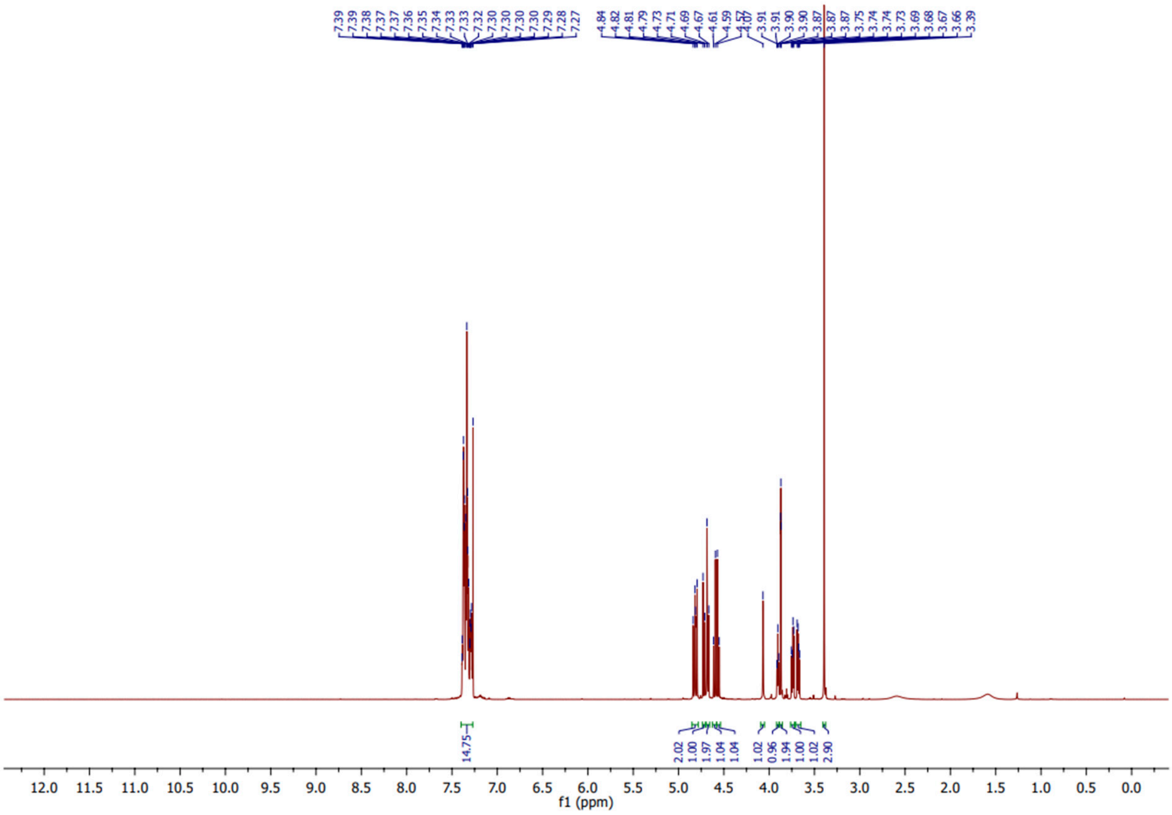

Methyl 2,3,6-tri-*O*-benzyl-*β*-D-glucopyranoside (10)

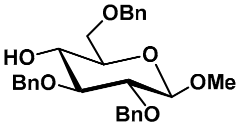

^{13}C NMR (151 MHz, CDCl$_3$)

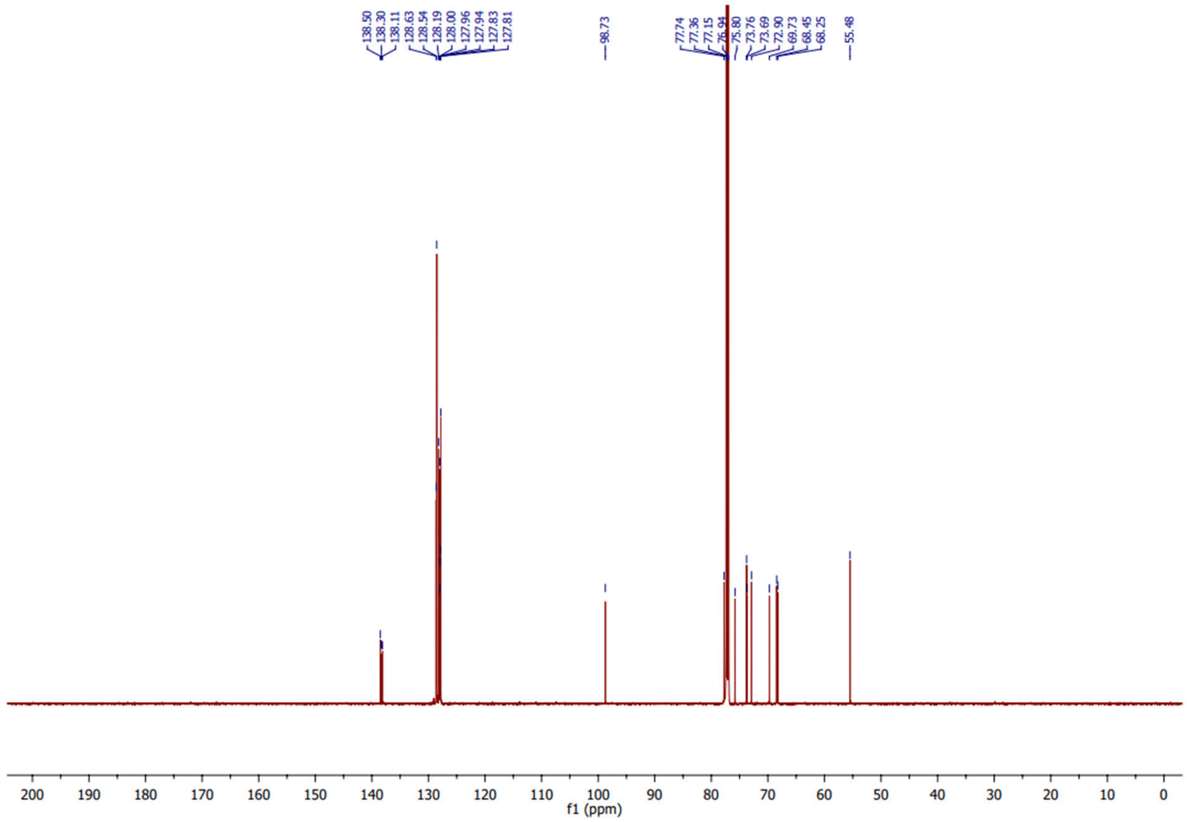

(2,3,4,6-tetra-*O*-benzyl-α-D-galactopyranosyl)-(1à6)-1,2:3,4-di-*O*-isopropylidene-*α*-D-galactopyranoside (13*α*)

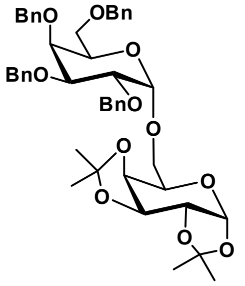

^{1}H NMR (600 MHz, $CDCl_3$)

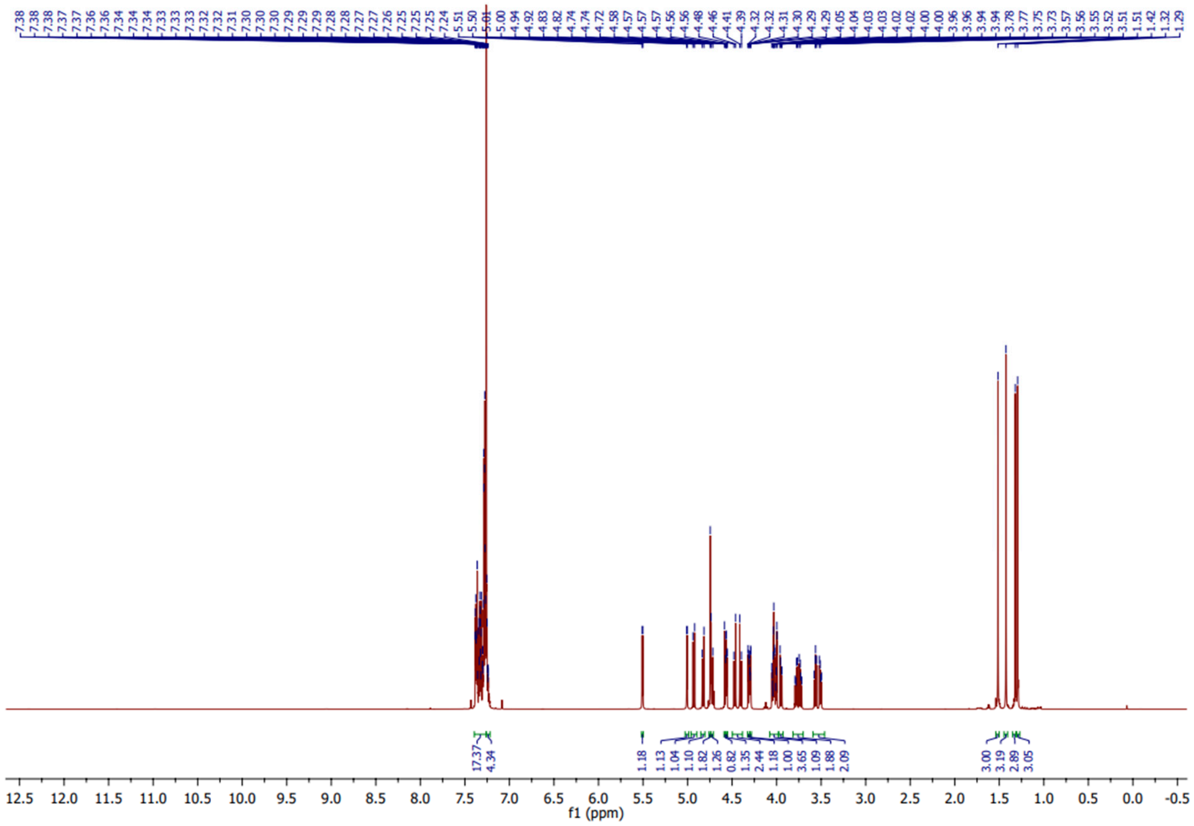

(2,3,4,6-tetra-*O*-benzyl-α-D-galactopyranosyl)-(1à6)-1,2:3,4-di-*O*-isopropylidene-*α*-D-galactopyranoside (13*α*)

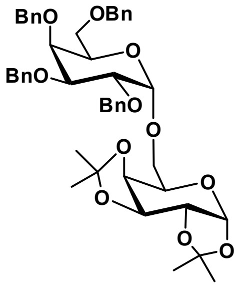

^{13}C NMR (151 MHz, $CDCl_3$)

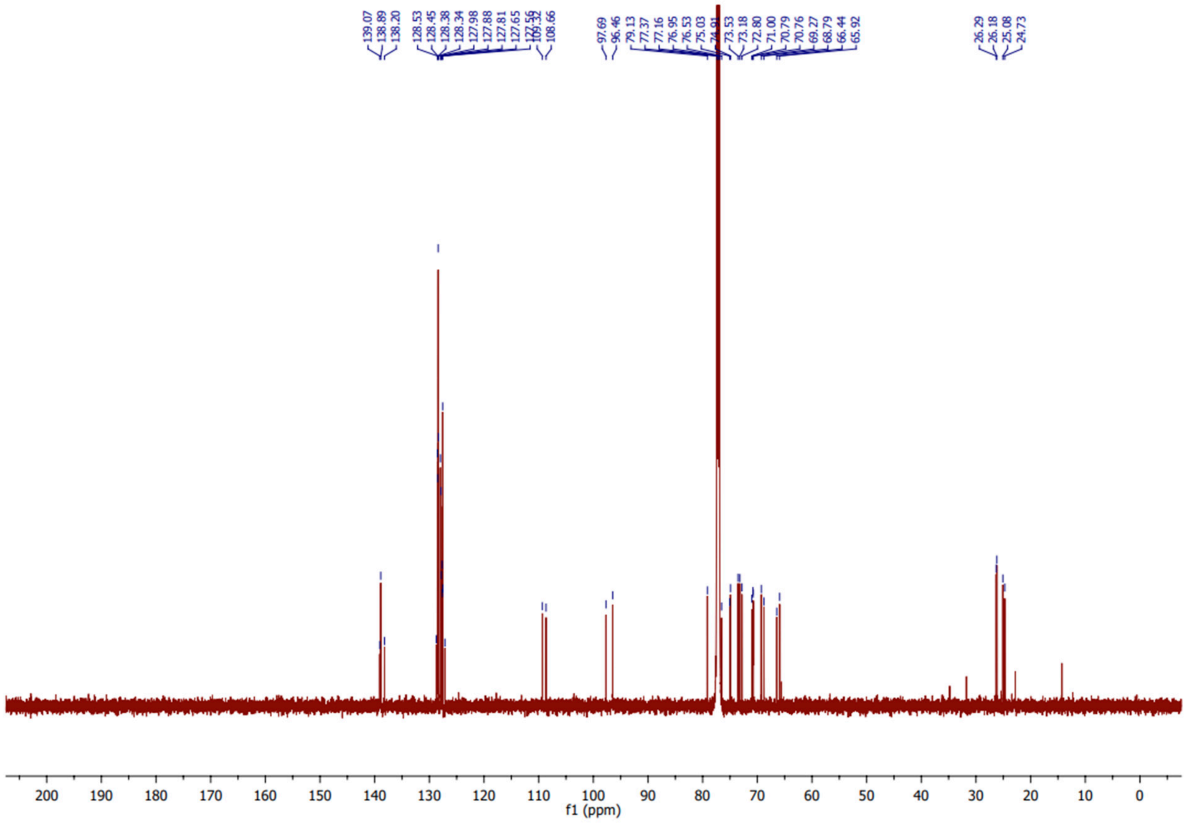

(2,3,4,6-tetra-*O*-benzyl-*β*-D-galactopyranosyl)-(1à6)-1,2:3,4-di-*O*-isopropylidene-*α*-D-galactopyranoside (13*β*)

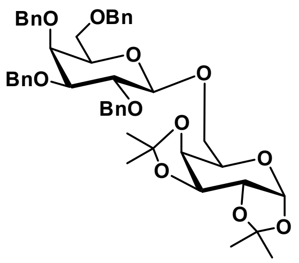

^{1}H NMR (600 MHz, CDCl₃)

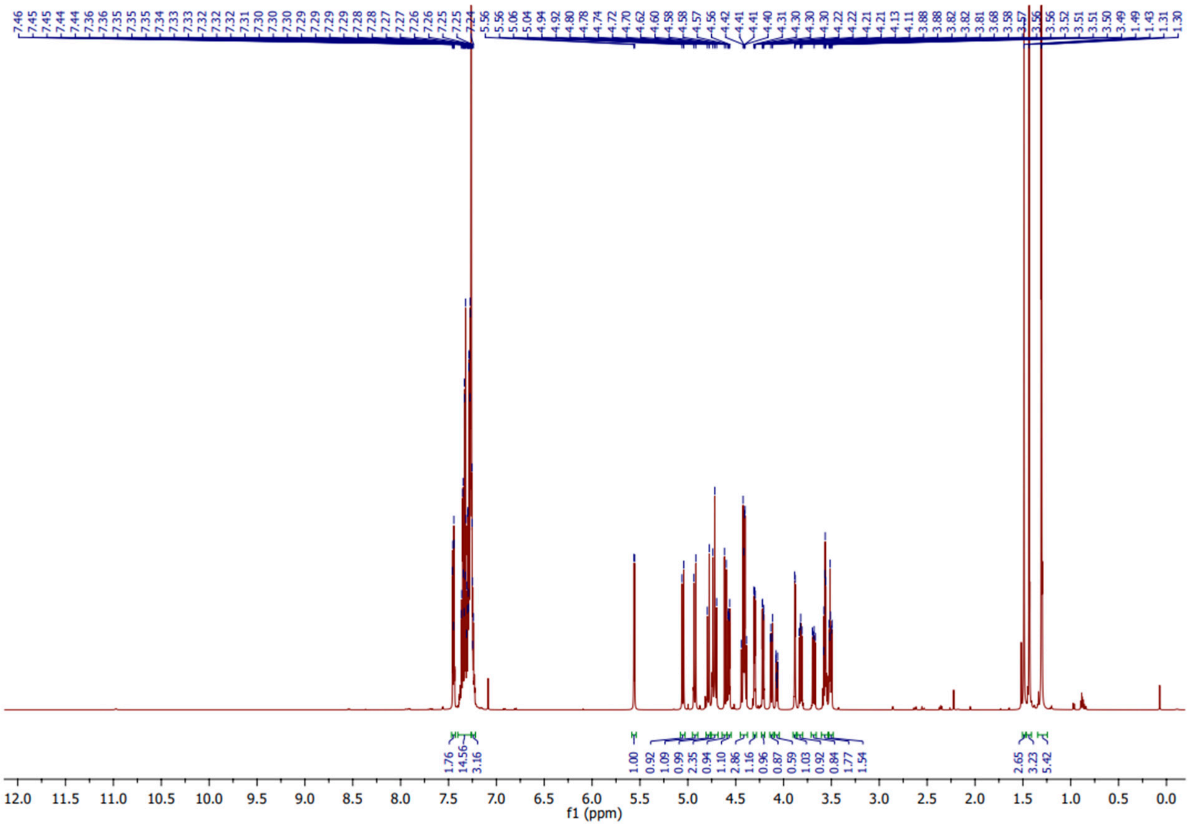

(2,3,4,6-tetra-*O*-benzyl-*β*-D-galactopyranosyl)-(1à6)-1,2:3,4-di-*O*-isopropylidene-*α*-D-galactopyranoside (13*β*)

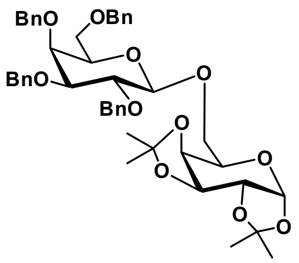

^{13}C NMR (151 MHz, $CDCl_3$)

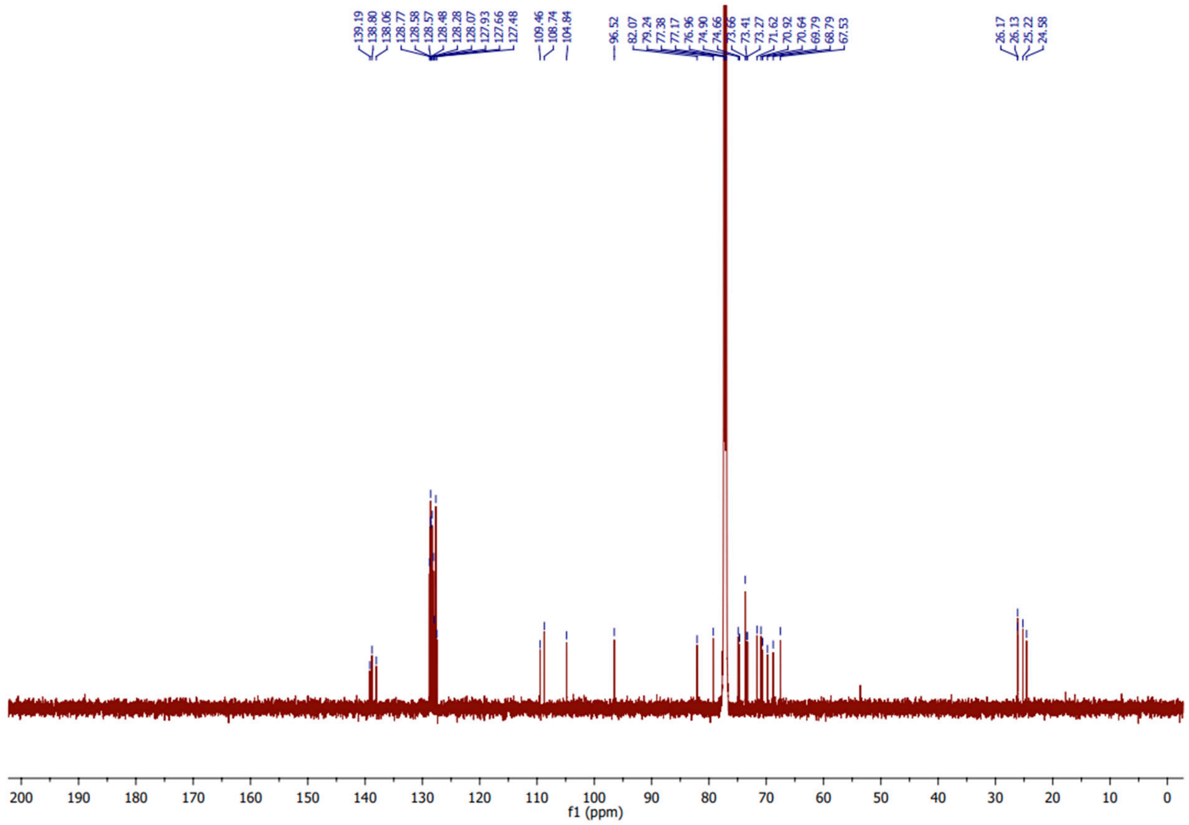

Methyl (2,3,4,6-tetra-*O*-benzyl-*β*-D-galactopyranosyl)-(1à2)-3,4,6-tri-*O*-benzyl-*β*-D-glucopyranoside (14*β*)

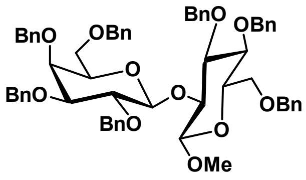

^{1}H NMR (600 MHz, CDCl$_3$)

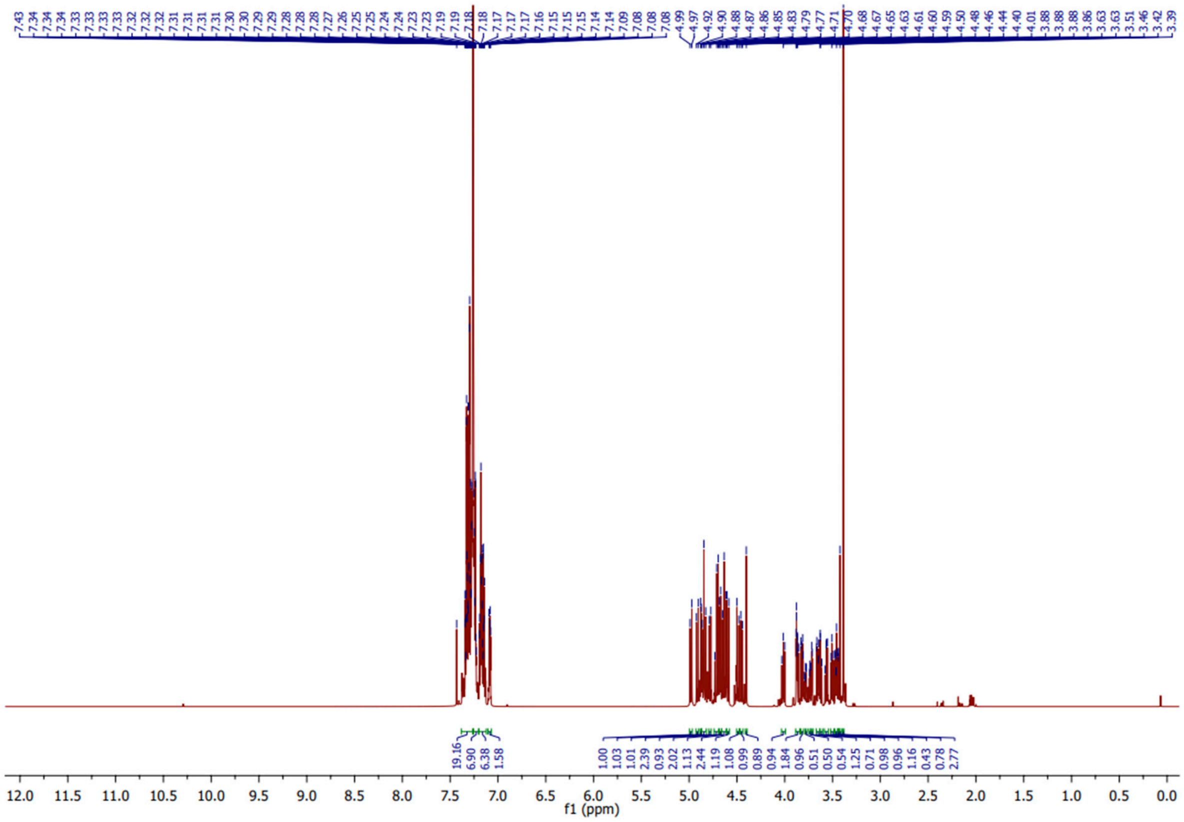

Methyl (2,3,4,6-tetra-*O*-benzyl-*β*-D-galactopyranosyl)-(1à2)-3,4,6-tri-*O*-benzyl-*β*-D-glucopyranoside (14*β*)

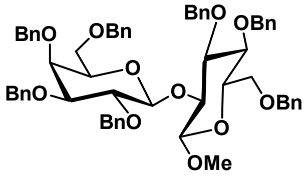

^{13}C NMR (151 MHz, $CDCl_3$)

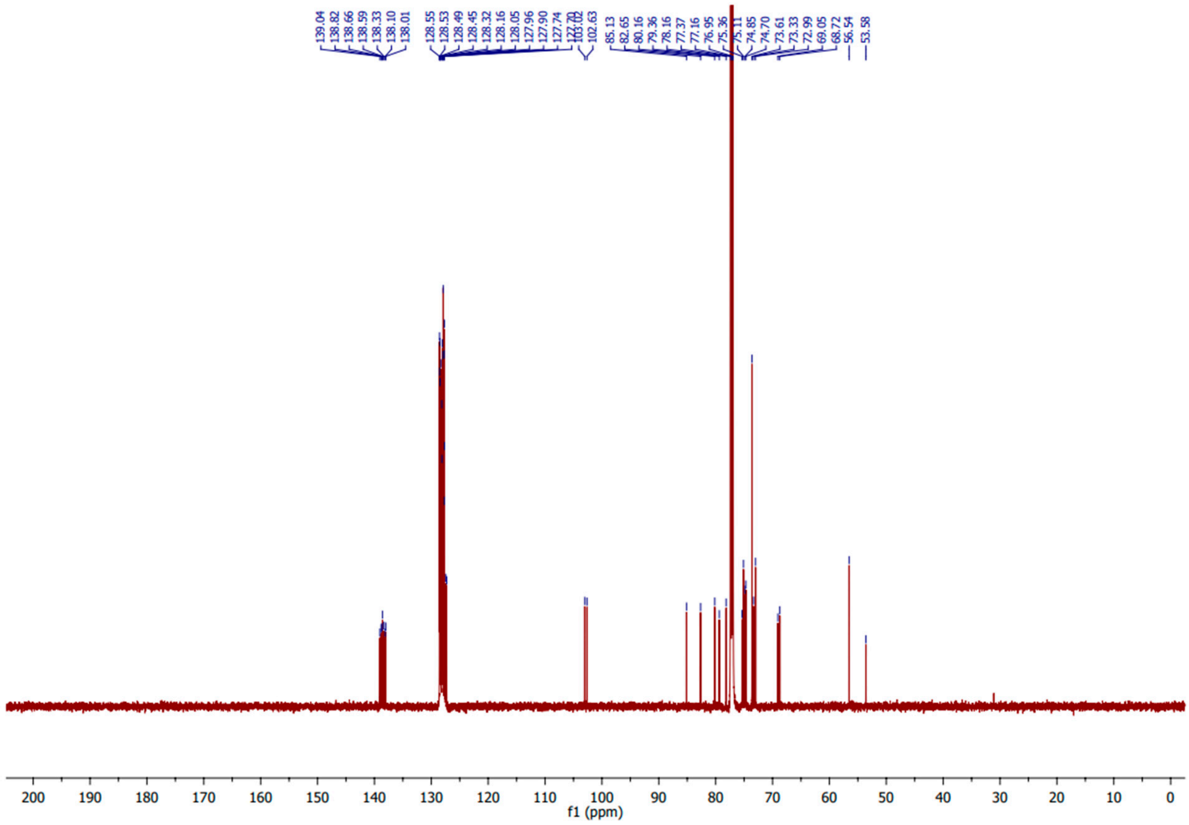

Methyl (2,3,4,6-tetra-*O*-benzyl-*α*-D-galactopyranosyl)-(1à2)-3,4,6-tri-*O*-benzyl-*β*-D-glucopyranoside (14*α*)

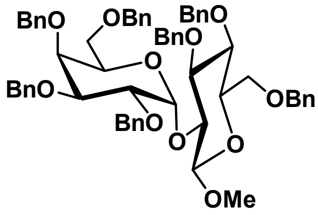

^{1}H NMR (600 MHz, CDCl$_3$)

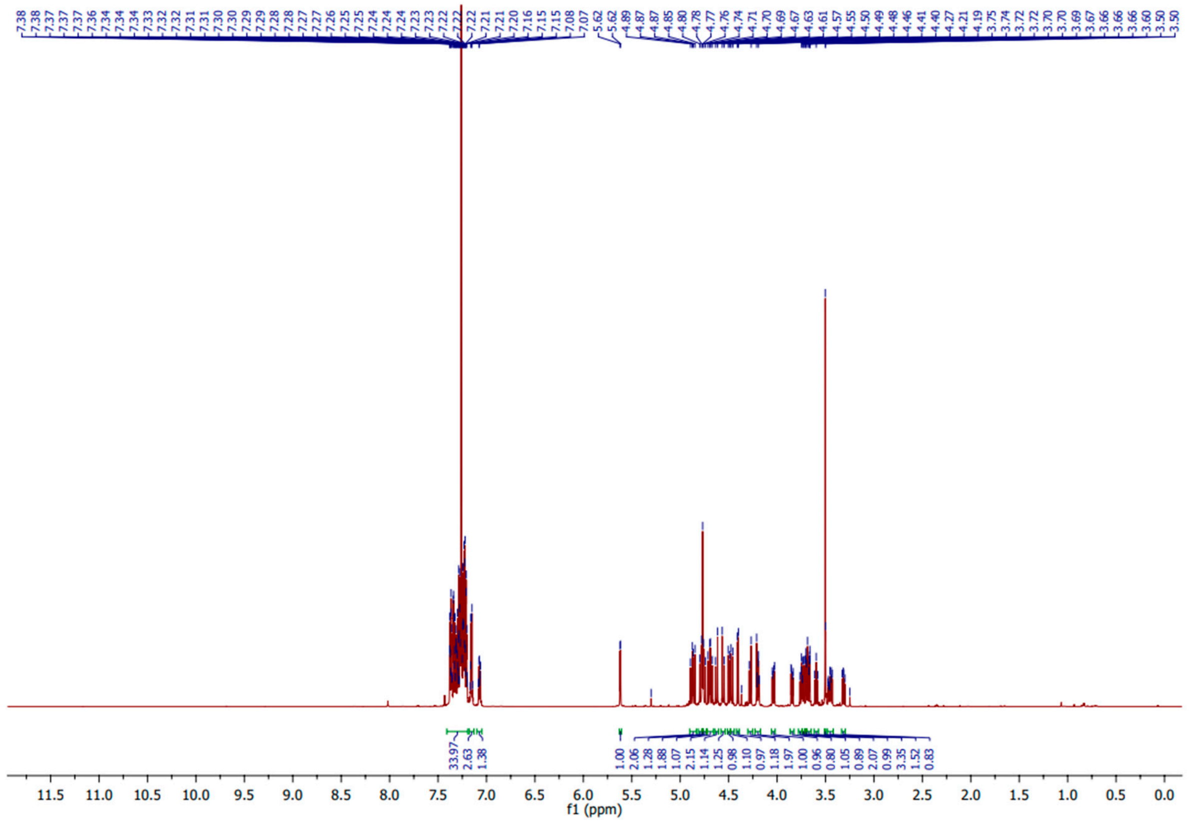

Methyl (2,3,4,6-tetra-*O*-benzyl-*α*-D-galactopyranosyl)-(1à2)-3,4,6-tri-*O*-benzyl-*β*-D-glucopyranoside (14*α*)

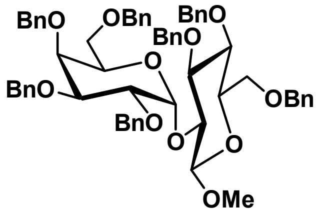

^{13}C NMR (151 MHz, CDCl$_3$)

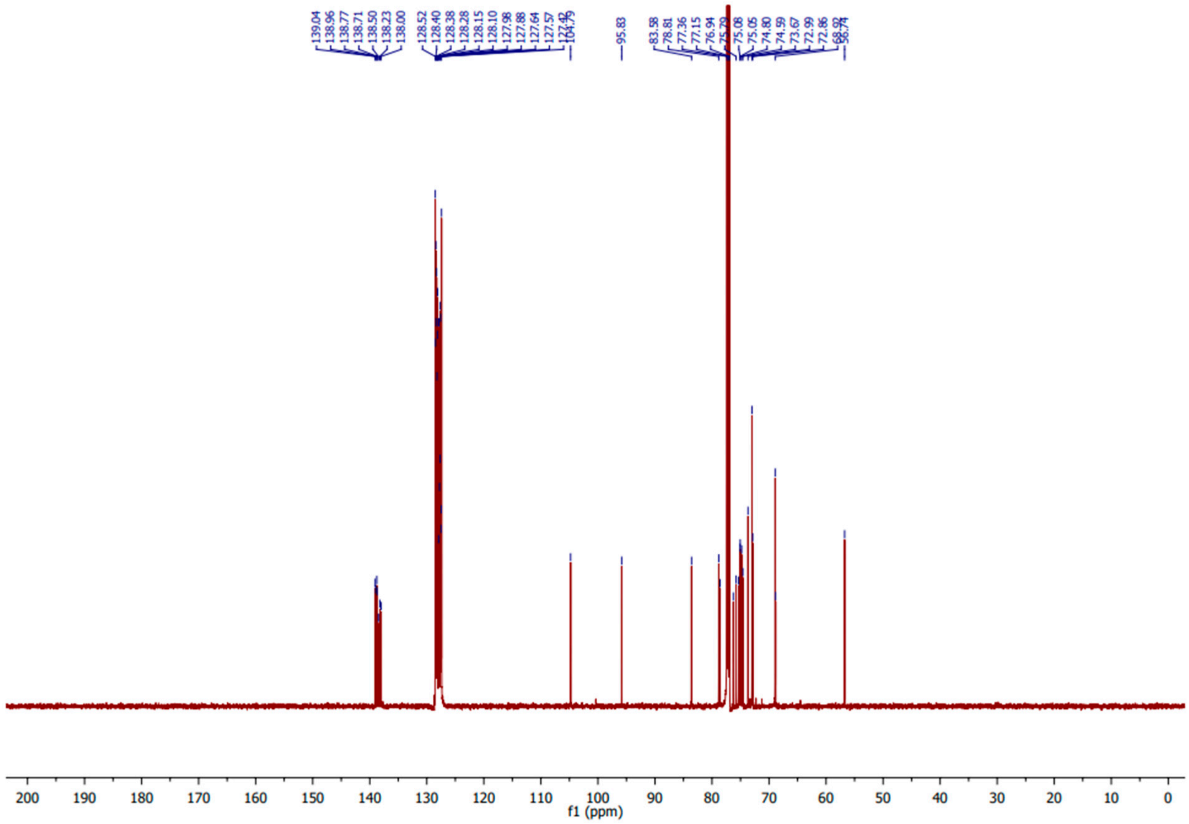

Methyl (2,3,4,6-tetra-O-benzyl-α-D-galactopyranosyl)-(1à3)-2,4,6-tri-*O*-benzyl- β-D-glucopyranoside (15α)

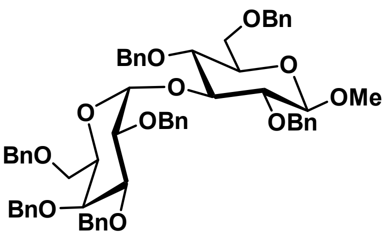

^{1}H NMR (600 MHz, $CDCl_3$)

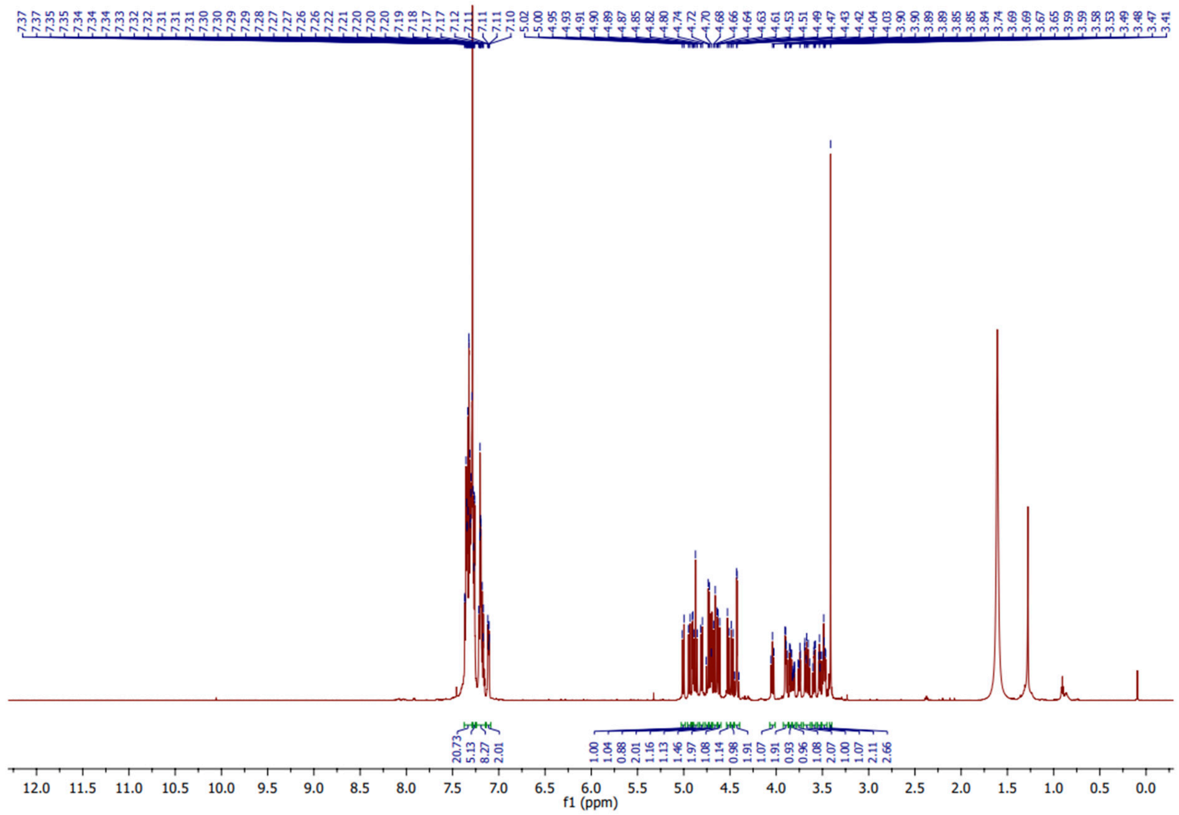

Methyl (2,3,4,6-tetra-O-benzyl-α-D-galactopyranosyl)-(1à3)-2,4,6-tri-*O*-benzyl-β-D-glucopyranoside (15α)

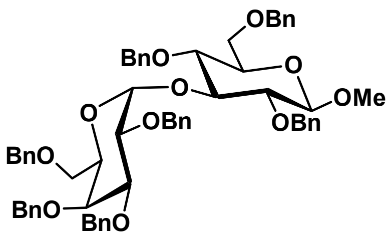

^{13}C NMR (151 MHz, CDCl3)

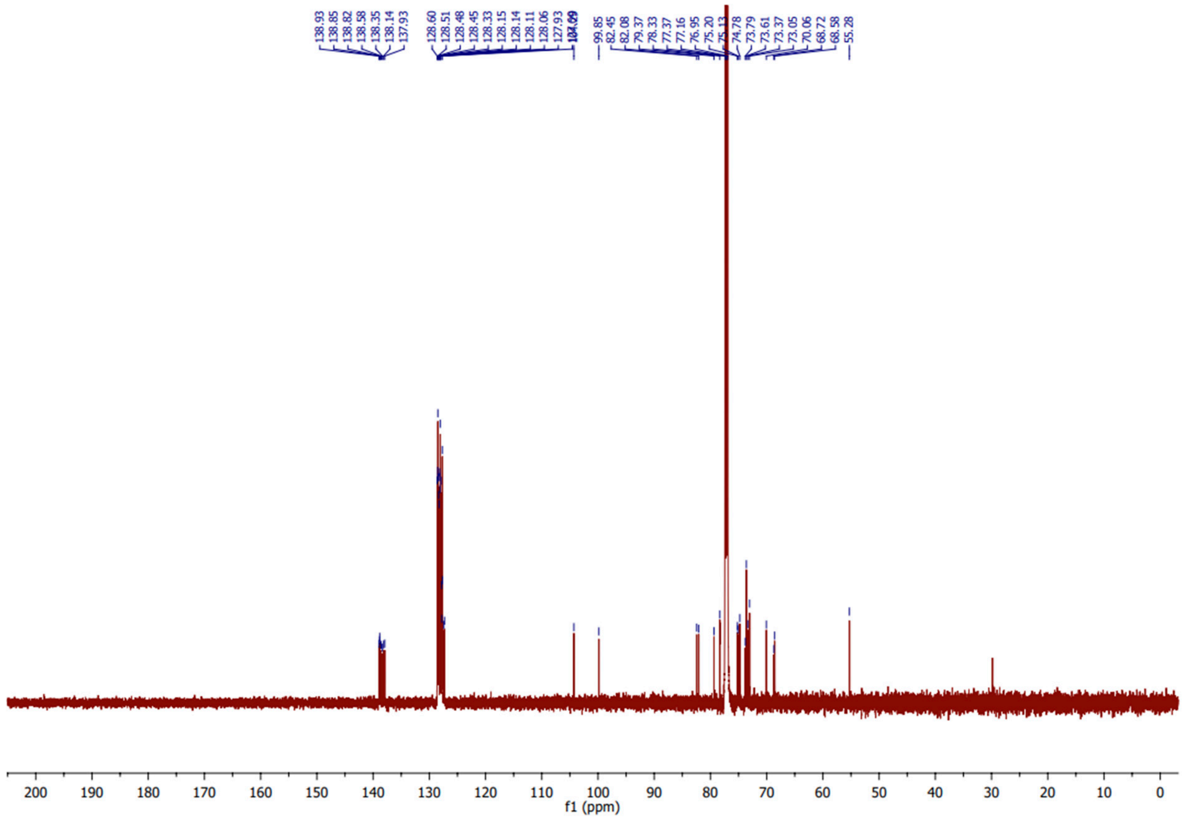

Methyl (2,3,4,6-tetra-*O*-benzyl-β-D-galactopyranosyl)-(1à3)-2,4,6-tri-*O*-benzyl-β-D-glucopyranoside (15*β*)

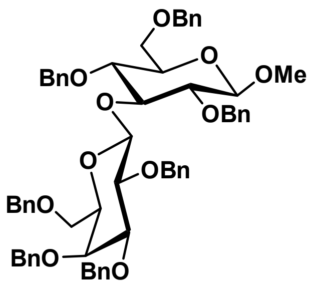

^{1}H NMR (600 MHz, CDCl$_3$)

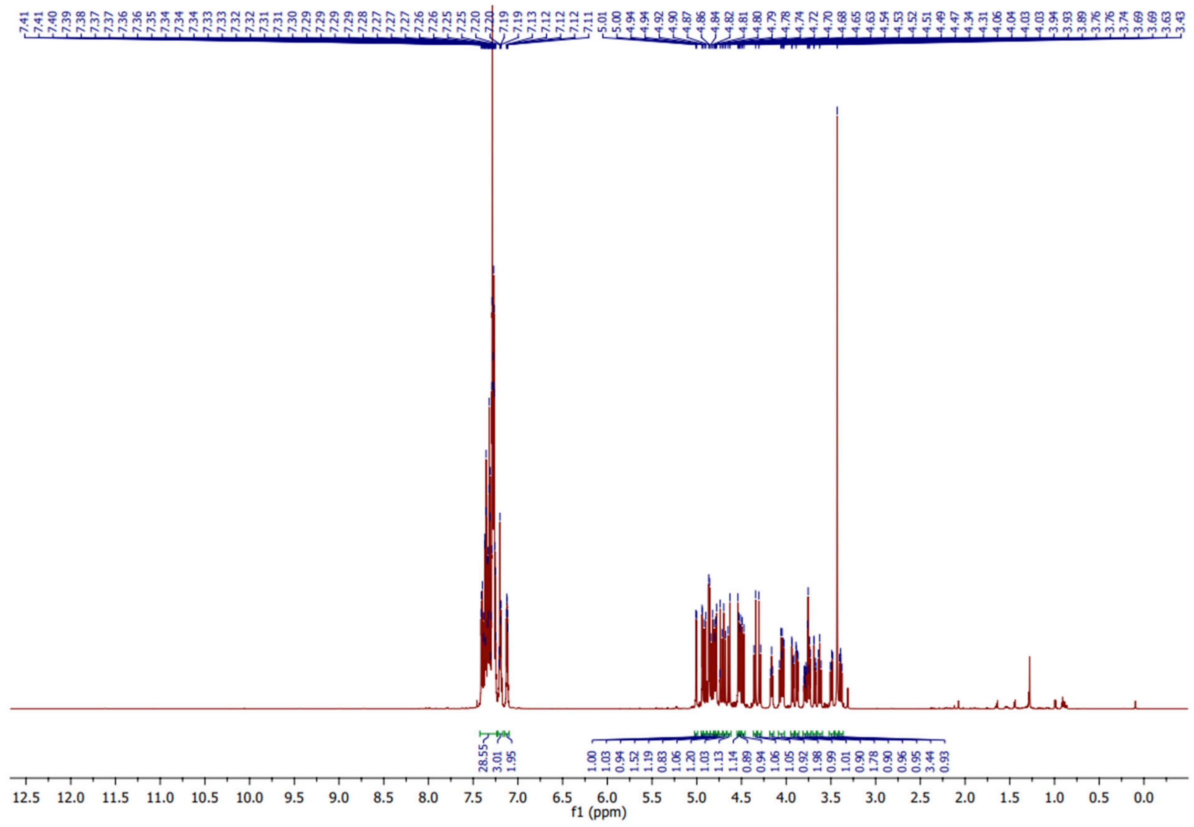

Methyl (2,3,4,6-tetra-*O*-benzyl-β-D-galactopyranosyl)-(1à3)-2,4,6-tri-*O*-benzyl-β-D-glucopyranoside (15*β*)

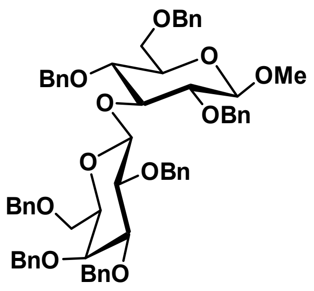

^{13}C NMR (151 MHz, $CDCl_3$)

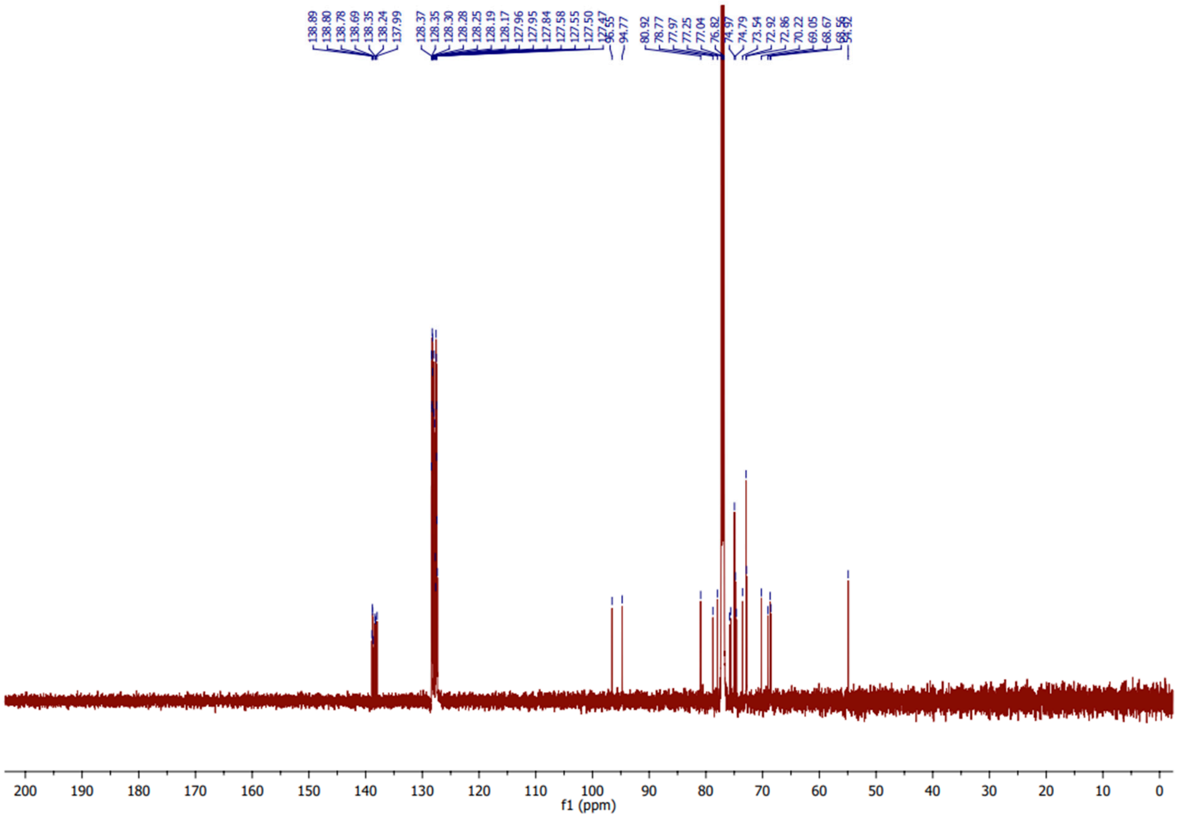

1-Adamantanyl 2,3,4,6-tetra-O-benzyl-α-D-galactopyranoside (17α)

^{1}H NMR

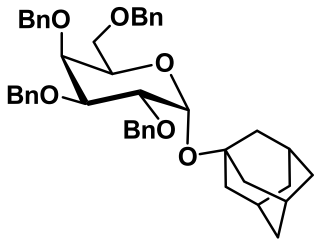

^{1}H NMR (600 MHz, $CDCl_3$)

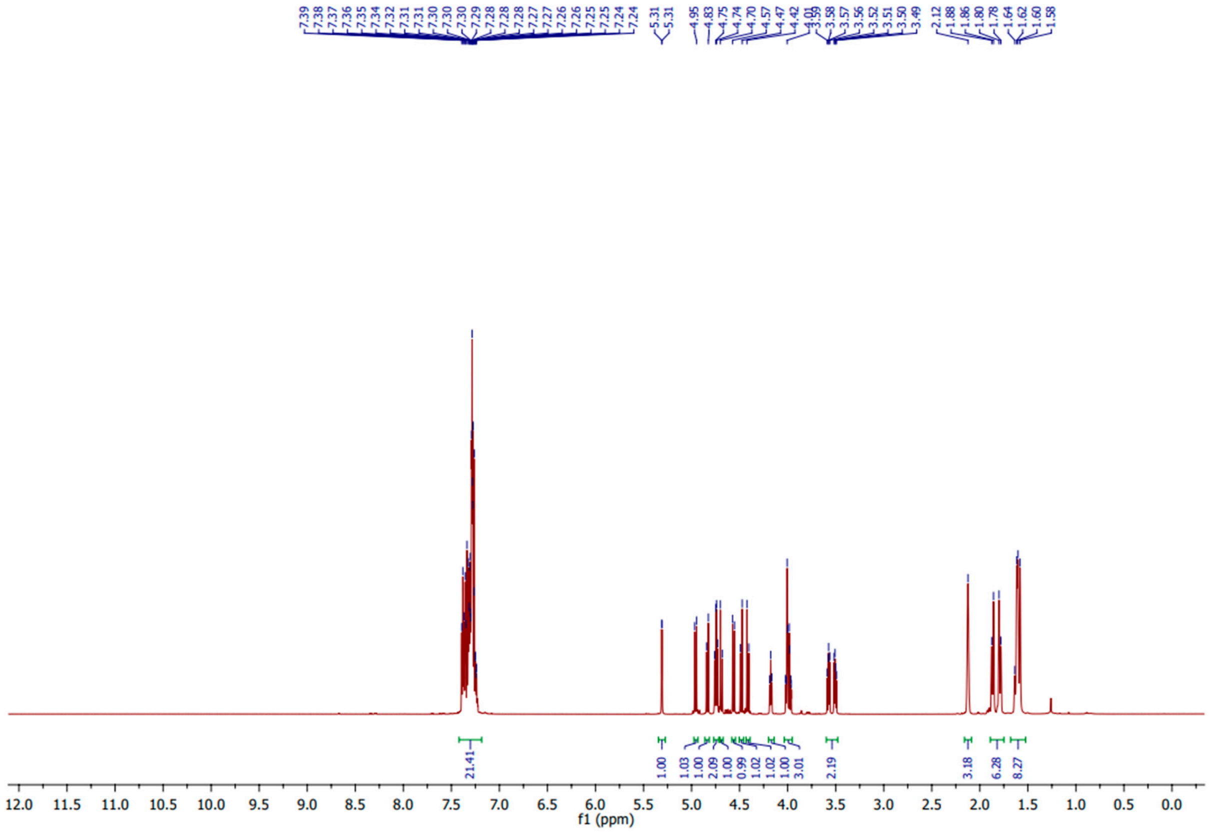

1-Adamantanyl 2,3,4,6-tetra-O-benzyl-α-D-galactopyranoside (17α)

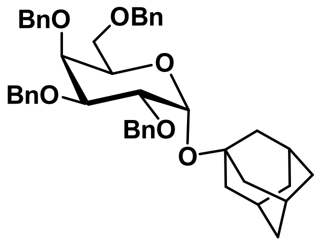

^{13}C NMR (151 MHz, CDCl$_3$)

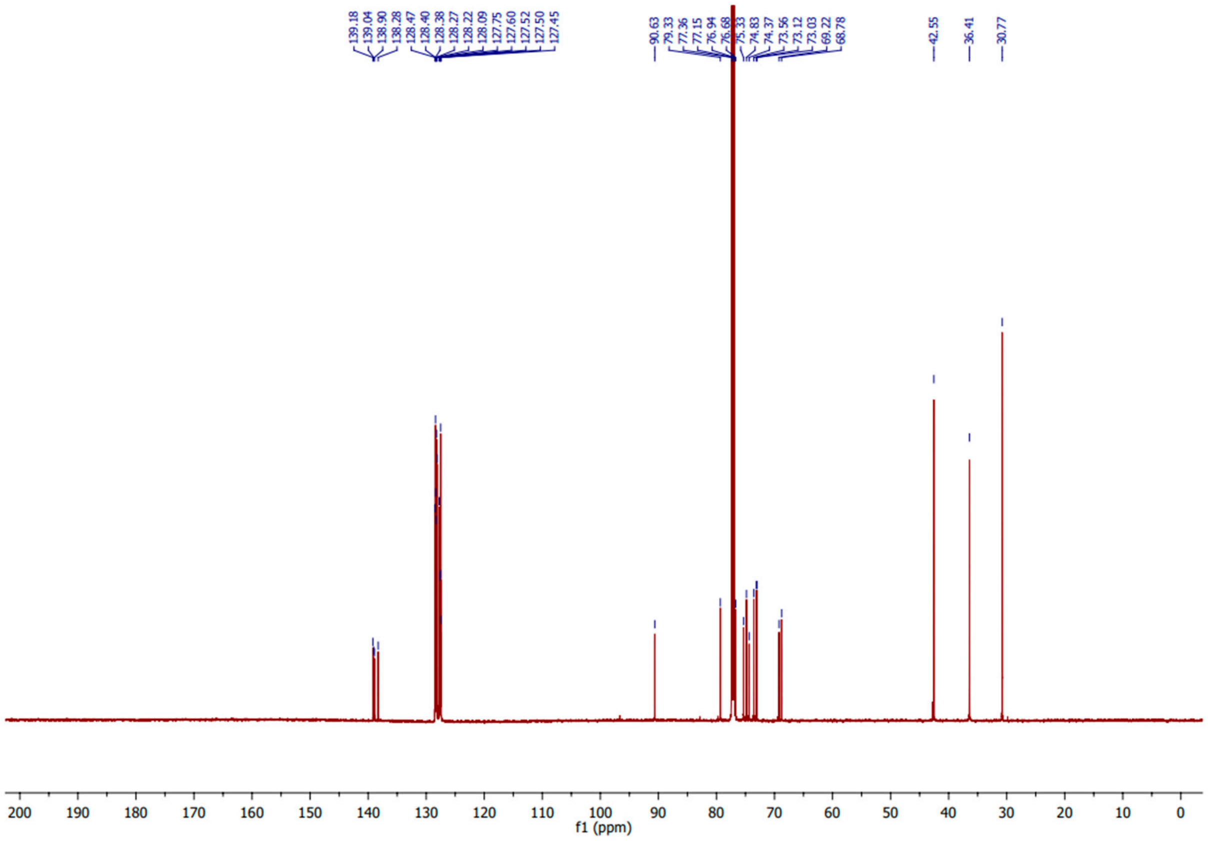

Cholesteryl 2,3,4,6-tetra-O-benzyl-D-glucopyranoside (18 *α*/*β*)

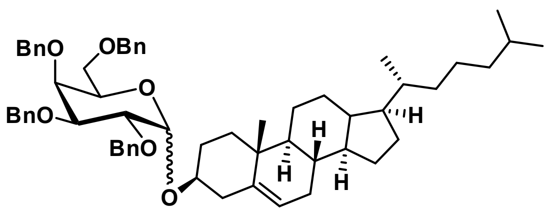

^{1}H NMR (600 MHz, CDCl3)

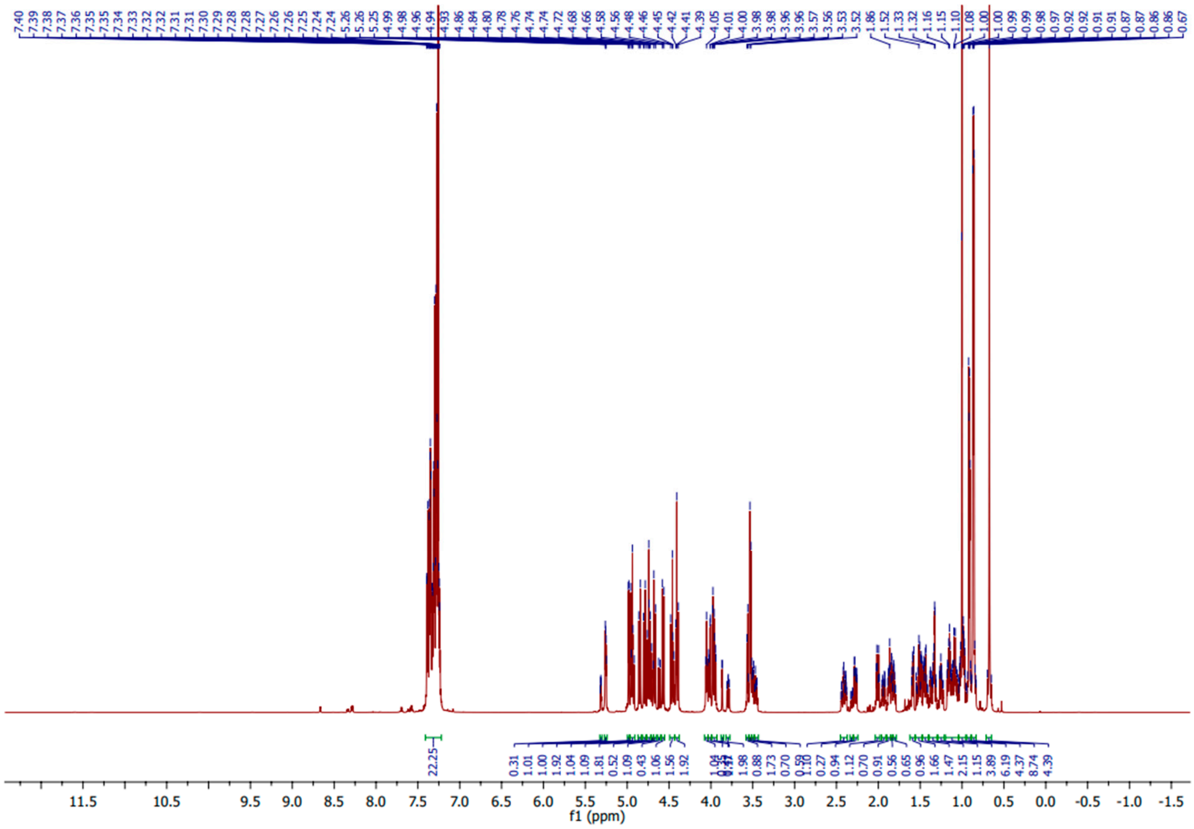

Cholesteryl 2,3,4,6-tetra-O-benzyl-D-glucopyranoside (18 *α*/*β*)

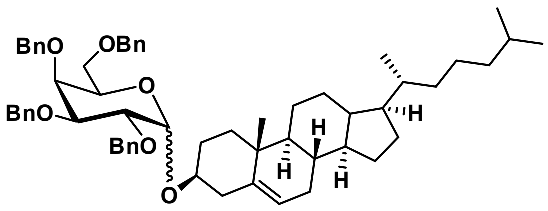

^{13}C NMR (151 MHz, $CDCl_3$)

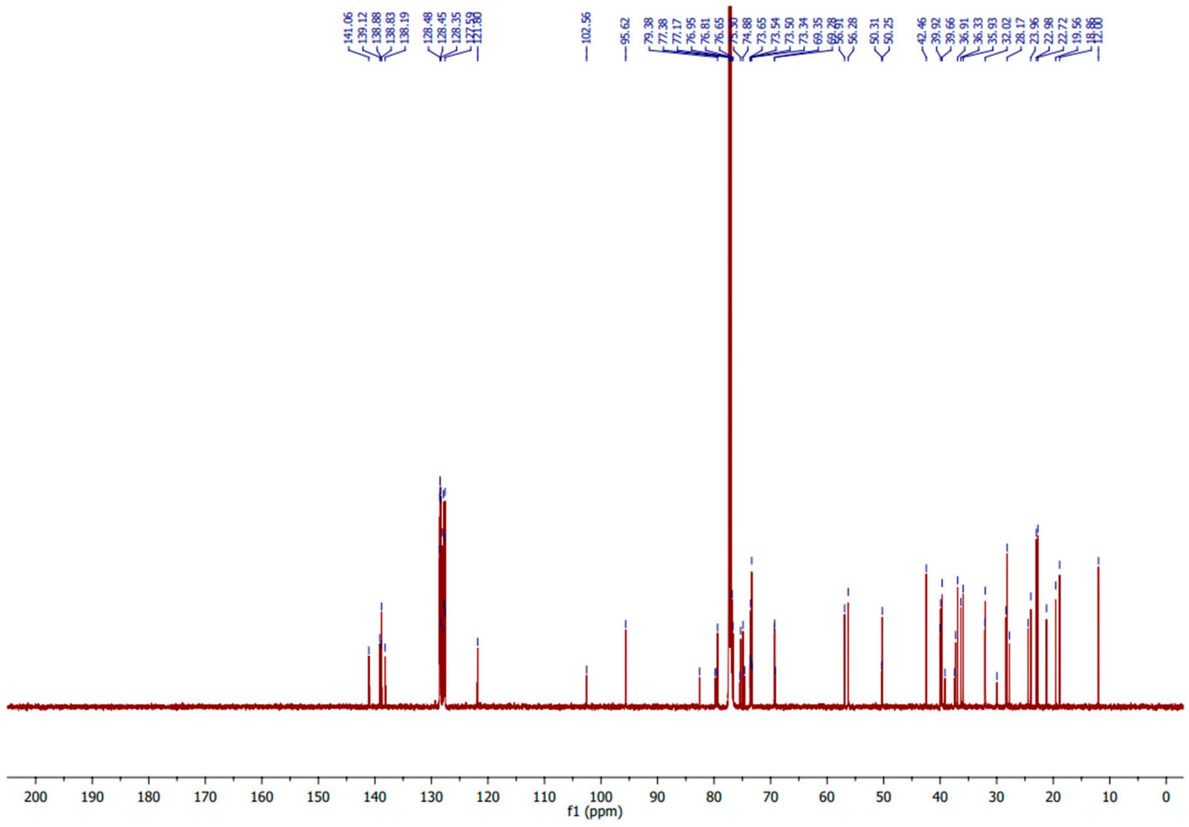

2,4-Dinitrophenyl (2,3,4,6-tetra-*O*-benzyl-*α*-D-galactopyranosyl)-2,3,4-tri-*O*-benzyl-*α*-D galactopyranoside (19a)

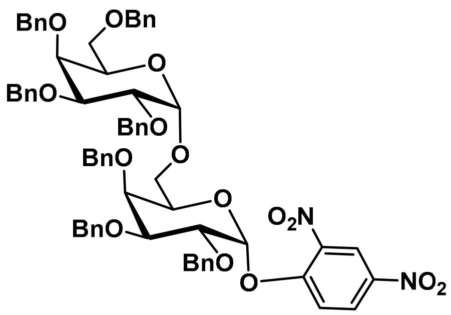

^{1}H NMR (600 MHz, $CDCl_3$)

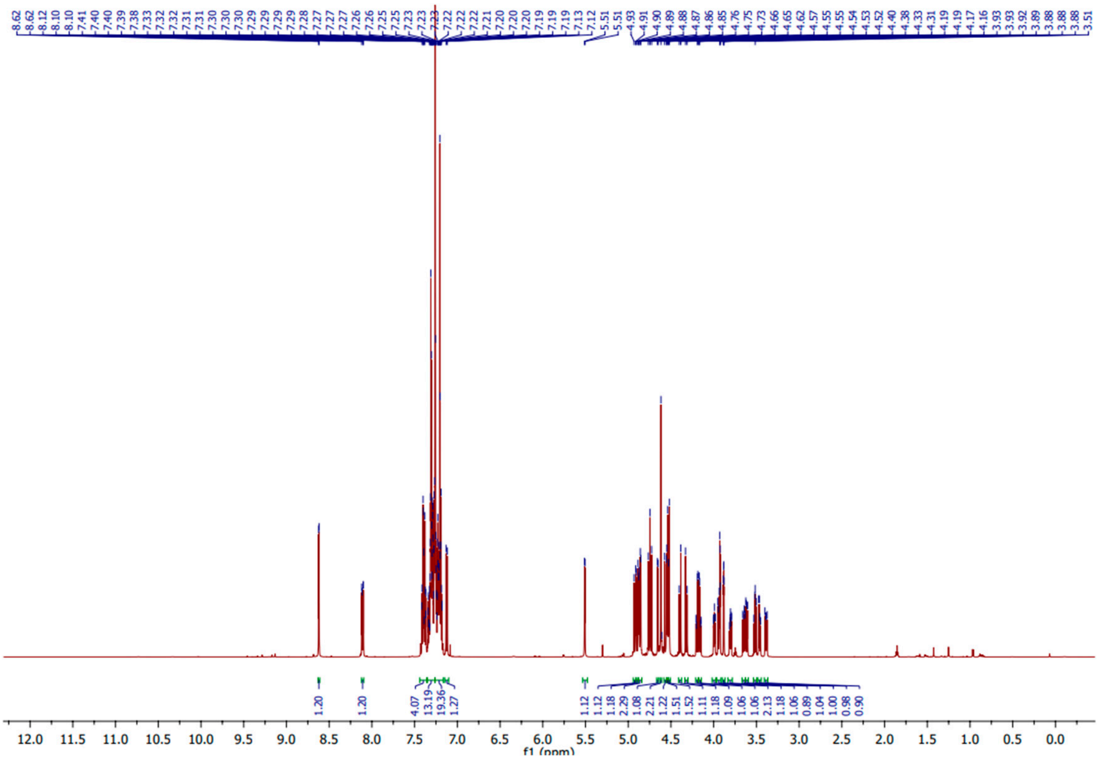

2,4-Dinitrophenyl (2,3,4,6-tetra-*O*-benzyl-*α*-D-galactopyranosyl)-2,3,4-tri-*O*-benzyl-*α*-D galactopyranoside (19a)

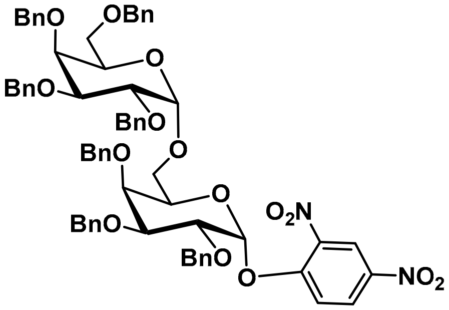

^{13}C NMR (151 MHz, CDCl$_3$)

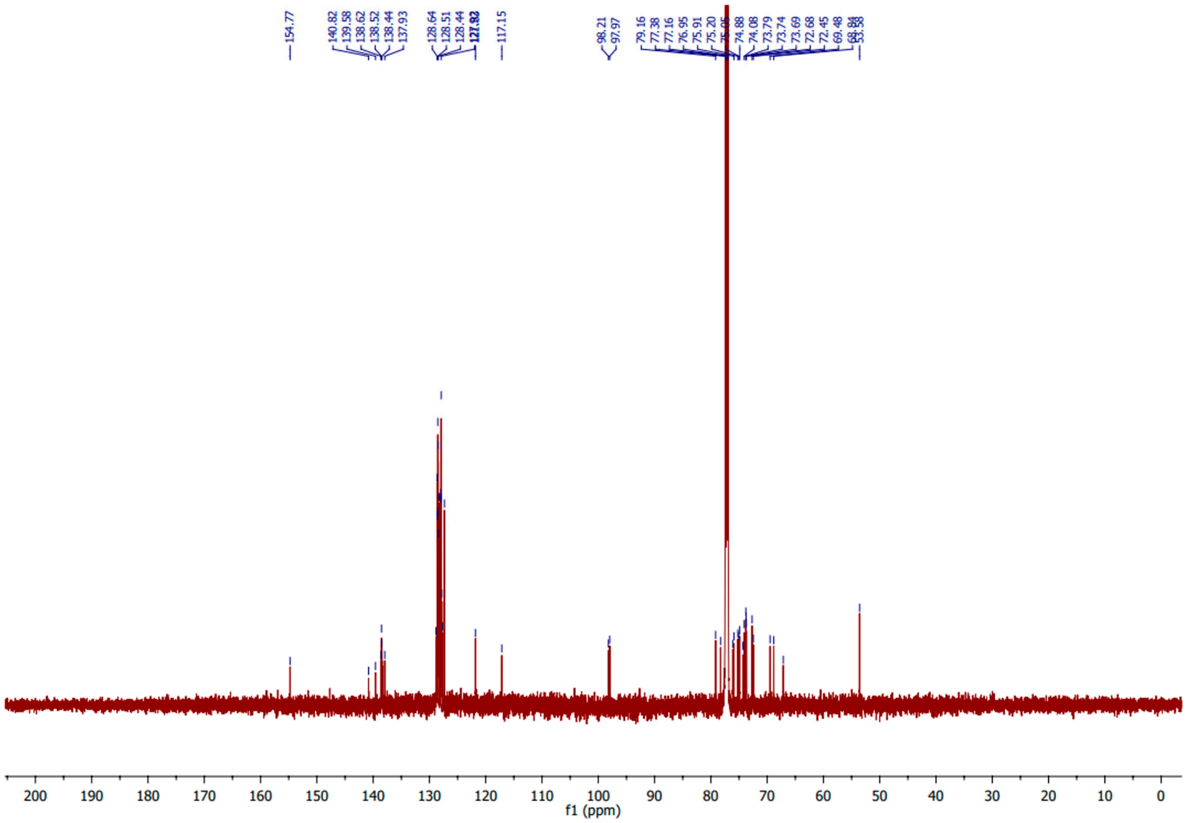

2,4-Dinitrophenyl (2,3,4,6-tetra-*O*-benzyl-*α*-*D*-galactopyranosyl)-2,3,4-tri-*O*-benzyl-*β*-D-galactopyranoside (19b)

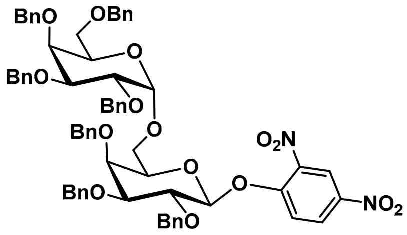

^{1}H NMR (600 MHz, CDCl$_3$)

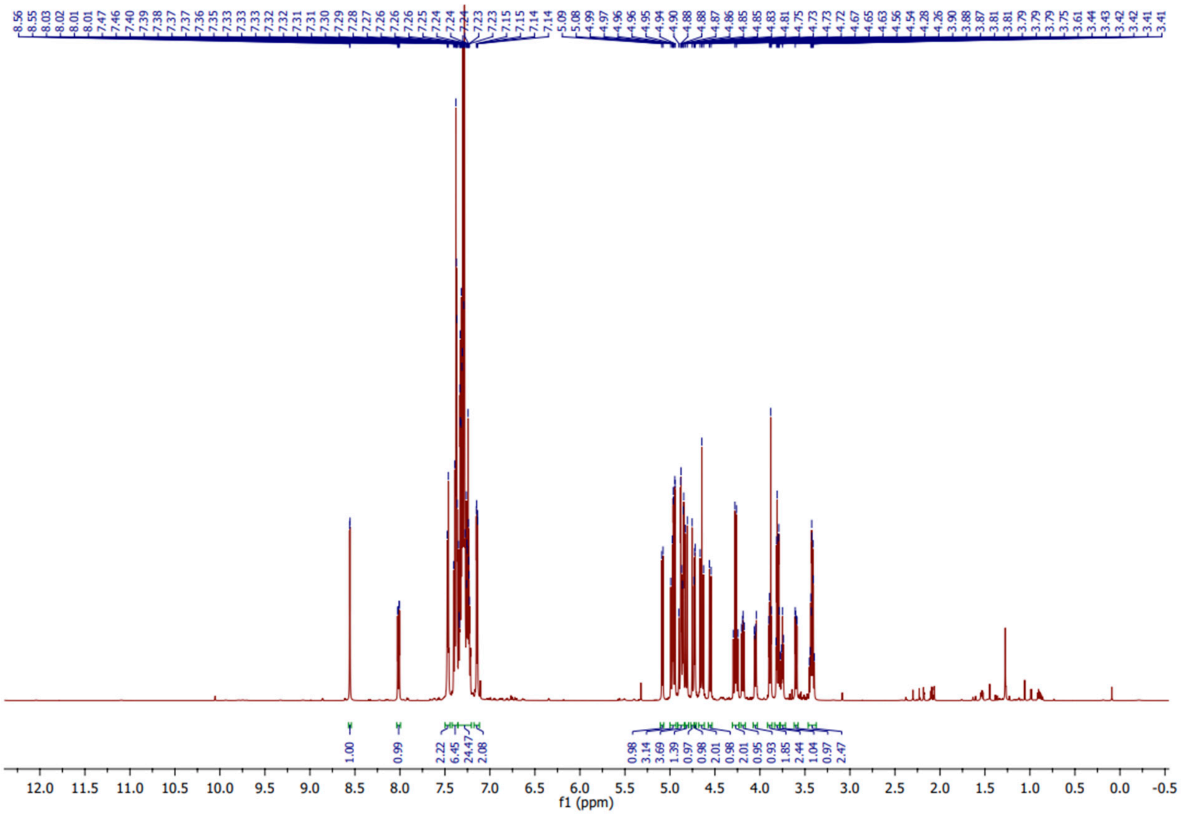

2,4-Dinitrophenyl (2,3,4,6-tetra-*O*-benzyl-*α*-*D*-galactopyranosyl)-2,3,4-tri-*O*-benzyl-*β*-D-galactopyranoside (19b)

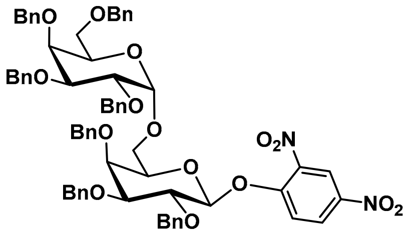

^{13}C NMR (151 MHz, CDCl₃)

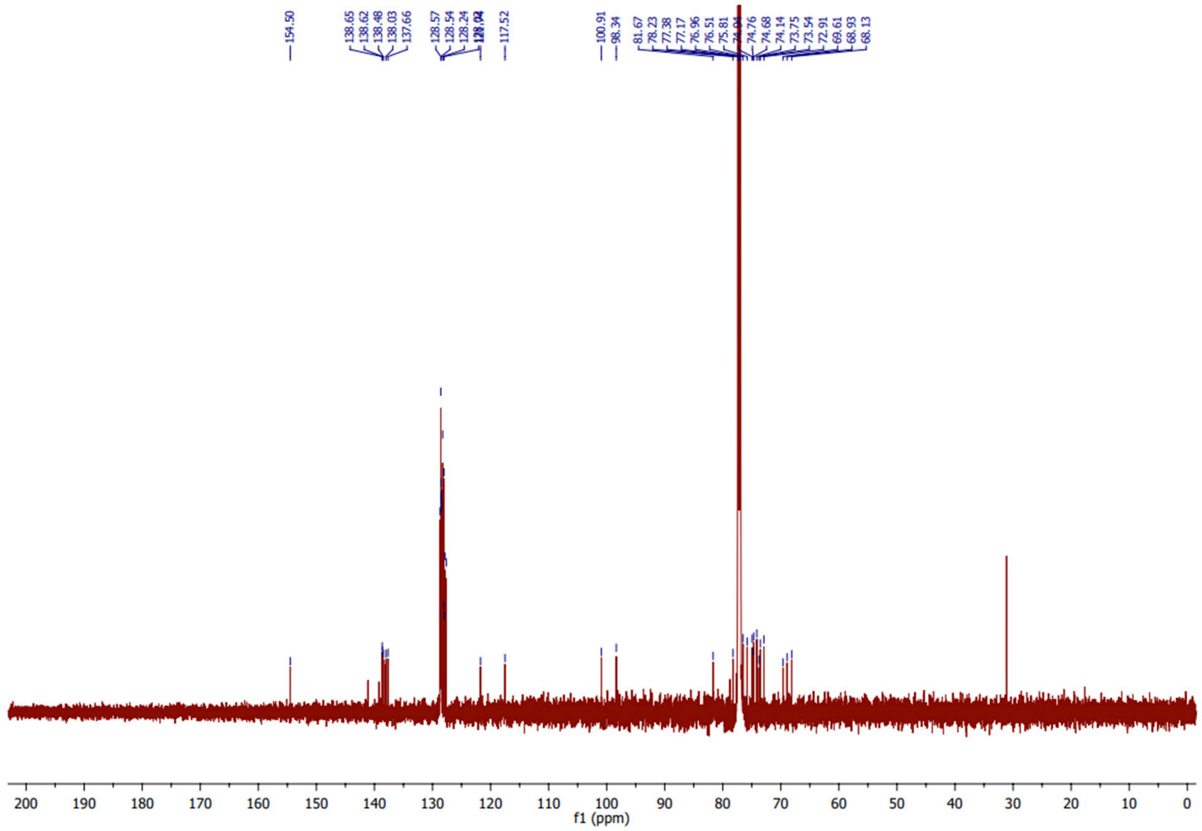

Methyl 2,3,4-tri-O-benzyl-6-O-[2,3,4-tri-O-benzyl-6-O-(2,3,4,6-tetra-O-benzyl-α-D-galactopyranosyl)-α-D-galactopyranosyl]- α -D-galactopyranoside (20)

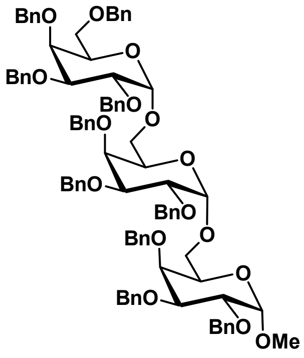

^{1}H NMR (600 MHz, $CDCl_3$)

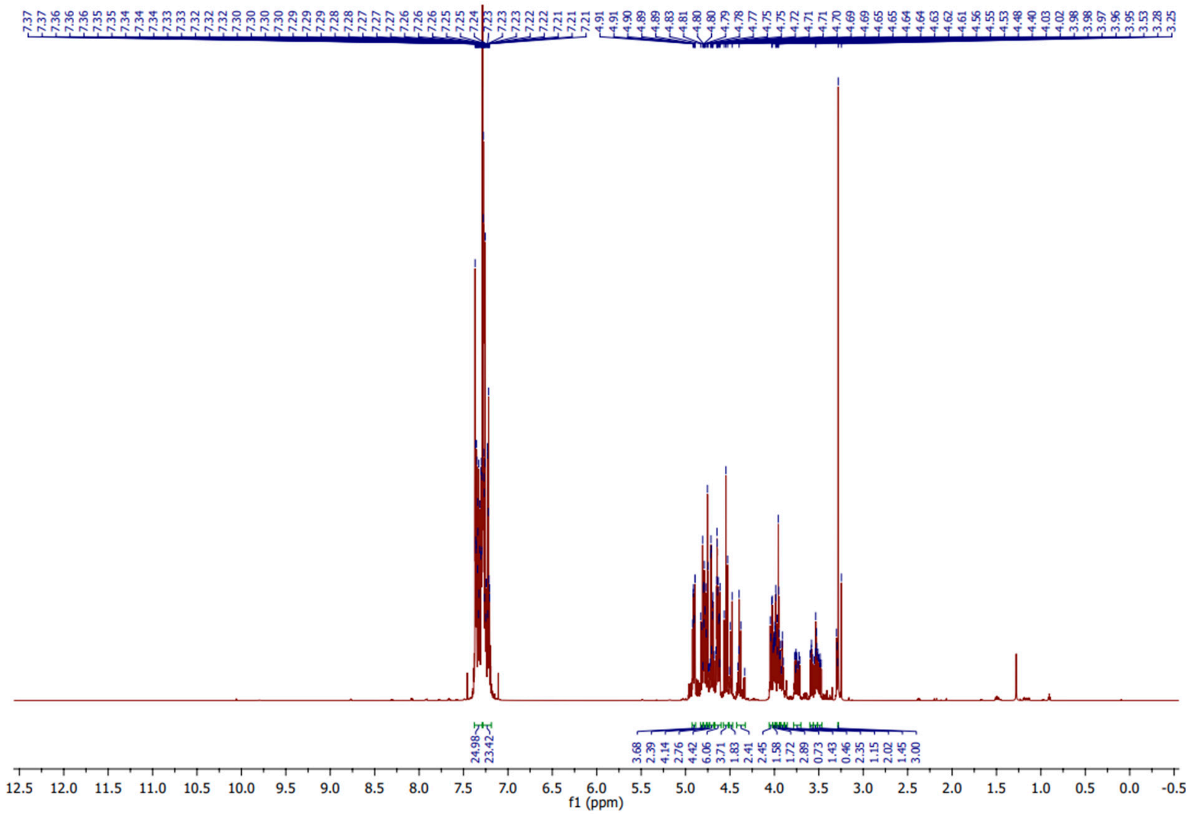

Methyl 2,3,4-tri-O-benzyl-6-O-[2,3,4-tri-O-benzyl-6-O-(2,3,4,6-tetra-O-benzyl-α-D-galactopyranosyl)-α-D-galactopyranosyl]- α -D-galactopyranoside (20)

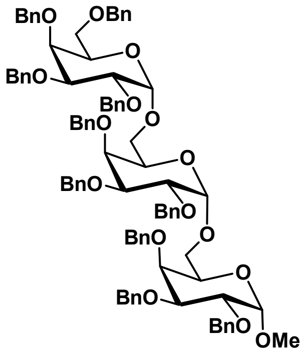

^{13}C NMR (151 MHz, $CDCl_3$)

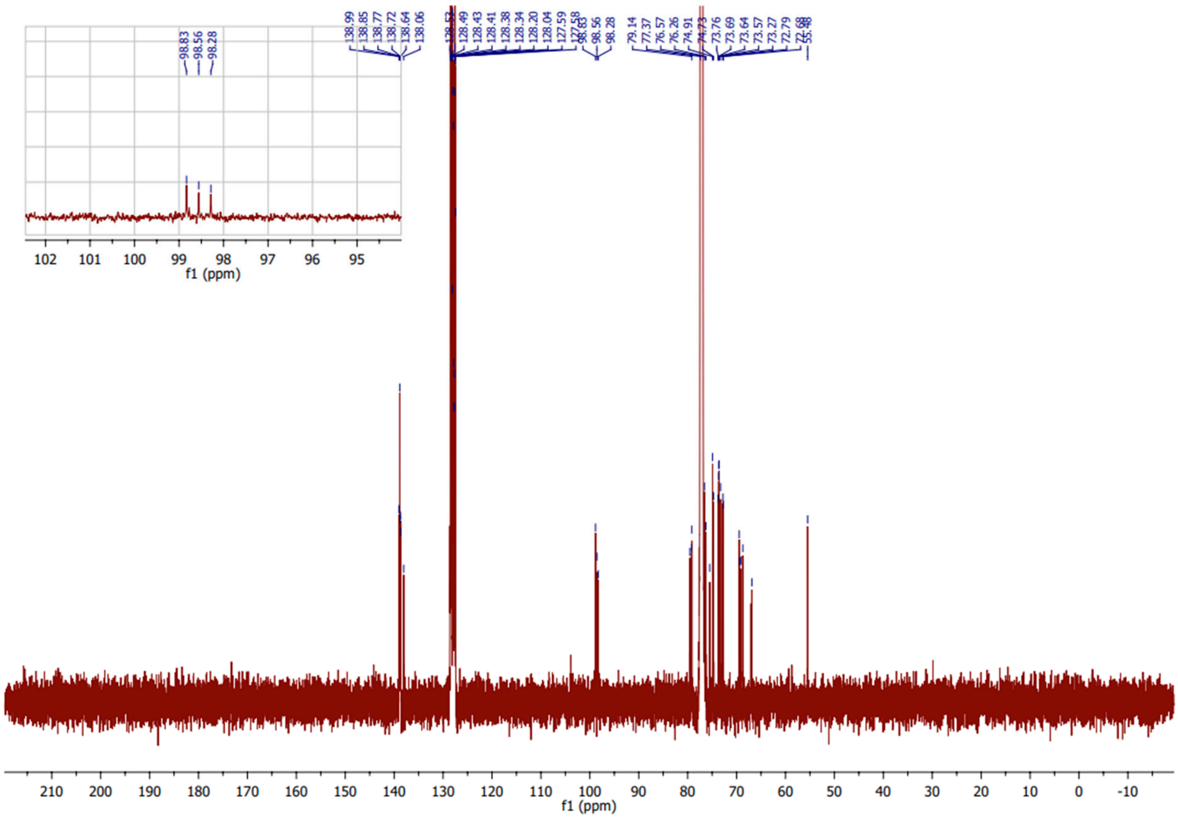

Methyl 2,3,4-tri-O-benzyl-6-O-[2,3,4-tri-O-benzyl-6-O-(2,3,4,6-tetra-O-benzyl-α-D-galactopyranosyl)-α-D-galactopyranosyl]- α -D-galactopyranoside (20)

COSY

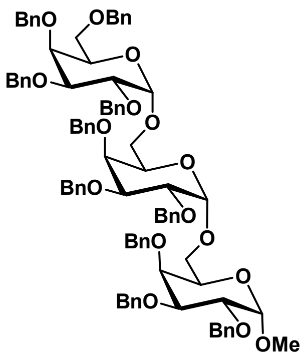

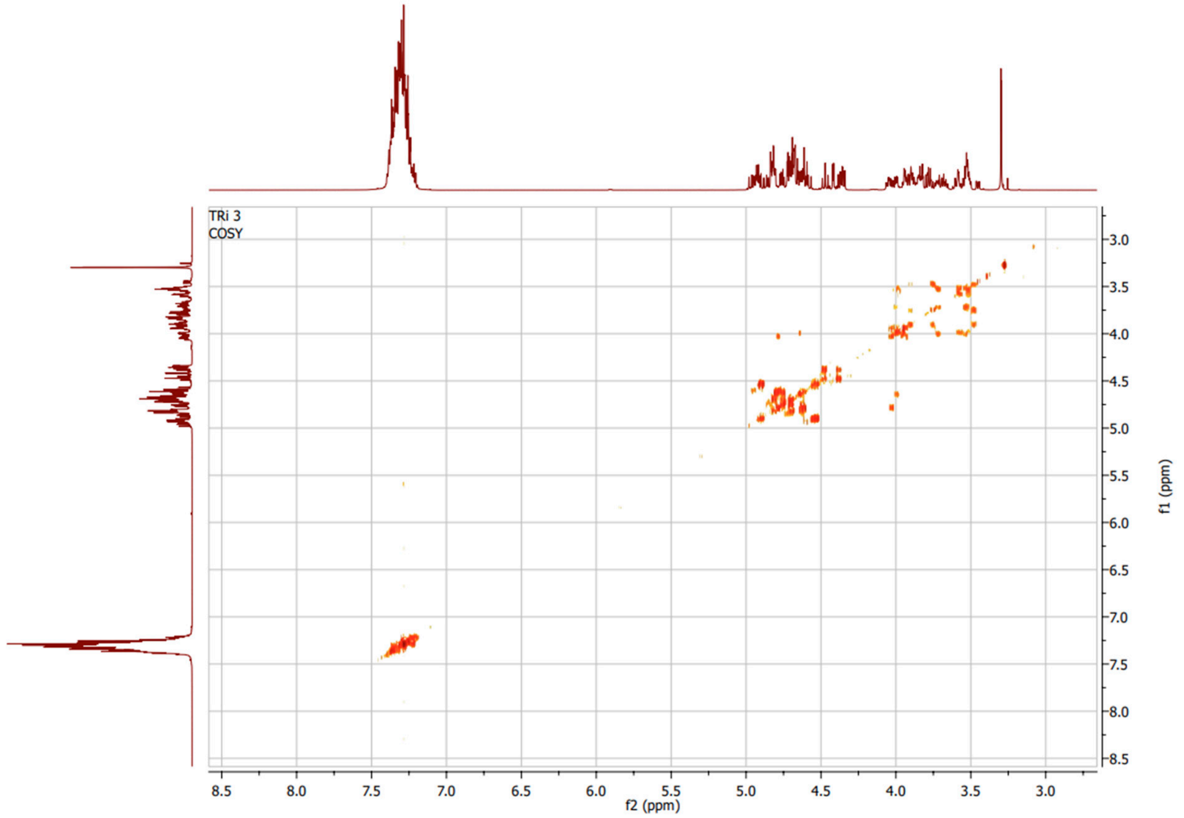

Methyl 2,3,4-tri-O-benzyl-6-O-[2,3,4-tri-O-benzyl-6-O-(2,3,4,6-tetra-O-benzyl-α-D-galactopyranosyl)-α-D-galactopyranosyl]- α -D-galactopyranoside (20)

HSQC

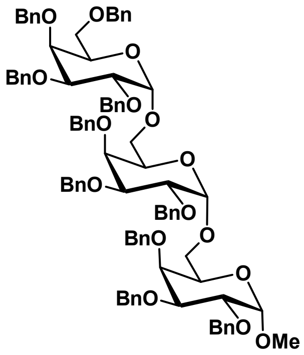

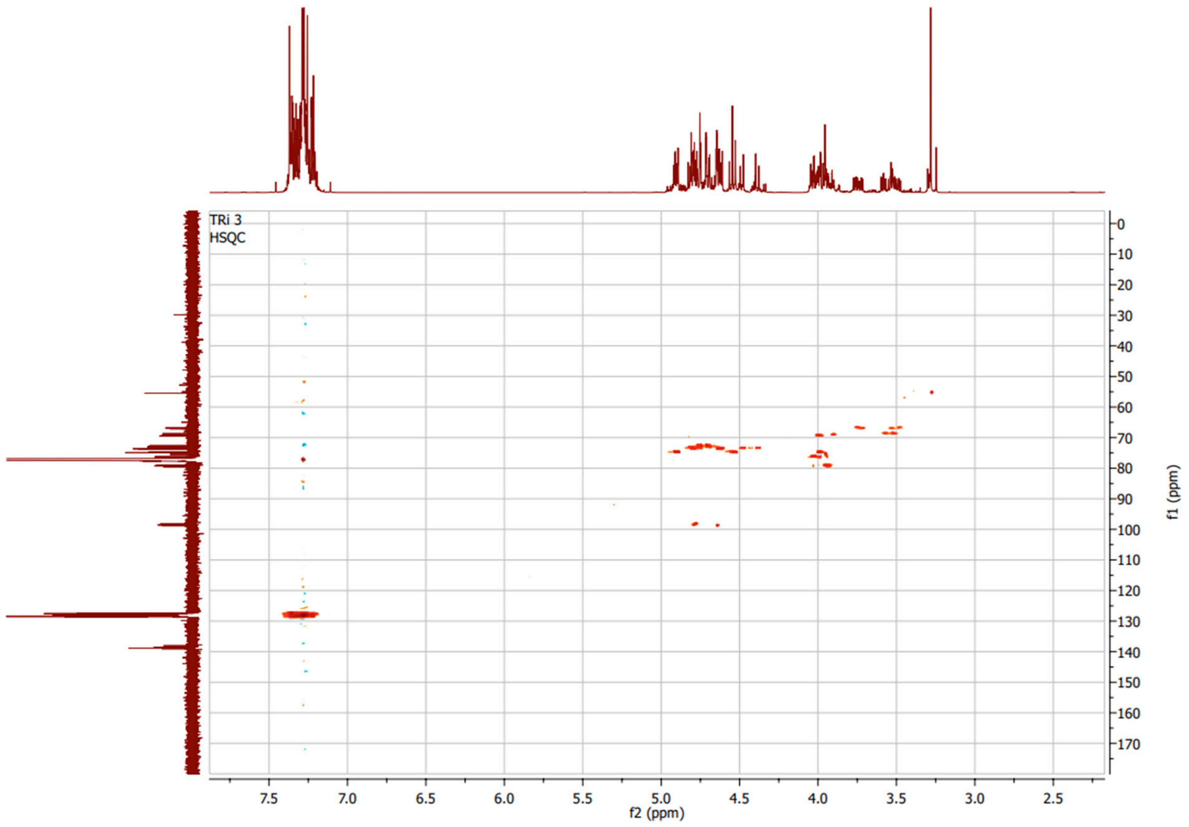

2,3,4,6-Tetra-O-benzyl-D-galactopyranose

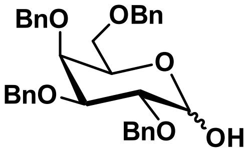

(z)

^{1}H NMR (600 MHz, $CDCl_3$)

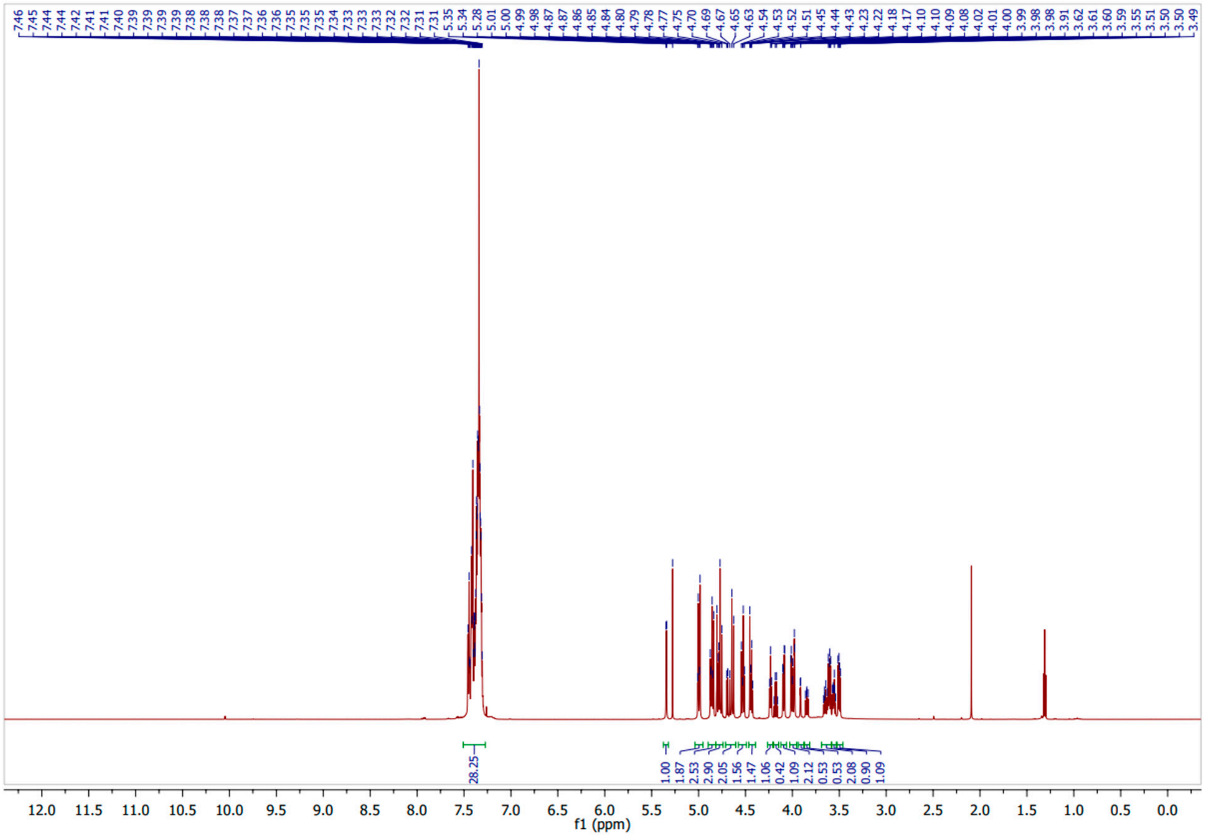

2,3,4,6-Tetra-O-benzyl-D-galactopyranose (z)

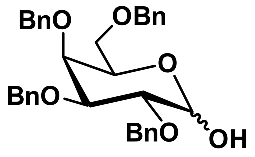

(z)

^{13}C NMR (151 MHz, $CDCl_3$)

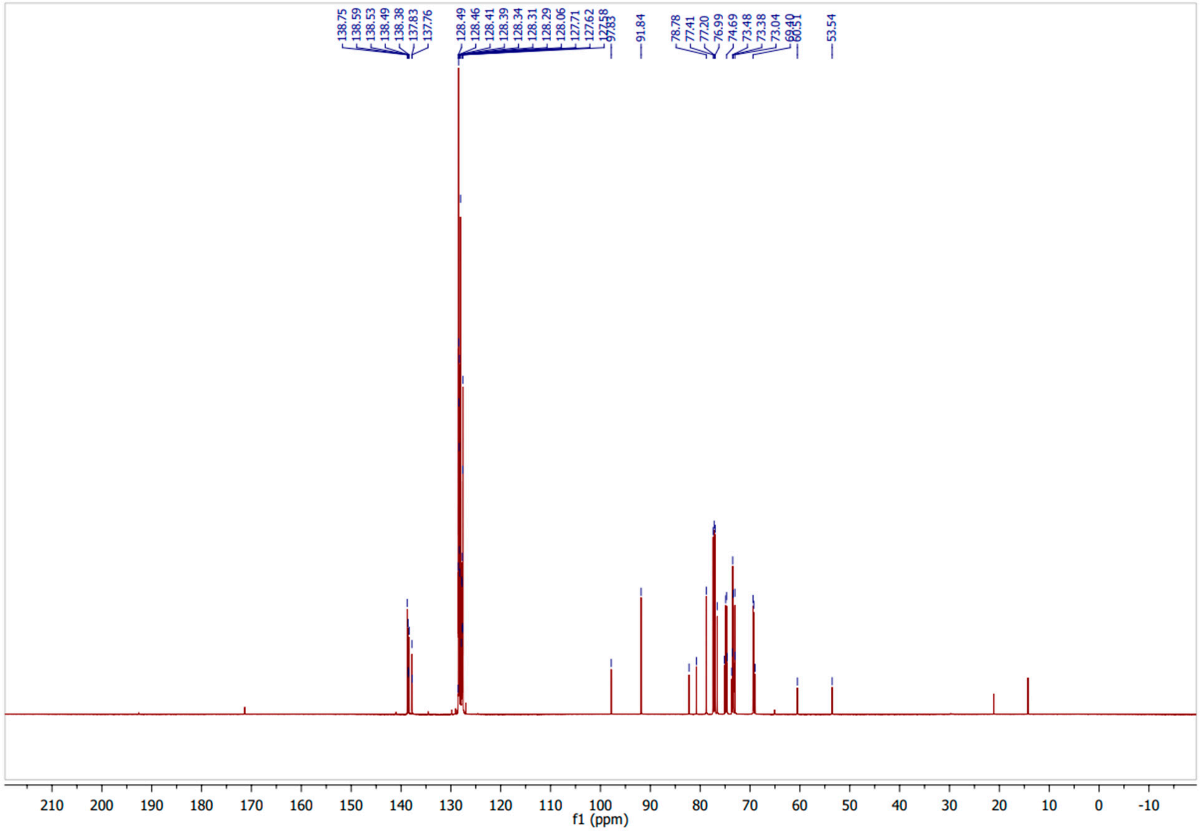

2,3,4,6-tetra-*O*-benzyl-D-galactal (y)

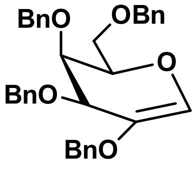

(y)

^{1}H NMR (600 MHz, $CDCl_3$)

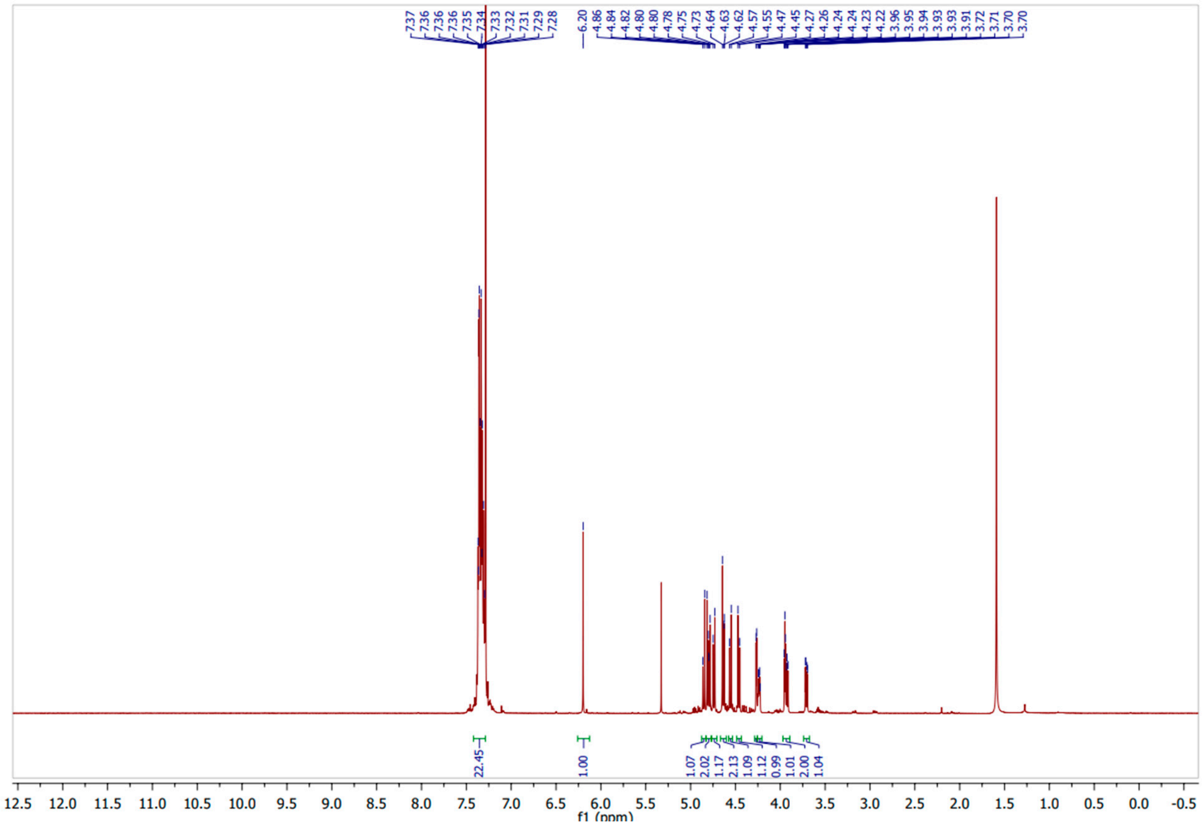

2,4-Dinitrophenol (x)

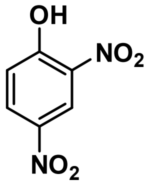

(x)

^{1}H NMR (600 MHz, $CDCl_3$)

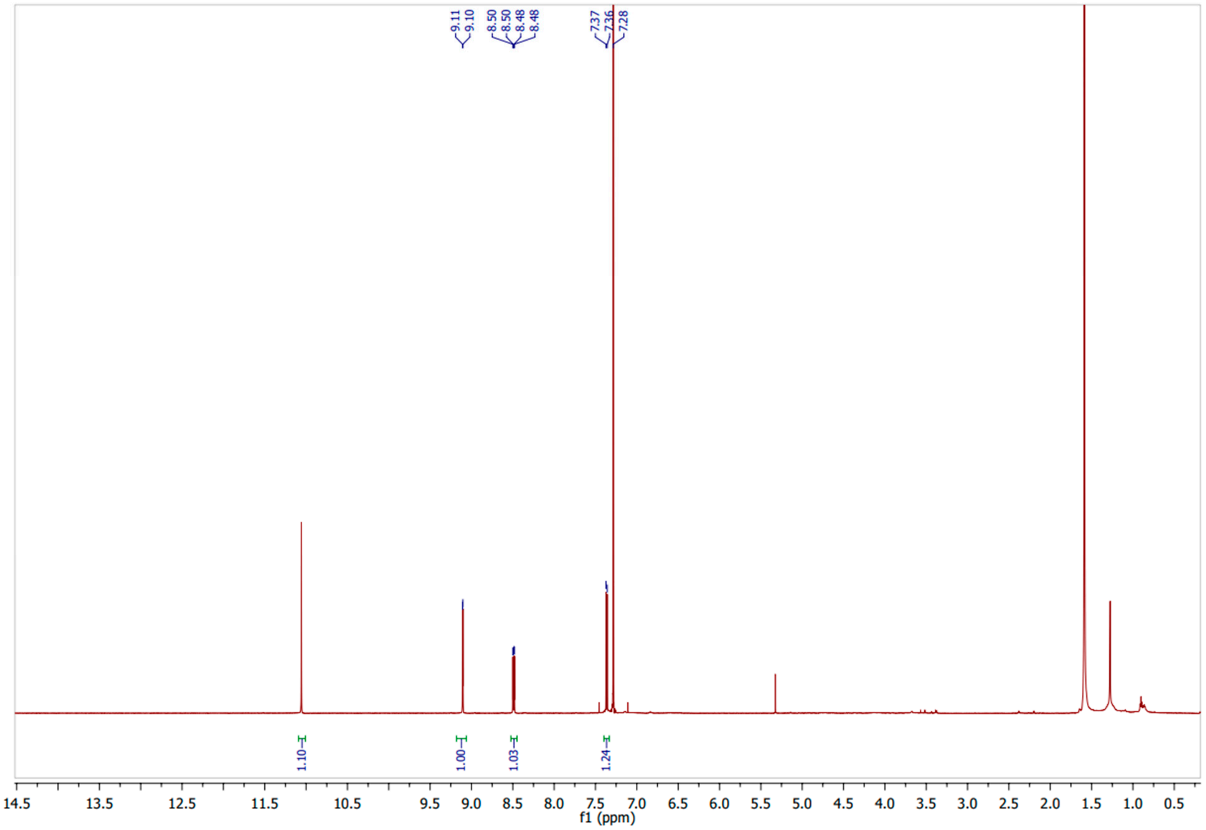

Supplement: Supplementary file 1 [file molecules-30-03693-s001.zip › molecules-3661063-supplementary.pdf]
